# Biological Role of the Intercellular Transfer of Glycosylphosphatidylinositol-Anchored Proteins: Stimulation of Lipid and Glycogen Synthesis

**DOI:** 10.3390/ijms23137418

**Published:** 2022-07-04

**Authors:** Günter A. Müller, Timo D. Müller

**Affiliations:** 1 Institute for Diabetes and Obesity (IDO), Helmholtz Diabetes Center (HDC) at Helmholtz Zentrum München, German Research Center for Environmental Health (GmbH), 85764 Oberschleissheim, Germany; timo.mueller@helmholtz-muenchen.de; 2 German Center for Diabetes Research (DZD, Deutsches Zentrum für Diabetesforschung), International Helmholtz Research School for Diabetes, 85764 Oberschleissheim, Germany

**Keywords:** diabetes, glycosylphosphatidylinositol (GPI)-anchored proteins (GPI-APs), (G)PI-specific phospholipases, phosphoinositolglycans, glycogen and lipid synthesis, protein transfer

## Abstract

Glycosylphosphatidylinositol-anchored proteins (GPI-APs), which are anchored at the outer leaflet of plasma membranes (PM) only by a carboxy-terminal GPI glycolipid, are known to fulfill multiple enzymic and receptor functions at the cell surface. Previous studies revealed that full-length GPI-APs with the complete GPI anchor attached can be released from and inserted into PMs in vitro. Moreover, full-length GPI-APs were recovered from serum, dependent on the age and metabolic state of rats and humans. Here, the possibility of intercellular control of metabolism by the intercellular transfer of GPI-APs was studied. Mutant K562 erythroleukemia (EL) cells, mannosamine-treated human adipocytes and methyl-ß-cyclodextrin-treated rat adipocytes as acceptor cells for GPI-APs, based on their impaired PM expression of GPI-APs, were incubated with full-length GPI-APs, prepared from rat adipocytes and embedded in micelle-like complexes, or with EL cells and human adipocytes with normal expression of GPI-APs as donor cells in transwell co-cultures. Increases in the amounts of full-length GPI-APs at the PM of acceptor cells as a measure of their transfer was assayed by chip-based sensing. Both experimental setups supported both the transfer and upregulation of glycogen (EL cells) and lipid (adipocytes) synthesis. These were all diminished by serum, serum GPI-specific phospholipase D, albumin, active bacterial PI-specific phospholipase C or depletion of total GPI-APs from the culture medium. Serum inhibition of both transfer and glycogen/lipid synthesis was counteracted by synthetic phosphoinositolglycans (PIGs), which closely resemble the structure of the GPI glycan core and caused dissociation of GPI-APs from serum proteins. Finally, large, heavily lipid-loaded donor and small, slightly lipid-loaded acceptor adipocytes were most effective in stimulating transfer and lipid synthesis. In conclusion, full-length GPI-APs can be transferred between adipocytes or between blood cells as well as between these cell types. Transfer and the resulting stimulation of lipid and glycogen synthesis, respectively, are downregulated by serum proteins and upregulated by PIGs. These findings argue for the (patho)physiological relevance of the intercellular transfer of GPI-APs in general and its role in the paracrine vs. endocrine (dys)regulation of metabolism, in particular. Moreover, they raise the possibility of the use of full-length GPI-APs as therapeutics for metabolic diseases.

## 1. Introduction

Proteins are anchored at the plasma membranes (PM) of eukaryotic cells by several distinct modes, among them the penetration of the phospholipid bilayer by one or several proteinaceous transmembrane domains (mono- or polytopic transmembrane proteins), the non-covalent association with the extracellular domain of transmembrane proteins (peripheral membrane proteins) or the insertion of fatty acyl chains covalently linked to certain (amino- or carboxy-terminal) amino acids into the outer leaflet of the phospholipid bilayer (e.g., myristoylated, palmitoylated or farnesylated proteins). 

Moreover, certain eukaryotic membrane proteins are modified with a specific glycolipid, i.e., glycosylphosphatidylinositol (GPI), which becomes embedded in the outer phospholipid leaflet of PM and thereby anchors the protein moiety of these so-called GPI-anchored proteins (GPI-APs) at the cell surface (for the structure of GPI-APs, see Supplementary Materials Figure S1). In mammalian cells, GPI-APs account for about 1–2% of the translated proteins according to the carboxy-terminal consensus sequence for GPI anchor addition and encompass receptors, enzymes and adhesion molecules [1,2,3,4].

The overall structure of the GPI anchor is identical from yeast to man and constituted by amphiphilic phosphatidylinositol (PI) as the phospholipid component and a highly conserved hydrophilic glycan core (see for the structure of the glycan core, Supplementary Materials Figure S1, inset) [5,6]. This consists of a non-acetylated glucosamine and three mannose residues connected via specific glycosidic linkages. One end is glycosidically linked to the 6-hydroxyl group of PI, and the other non-reducing end forms a phosphodiester bridge to an ethanolamine residue. 

This terminal amino group of the GPI anchor is invariably amide-linked to the carboxy-terminus of the GPI-AP protein moiety. In mammalian cells, the mannose residues of the glycan core may be modified by additional phosphoethanolamine side branches, *N*-acetylgalactosamine, galactose, sialic acid and phospho-*N*-acetylglucosamine moieties [7,8,9,10,11,12]. The phospholipid component is typically built from a PI moiety with two long-chain (mostly saturated) fatty acyl chains, as either diacyl-PI or 1-alkyl-2-acyl-PI [13,14].

A variety of physiological functions have been attributed to the expression of GPI-APs at PM of eukaryotic cells. These include the mediation of cell adhesion and cell–cell interactions, enabled by the increased lateral mobility and denser packing at the outer leaflet of PM of GPI-APs compared to transmembrane and peripheral membrane proteins as well as the transmission of signals across PM through the interaction of the fatty acyl chains of the GPI anchor with those of acylated tyrosine kinases anchored at the inner leaflet of the PM [15,16]. Furthermore, the intercellular transmission of biological signals may be mediated by protein moieties of GPI-APs upon proteolytic or lipolytic removal of their GPI anchor by specific proteases or (G)PI-specific phospholipases C/D (PLC/D), such as GPLD1 and their resulting release into the blood stream or interstitial spaces, where they act as hormones or receptor (ant)agonists at cells distant from the releasing cells [17,18,19].

During the past two decades, another putative function unique for GPI-APs rather than transmembrane proteins has become the object of considerable research efforts. Those focus on the release of GPI-APs from PM, which does not involve proteolytic or lipolytic cleavage of the GPI anchor but instead engages shedding or exocytosis of extracellular vesicles or a yet unidentified molecular mechanism for their spontaneous or controlled release in non-vesicular structures [20]. 

With regard to the latter, so-called full-length GPI-APs with the complete GPI anchor remaining attached have recently been found to be released from eukaryotic cells in response to endogenous or environmental cues, such as age, size, lipid loading and metabolic state [21,22,23], and have meanwhile been recovered from extracellular aqueous compartments, such as body fluids or culture medium, in macromolecular structures, such as lipoprotein-like particles, micelle-like complexes and homo- or heteromultimers [24,25,26,27,28,29,30,31,32,33]. 

However, the (patho)physiological consequences of the release of full-length GPI-APs from donor cells and tissues into interstitial spaces or body fluids have escaped elucidation thus far. From a theoretical point of view, the following possibilities are conceivable: (i) removal of inactive, superfluous or deleterious GPI-APs from PM; (ii) modulation of the enzymic, adhesive or binding characteristics of PM as far as determined by GPI-APs; (iii) biogenesis of macromolecular GPI-AP complexes (in concert with other constituents) exerting specific catalytic, binding or signaling function in interstitial spaces or body fluids; and (iv) transfer of GPI-APs from donor to acceptor cells resulting in functional alterations in the latter. While studies have become available supporting possibilities (i) [34,35,36,37,38], (ii) [39,40,41,42,43,44] and (iii) [21,45,46,47,48], the experimental evidence for option (iv) [49,50,51,52] has remained limited thus far.

The following study was aimed at closing this gap (iv) at the cellular level. This work is based on previous data, which were obtained with a cell-free microfluidic chip-based surface acoustic wave (SAW) sensing system for analysis of the transfer of GPI-APs between isolated PM [53]. In greater detail, the efficacy of transfer between isolated rat adipocyte and erythrocyte PM was found to be determined by the genotype and metabolic state of the rats, from which the donor PM had been derived, with considerable increases in obese and diabetic rats compared to healthy controls. 

In the present study, two different cell-based test systems coupled to SAW sensing were introduced for the analysis of the transfer of GPI-APs from donor to acceptor human or rat adipocytes and human K562 erythroleukemia (EL) cells in homologous (identical cell type) or heterologous (between different cells types) configurations in parallel to effects on the metabolism of the corresponding acceptor cells, which exhibit reduced or even no endogenous expression of GPI-APs at their PM: (i) incubation of full-length GPI-APs reconstituted into micelle-like complexes with acceptor cells and (ii) incubation of donor and acceptor cells in transwell co-culture. Both test systems are prone to manipulation of the incubation conditions, such as addition of serum factors, depletion of GPI-APs during the transfer process and use of different configurations of donor and acceptor cells, for characterization of the transfer process and its correlation to functional alterations in the acceptor cells.

For the elucidation of the amount of specific GPI-APs that are transferred from (see above (i)) micelle-like GPI-AP complexes or (see above (ii)) PM of the donor cells to PM of the acceptor cells in course of incubation of (i) standard cell cultures or (ii) transwell co-cultures, a chip-integrated microfluidic sensor system was used. It relies on the propagation of SAW along the chip surface, which is affected by the binding of any entities to the chip [53,54,55]. This may happen in the course of the specific detection of a protein of interest expressed at PM during immobilization of the latter onto the chip with the aid of ionic or covalent capturing procedures and subsequent injection of appropriate antibodies into the microfluidic chip channels. 

The resulting right-ward phase shift (decrease in frequency) of the SAW represents a measure for the loaded mass, i.e., the amount of a specific protein in the sample, irrespective of whether being solubilized and in a pure state or embedded in authentic membranous or vesicular entities. Thus, for the analysis of the transfer of GPI-APs to PM of acceptor cells, both the initial capture of the PM prepared from the acceptor cells by the TiO_2_ chip surface and the subsequent measurement of the amount of specific GPI-APs expressed at the acceptor PM before and after incubation of the (i) micelle-like GPI-AP complexes or (ii) donor cells with the acceptor cells were monitored in real-time as increases in phase shift. 

Taken together, the identification and quantitative evaluation of specific GPI-APs at the acceptor PM, as reflected in increases in phase shift upon injection of relevant antibodies (of single or multiple types, i.e., in sandwich), was used as a measure for the transfer of full-length GPI-APs in both test systems. The data obtained strongly suggest that intercellular transfer of full-length GPI-APs within adipocytes or EL cells or between adipocytes and EL cells or vice versa leads to the stimulation of lipid (adipocytes) and glycogen (EL cells) synthesis. 

Both transfer and synthesis stimulation are inhibited by binding proteins for GPI-APs, such as serum GPLD1 or albumin, and are relieved of this inhibition in the course of their dissociation from GPI-APs by phosphoinositolglycans (PIGs; for structure, see Supplementary Materials, Figure S2) [56]. Differential concerted action of those factors in the interstitial tissue spaces vs. the blood compartment may determine the operation vs. blockade of paracrine and endocrine transfer, respectively, of GPI-APs as well as the up- vs. downregulation of lipid and glycogen synthesis in the corresponding acceptor tissues.

## 2. Results

### 2.1. Different Modes for Depletion of GPI-APs from PM of Different Cell Types

For elucidation of the physiological consequences of the transfer of GPI-APs to acceptor cells, it was reasoned that their expression of endogenous GPI-APs should be as low as possible. A low or no “background” of pre-existing GPI-APs should increase the sensitivity of the acceptor cells towards functional effects exerted by transferred exogenous GPI-APs. Furthermore, a “gradient” or “disequilibrium” from high to low or no PM expression of GPI-APs at acceptor cells may guarantee their unidirectional net flux from donor cells. 

In contrast, similar expression would create an “equilibrium” between donor and acceptor cells and thereby foster mutual exchange of GPI-APs between them at identical rates. The resulting bidirectional transfer and zero net flux would mask the potential effects of upregulation of the number of functional GPI-APs at PM compared to the control. Therefore, three different modes for the downregulation of GPI-AP expression at PM were used in the following to unravel functional effects of GPI-AP transfer to acceptor cells, two using cultured and one primary cells.

As first mode, cultured GPI anchor-defective EL cells incapable of the earliest step in the glycosylation of PI with acetylated glucosamine (GlcNAc), i.e., the formation of PI-*N*-GlcNAc, were selected as acceptor cells. Those clones had previously been identified during random in vitro mutagenesis for elucidation of the molecular defects in the GPI anchorage pathway, which are responsible for the phenotype of paroxysmal nocturnal hemoglobinuria [57,58]. 

In contrast to wildtype EL clones, which synthesize the highly conserved GPI glycan core of PI-GlcN-mannose_3_-phosphate-ethanolamine for anchorage of GPI-APs (see also Supplementary Materials, Figure S1), the GPI-deficient EL clones (“IA”) were previously shown to lack surface expression of the GPI-APs CD55 and CD59 [59]. This is compatible with the missing PI-*N*-acetyl-GlcN precursor for subsequent deacetylation, mannosylation and phosphoethanolamine transfer, which would yield the complete GPI anchor, competent for post-translational coupling of the corresponding protein moiety by a transferase [3,9,10,11]. As a consequence, in GPI-deficient EL clones (“IA”) the protein moieties of GPI-APs fail to be coupled to GPI anchors and transported to the PM but rather are degraded in the lumen of the endoplasmic reticulum [4,10,13].

**Figure 1:**
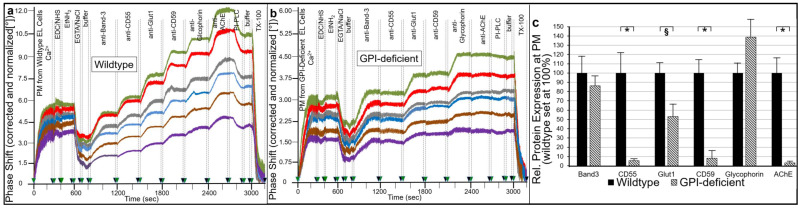
Inhibition of GPI-AP expression at PM of GPI-deficient EL cells. Wildtype and GPI-deficient EL cells were grown and used for preparation of PM. Following injection of 150 µL of acceptor PM from wildtype ((**a**,**c**) filled bars) or GPI-deficient ((**b**,**c**) hatched bars) EL cells (0.15–0.20 mg protein/mL) together with Ca^2+^ (ionic capture), 50 µL of EDC/NHS were injected into the chips followed in sequential order by 100 µL of 1 M EtNH_2_ (covalent capture), 50 µL of 1 mM EGTA/2 M NaCl and 50 µL of buffer each at a flow rate of 30 µL/min, 75 µL of antibodies against GPI-APs or transmembrane proteins as indicated, 75 µL of PI-PLC (*Bacillus cereus*, 7.5 mU/mL), 25 µL of washing buffer and 50 µL of TX-100 (0.1%) each at a flow rate of 15 µL/min. (**a**,**b**) PMs from six different cell clones were analyzed for phase shift by SAW sensing using different chips. Phase shift as a measure for expression of GPI-APs and transmembrane proteins at PM is given upon correction for unspecific interaction (no PM) and normalization for varying capturing efficacy of different chip channels (i.e., for identical amounts of PM captured). The experiment was repeated five times with similar results. Hatched vertical lines with green and black arrows indicate start and termination, respectively, of cycles of fluid injection at the time points indicated. (**c**) The values for the six different cell clones for wildtype and GPI-deficient EL cells each were corrected for identical phase shift induced by capture of the PM (at 815 s prior to antibody injection; GPI-deficient PM accounted for only 45–69% of the phase shift of wildtype PM) and then used for calculation of the phase shift Δ induced by each antibody (anti-Band-3, Δ 1200–800 s; anti-CD55, Δ 1500–1200 s; anti-Glut1, Δ 1800–1500 s; anti-CD59, Δ 2100–1800 s; anti-Glycophorin-A, Δ 2400–2100 s; anti-AChE 2700–2400 s). The mean values ± SD of the phase shift Δ were used for calculation of the relative protein expression at PM, with wildtype EL cells set at 100% each and significant differences vs. GPI-deficient ones indicated (* *p* ≤ 0.01, ^§^ *p* ≤ 0.05).

The different behavior of GPI-APs in GPI-deficient vs. wildtype EL clones was confirmed by analysis of their expression at PM using chip-based SAW sensing (see Introduction and for details of this analytical procedure Ref. [53]). For this, PMs from wildtype (Figure 1a) and GPI deficient EL cells (Figure 1b) were captured by negatively charged TiO_2_ chips in the presence of excess of Ca^2+^ through a combination of ionic (with negatively—and to a lower extent, positively—charged phospholipids) and hydrophobic (with zwitterionic phospholipids) interactions, yielding an almost complete coverage of the chip surface at high density (Figure 1, 0–300 s). 

This considerably increased the efficacy of the subsequent covalent capture of the PM by crosslinking to the activated TiO_2_ surface via the protein moieties of their constituent GPI-APs and transmembrane proteins using conventional EDC/NHS-based coupling chemistry (Figure 1, 300–400 s). Following blockade of the reaction by injection of ethanolamine (EtNH_2_, 400–600 s), the subsequent removal of Ca^2+^ by EGTA and final injection of NaCl to avoid unspecific binding of antibodies and then of buffer, the chip channels were stably covered with PM, presumably as enlarged and flattened vesicles (due to fusion as consequence of Ca^2+^-induced elimination of repulsive forces) and can then be used for assaying the expression of GPI-APs and transmembrane proteins.

The efficacies of ionic/covalent capture of PM derived from six different cell clones for wildtype and GPI-deficient EL cells each were monitored by right-ward phase shift (i.e., decrease in frequency) of the horizontal SAW propagating along the plane of the chip surface as measure for the loaded PM (Figure 1a,b).

For all PM preparations, 30% to 55% of the ionically captured PM (at 300 s) resisted injection of NaCl/EGTA and buffer (at 800 s) indicating covalent capture of a considerable portion of the PM. The ionic/covalent capturing efficacies (identical amounts of PM protein injected) differed between the six wildtype or GPI-deficient EL cell clones by 2.5- to 3-fold and between wildtype and GPI-deficient EL cell clones by 2- to 2.5-fold. 

The presence of the typical transmembrane proteins, Band-3, Glycophorin-A and glucose transporter-1 (Glut1), and the GPI-APs, CD55, CD59 and acetylcholinesterase (AChE), at the captured PM was determined by sequential injection (at 800 to 2700 s) of the corresponding antibodies and their binding in sandwich to the chip. This procedure led to stepwise increases in phase shift in response to anti-Band-3, Glut1 and Glycophorin-A antibodies as well as anti-CD55, CD59 and AChE antibodies for each clone of wildtype EL cells (Figure 1a). In contrast, GPI-deficient EL cells exhibited anti-Band-3, Glut1 and Glycophorin-A antibody-induced phase shift increases, only.

The antibody-induced phase shifts in sandwich reflected the expression of GPI-APs at PM of wildtype vs. GPI-deficient EL cells, which was confirmed by subsequent injection of bacterial PI-PLC (Figure 1a,b; at 2700–2900 s). This specifically removed the diacylglycerol moieties from the GPI anchors and thereby caused the loss of the GPI-AP protein moieties as well as of those PM vesicles captured through their GPI-APs from the chip leading to considerable reduction in phase shift with wildtype (Figure 1a) but not GPI-deficient (Figure 1b) EL cells. The apparently incomplete lipolytic digestion may be explained with impaired accessibility of the GPI-APs for PI-PLC from *Bacillus cereus*, which is known to depend on the type and species of the GPI-AP expressing cell. 

Importantly, acylation at the 2-position of the *myo*-inositol residue of the glycan core of human GPI-APs, such as erythrocyte AChE, was reported to impair cleavage by bacterial PI-PLC [60,61,62]. Thus, the unexpected finding that GPI-APs of EL cells and human adipocytes represented substrates for digestion by bacterial PI-PLC, at least to certain degree, could hint to partial regain of deacetylase expression during cell culture leading to limited amounts of non-acetylated GPI-APs. The residual PI-PLC-resistant portion of the phase shift increases was completely eliminated upon injection of TX-100 (Figure 1a,b; at 3000–3200 s), compatible with disintegration of the PM as control for the dependence of any phase shift increase on membranes captured by the chips.

Quantitative comparison of the six cell clones each (after correction for different capturing efficacies and normalization, see figure legend) revealed drastically diminished expression of CD55, CD59 and AChE in PM of GPI-deficient vs. wildtype EL cells (Figure 1c). In contrast, transmembrane proteins were not affected, with exception of a moderate reduction in Glut1 in GPI-deficient EL cells. These data confirmed the general and complete defect in PM expression of GPI-APs in EL cell clones “IA” as had been previously generated and characterized [63] and demonstrated the value of chip-based SAW sensing for the detection of proteins at PM, even at a very low level.

As second mode, downregulation of PM expression of GPI-APs was provoked in cultured human adipocytes with an inhibitor of GPI synthesis. Previously, the amino sugar mannosamine (ManN) was demonstrated to considerably reduce the incorporation of GPI into GPI-APs, possibly by leading to the depletion of transfer-competent GPI anchor precursor [64]. In polarized MDCK cells, ManN caused the conversion of both endogenous and recombinant GPI-APs, which are typically expressed at apical PM, to soluble proteins, which become secreted in an unpolarized fashion. 

Subsequent studies confirmed the capacity of ManN to inhibit the synthesis of full-length GPI-APs in other cell types [65,66,67]. Furthermore, they showed that ManN becomes incorporated into the glycan core at the 2-position from a mannose donor. It thereby prevents other glycan core components, such as mannose, from being coupled to this position, since the required hydroxyl group is replaced by a primary amino group [65]. Alternatively, ManN may operate as enzymatic inhibitor of α-1,2-specific mannosyl transferases [68].

**Figure 2:**
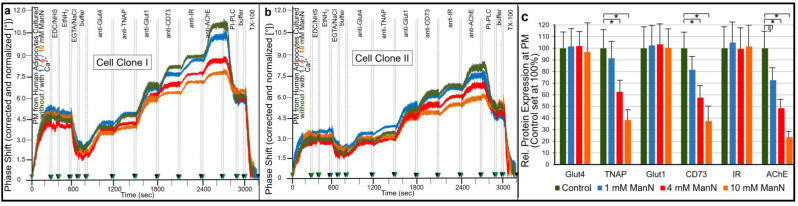
Lowering of GPI-AP expression at PM of human adipocytes by ManN. Human adipocytes of lipid-loading stage II (see Supplementary Materials, Methods and Figure S6) were incubated in the absence (green, control) or presence of increasing concentrations of ManN (blue, 1 mM; red, 4 mM; and orange, 10 mM) as described in the Materials and Methods. The expression of transmembrane proteins and GPI-APs at PM prepared from the adipocytes was analyzed by SAW sensing. (**a**,**b**) PM from two different cell clones (I,II) were analyzed using different chips. The measured phase shift is given upon correction and normalization as described in the legend to Figure 1. (**c**) The values from six independent cell clones for both control and ManN-treatment were used for calculation of the phase shift Δ induced by each antibody (anti-Glut4, Δ 1200–800 s; anti-TNAP, Δ 1500–1200 s; anti-Glut1, Δ 1800–1500 s; anti-CD73, Δ 2100–1800 s; anti-IR, Δ 2400–2100 s; anti-AChE, Δ 2700–2400 s). The mean values ± SD of the phase shift Δ were used for calculation of the relative protein expression at PM with control adipocytes set at 100% each and significant differences vs. ManN-treated ones indicated (* *p* ≤ 0.01, ^§^ *p* ≤ 0.05).

For demonstration of the effect of ManN on the expression of GPI-APs at PM in human adipocytes differentiated from human adipose-derived stem cells (hADSCs) in vitro [69,70], the same experimental design was used (Figure 2) as for the analysis of GPI-deficient EL cells (see Figure 1). Following the ionic and covalent capture to chips of PM prepared from human adipocytes that had been incubated with increasing concentrations of ManN, antibodies against typical adipocyte transmembrane proteins and GPI-APs were injected into the chips to assess their PM expression as the antibody-induced phase shift increases (Figure 2). 

As depicted for two different cell clones I and II (Figure 2a,b), ManN reduced the PM expression of the GPI-APs, tissue non-specific alkaline phosphatase (TNAP), CD73 and AChE, in a concentration-dependent fashion without significantly affecting that of the transmembrane proteins, glucose transporter-4 (Glut4), Glut1 and insulin receptor (IR). Quantitative evaluation of six independent cell clones revealed the downregulation of GPI-APs by 65% to 75% at 10 mM ManN, which was used in the following experiments and an apparent IC_50_ of 4 mM (Figure 2c).

As third mode for the downregulation of PM expression of GPI-APs, primary rat adipocytes were treated with methyl-ß-cyclodextrin (mßCD) for extraction of GPI-APs. GPI-APs are concentrated in lipid rafts, i.e., in nanodomains that are thought to be formed by self-aggregation of cholesterol and sphingolipids and to exist in a liquid-ordered state [71,72,73,74,75]. Consequently, cholesterol is an absolute requirement for the integrity of lipid rafts and its depletion leads to lipid raft dispersion with accompanying release of GPI-APs from intact viable cells, as was already demonstrated for rat adipocytes upon exposure towards cholesterol-chelating mßCD [71,76]. mßCD has a central cavity able to form a 2:1-complex with cholesterol [77,78] and (at variance with other agents causing acute cholesterol depletion, such as digitonin, filipin and saponin [78]) acts at the membrane surface, exclusively.

**Figure 3:**
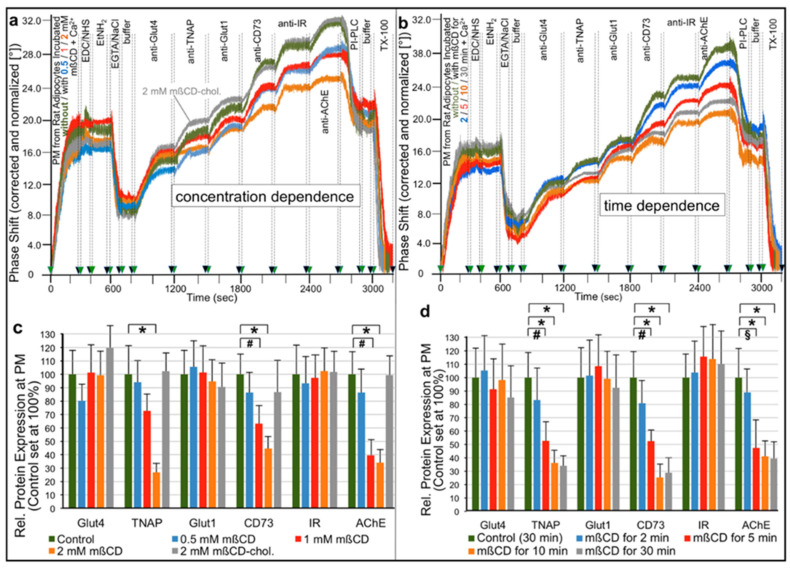
Removal of GPI-APs from PM of rat adipocytes by mßCD. Primary rat adipocytes were incubated (**a**,**c**) in the absence (green) or presence of increasing concentration of mßCD (blue, 0.5 mM; red, 1 mM; and orange, 2 mM) or 2 mM mßCD in complex with cholesterol (grey, mßCD-chol.) for 10 min or (**b**,**d**) in the absence (green) or presence of 10 mM mßCD for increasing periods of time (blue, 2 min; red, 5 min; orange, 10 min; and grey, 30 min) as described in Materials and Methods. The expression of transmembrane proteins and GPI-APs at PM, prepared from the adipocytes, was analyzed by SAW sensing. (**a**,**b**) PM from representative adipocyte preparations analyzed by different chips are shown and were repeated three to five times with similar results. The measured phase shift is given upon correction as described in the legend to Figure 1. (**c**,**d**) The values from four to six independent adipocyte preparations for both control and mßCD treatment were used for calculation and normalization of the phase shift Δ induced by each antibody. The mean values ± SD of the phase shift Δ were used for calculation of the relative protein expression at PM with control adipocytes set at 100% each and significant differences vs. mßCD-treated ones indicated (* *p* ≤ 0.01, ^#^ *p* ≤ 0.02, ^§^ *p* ≤ 0.05).

For the confirmation of extraction of GPI-APs from (lipid rafts of) PM of rat adipocytes as a result of mßCD-treatment, the same experimental design was used (Figure 3) as for the analysis of ManN-treated human adipocytes (see Figure 2). Following ionic and covalent capture by chips of PM prepared from rat adipocytes that had been incubated with increasing concentrations of mßCD for a constant period (Figure 3a) or with a constant concentration of mßCD for increasing periods (Figure 3b), antibodies against typical adipocyte transmembrane proteins and GPI-APs were injected into the chips to assess their PM expression as the antibody-induced phase shift increases (Figure 3). 

As depicted in representative experiments (Figure 3a,b), mßCD reduced the PM expression of the GPI-APs, TNAP, CD73 and AChE, in concentration- (Figure 3a) and time- (Figure 3b) dependent fashion without significantly affecting that of the transmembrane proteins, Glut4, Glut1 and IR. mßCD in complex with cholesterol failed to extract GPI-APs from PM demonstrating that this effect is specifically due to cholesterol depletion (Figure 3a, grey curves). 

This is in agreement with the negative correlation between the mßCD concentration applied and the cholesterol content left in rat adipocyte PM (see Materials and Methods, Section 4.6). Quantitative evaluation (Figure 3c,d) revealed maximal downregulation of GPI-APs by 55 to 75% (depending on the type of GPI-AP) at 2 mM mßCD and 10 min incubation. A longer extraction with 2 mM mßCD did not further increase extraction efficacy but caused considerable impairment of cell viability (see Materials and Methods, Section 4.6). Consequently, these conditions were used for subsequent analysis of the functional effects of the transfer of GPI-APs to primary rat adipocytes.

### 2.2. GPI-APs Are Transferred from Micelle-like GPI-AP Complexes to EL Cells or Adipocytes with Accompanying Stimulation of Glycogen and Lipid Synthesis, Respectively

For induction of transfer of exogenous GPI-APs to acceptor cells depleted of endogenous GPI-APs, GPI-deficient EL cells, ManN-treated rat adipocytes and mßCD-treated human adipocytes were incubated with micelle-like GPI-AP complexes, which were reconstituted from (lyso)phospholipids and total rat adipocyte GPI-APs (see Materials and Methods). 

**Figure 4:**
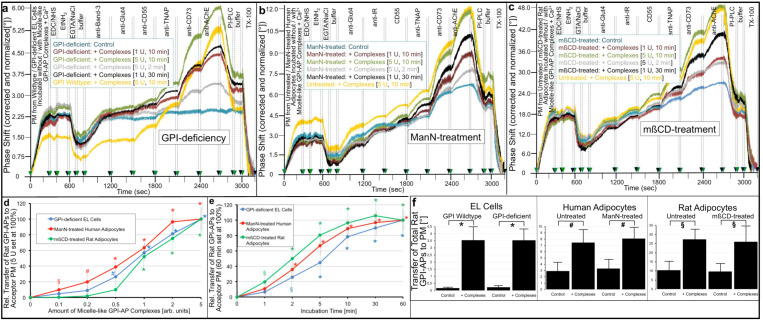
Transfer of full-length GPI-APs from micelle-like GPI-AP complexes to acceptor cells with reduced expression of GPI-APs. (**a**) Wildtype and GPI-deficient EL cells, (**b**) untreated and ManN-treated human adipocytes of lipid-loading stage II and (**c**) untreated and mßCD-treated rat adipocytes were incubated with buffer (Control) or the indicated amounts of micelle-like rat adipocyte GPI-AP complexes (prepared as described in Materials and Methods) for the indicated periods. The expression of rat adipocyte transmembrane proteins and GPI-APs at PM prepared from the cells was analyzed by SAW sensing as described in the legend to Figure 1. (**a**–**c**) PM from representative cell clones/preparations analyzed by different chips are shown, repeated two times with similar results. The measured phase shift is given upon correction and normalization as described for Figure 1. (**d**,**e**) GPI-deficient EL cells, ManN-treated human adipocytes and mßCD-treated rat adipocytes were incubated with increasing amounts (**d**) or two arb. units (**e**) of micelle-like GPI-AP complexes for 30 min (**d**) or increasing periods of time (**e**). (**d**,**e**) The mean values ± SD of the phase shift Δ from three to four independent complex/cell preparations for both wildtype/control and GPI-deficiency/treatment induced by all antibodies against rat GPI-APs (summation signal between 1500 and 2700 s each) were used for calculation of relative transfer of rat GPI-APs to acceptor cells with maximal incubations (5 arb. units, 60 min) set at 100% each and significant differences vs. incubation with buffer for 60 min (**d**) or complexes (5 arb. units) for 0 min (**e**) indicated. (**f**) The mean values ± SD of the phase shift Δ were used for calculation of transfer of total GPI-APs to acceptor PM for each cell type and incubation (5 arb. units of complexes, 10 min; yellow curves in **a**–**c**) with significant differences vs. control (buffer, 10 min; blue curves in **a**–**c**) indicated for each cell type (* *p* ≤ 0.01, ^#^ *p* ≤ 0.02, ^§^ *p* ≤ 0.05).

PMs prepared from these cells were monitored for the expression of rat adipocyte GPI-APs by chip-based SAW sensing (Figure 4). Upon covalent and ionic capture of the PM by the chip surface and subsequent injection of antibodies into the chip channels, the phase shift increases served as measure for expression of endogenous Band-3, Glut4 and IR (Figure 4a–c). As expected, their expression levels did not differ between cells incubated in the absence (Control, blue curves) and presence of micelle-like GPI-AP complexes. At variance, incubation with complexes led to considerable increases in expression of each of the rat adipocyte GPI-APs, CD55, TNAP, CD73 and AChE, compared to the control in all three cell types. This was dependent on the amount of complexes (Figure 4d) and incubation time (Figure 4e).

The elevation in complex-induced PM expression was more pronounced for EL cells (Figure 4a) compared to human (Figure 4b) and rat (Figure 4c) adipocytes. This is a consequence of the complete failure of expression of endogenous GPI-APs in GPI-deficient EL cells (Figure 4a, blue curve; Figure 4f, Control) compared to the only diminished one in adipocytes in combination with cross-reactivity of the antibodies used for human and rat adipocyte GPI-APs (Figure 4b,c, blue curves; Figure 4f, Control). 

Apparently, for all three cell types, the transfer of GPI-APs from micelle-like complexes did not differ significantly between normal (wildtype, untreated) and reduced (GPI-deficient, ManN-/mßCD-treated) endogenous expression of GPI-APs (Figure 4a–c, yellow and green curves; Figure 4f for quantitative evaluation). The injection of bacterial PI-PLC led to almost complete loss of the transferred rat adipocyte GPI-APs in all cell types (compared to absence of complexes, control, blue curves). 

This argued for anchorage of the transferred proteins by (non-acylated) GPI and thus for transfer of full-length GPI-APs. The complete abrogation of phase shift increases upon injection of TX-100 (Figure 4a–c) was compatible with transfer of the GPI-APs to PM of the acceptor cells. Taken together, the data demonstrated that EL cells or human/rat adipocytes with impaired expression of endogenous GPI-APs at PM can be used for studying functional effects of the transferred GPI-APs.

Some decades ago, PIGs were synthesized or prepared from isolated GPI-APs, which resemble in structure the glycan core of authentic GPI-APs to variable degree (see Supplementary Materials, Figure S2) and reported to exert potent insulin-mimetic effects, such as the stimulation of glucose transport and incorporation of glucose into lipids and glycogen, in insulin target cells, such as adipose and muscle cells [56,79,80,81]. However, these data as well as the proposed underlying molecular mechanisms have remained a matter of intense dispute (for details, see Discussion). The insulin-mimetic activity of a specific subset of structurally different PIGs synthesized by chemical means was re-evaluated in this study (Supplementary Materials, Figure S3). The findings can be summarized as follows:

(i) PIGs stimulated lipid synthesis in primary rat adipocytes dependent on structure and concentration to up to the maximal insulin effect (PIG41 at 20 µM; Supplementary Materials, Figure S3a,b). 

(ii) The experimental requirements for demonstration of maximal insulin-mimetic activity of PIGs differed considerably from those for maximal insulin action with regard to adipocyte size (large vs. small), incubation (high vs. low titer and intense vs. mild shaking) and preparation (long vs. short collagenase digestion) (Supplementary Materials, Figure S3a–f). This suggested different molecular mechanisms, i.e., extracellular pathways for PIGs involving remodeling of the PM in course of cell-size increase, cell-to-cell contact, mechanical stress and enzymatic attack vs. canonical intracellular insulin signaling. 

(iii) BSA or serum proteins at certain concentrations in the incubation medium of adipocytes were required to demonstrate the insulin-mimetic activity of PIGs, which was predominantly based on keeping the control activity low rather than by increasing the maximal PIG-induced activity (Supplementary Materials, Figure S4a). This was further enhanced by removal of divalent cations (Supplementary Materials, Figure S4b, for fold-stimulation in the presence of the Ca^2+^-chelating agent ortho-phenanthroline [Pha]). Together this argued for a critical role of serum proteins in mediation of the insulin-mimetic activity of PIGs. 

(iv) PIGs caused the dissociation of serum proteins from binding to full-length GPI-APs (Supplementary Materials, Figure S5a–c). This was presumably due to their structural similarity to the GPI glycan core (see Supplementary Materials, Figures S1 and S2), with PIG41 being most efficient as revealed by the IC_30_ of about 1 µM (Supplementary Materials, Figure S5d–f, black horizontal and vertical lines). 

Strikingly, relative upregulation of lipid synthesis exerted by the structurally different PIG41, 37, 45, 7 and 1 (in that ranking order of declining potency) was not affected by pretreatment, size or incubation of the adipocytes (Supplementary Materials, Figure S3) with BSA, total serum proteins or divalent cations (Supplementary Materials, Figure S4). The finding that PIGs inhibit the binding of GPI-APs to serum proteins suggested a causal relationship between the insulin-mimetic activity of PIGs and the release of full-length GPI-APs from serum proteins, possibly for their subsequent transfer to acceptor cells.

**Figure 5:**
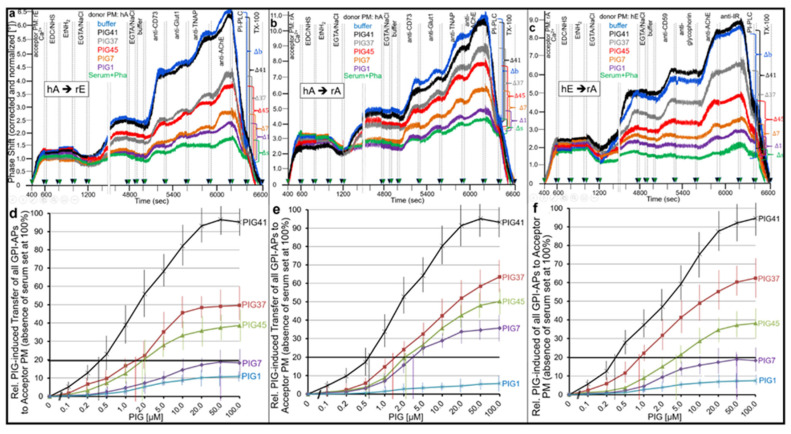
Effect of serum and PIGs on the cell-free transfer of full-length GPI-APs from donor to acceptor PM. Human adipocytes of lipid-loading stage IV (see Supplementary Materials, Figure S6) (**a**,**b**) or erythrocyte (**c**) donor PM (400 µL) were injected at 1200 s and at a flow rate of 60 µL/min into chips that had been coated with rat erythrocyte (**a**) and adipocyte (**b**,**c**) acceptor PM by ionic (Ca^2+^) and then covalent (EDC/NHS) capture for the donor–acceptor PM configurations as indicated. (**a**–**c**) After blockade with EtNH_2_ and washing with EGTA/NaCl, 100 µL of washing buffer (blue curves) or serum from obese ZDF rats (diluted 5-fold with buffer, green curves) together with 100 µM Pha alone or in combination with PIG41, 37, 45, 37 and 1 were injected. Thereafter, the chips were incubated until 4800 s at 37 °C at flow rate zero (double-hatched vertical lines). Following injection of EGTA/NaCl and then washing buffer, the expression of transmembrane proteins and GPI-APs at PM prepared from the cells was analyzed by SAW sensing as described in the legend to Figure 1. The experiments were repeated two times with similar results. Phase shift Δ elicited by donor PM and antibodies and eliminated by PI-PLC, as a measure for the transfer of all GPI-APs assayed, are indicated by brackets (Δb, buffer alone; Δs, serum together with Pha; Δ41, 37, 45, 7 and 1, PIG together with serum and Pha). The experiments were repeated three times with similar results. (**d**–**f**) 20 µL of serum together with increasing concentrations of PIGs were injected. The mean values ± SD of the phase shift Δ (Δ41, 37, 45, 7 and 1—Δs)/(Δb—Δs) were used for calculation of the relative PIG-induced transfer of GPI-APs to acceptor PM in the presence of serum with absence of serum set at 100% for each donor–acceptor PM configuration. The thick horizontal lines at 20% are used for delineation of the effective concentration for each PIG (EC_20_) as indicated by the vertical colored thin lines.

To substantiate causality between these processes, we tested whether the ranking order of PIG41, 37, 45, 7 and 1 for insulin-mimetic activity (Supplementary Materials, Figure S3) and dissociation of serum proteins from GPI-APs (Supplementary Materials, Figure S5) is reflected in their ability to induce transfer of full-length GPI-APs from donor to acceptor PM, prepared from human or rat adipocytes and rat erythrocytes in different configurations (Figure 5). For this, acceptor PMs were immobilized by sequential ionic (400–600 s) and covalent coupling (600–800 s). Upon termination of the coupling reactions (800–1200 s), donor PM with full-length GPI-APs embedded were injected together with buffer or serum alone or serum in combination with PIGs (1200–4800 s). Following removal of the donor PM by washing (4800–5000 s), the transfer of GPI-APs to acceptor PMs was monitored as described above (Figure 1).

As expected, transfer was detected for GPI-APs (CD73, TNAP, AChE and CD59) but not for transmembrane proteins (Glut1, Glycophorin-A, IR), dependent on the donor–acceptor PM configuration (Figure 5). GPI anchorage of the transferred GPI-APs was demonstrated by about 70% loss due to cleavage of their GPI anchor by bacterial PI-PLC (6200–6400 s). Cleavage-resistant GPI-APs were completely eliminated by TX-100 (6400–6600 s), compatible with transfer to PM captured by the chip surface. 

Importantly, with each of the three donor–acceptor PM configurations (Figure 5a–c), the presence of serum from obese Zucker diabetic fatty (ZDF) rats (together with Pha) during injection of the donor PM led to drastic reduction of GPI-AP transfer (Figure 5a–c, green curves) compared to buffer (Figure 5a–c, blue curves). This serum-induced blockade was abrogated by the simultaneous presence of PIGs (at 20 µM), with PIG41 being the most efficient and almost reaching the complete restoration of transfer, followed by PIG37, 45 and finally PIG 7. PIG1 exerted almost no relief of inhibition. 

This ranking order was confirmed by measurement of the concentration dependence of PIG-induced GPI-AP transfer in the presence of serum (together with Pha) for each of the donor–acceptor PM configurations (Figure 5d–f) with effective concentrations for 20% (EC_20_) of maximal transfer (i.e., measured for the absence of serum) of 0.34–0.55 µM for PIG41, 0.87–1.59 µM for PIG37, 2.0–4.3 µM for PIG45, 3.1–50 µM for PIG7 and >100 µM for PIG1. 

These cell-free data suggested that rat serum contains components that interfere with intercellular transfer of GPI-APs through interaction with the glycan core of the GPI anchor. One candidate component represents GPLD1, which according to previous findings, is amenable to the inhibition of cleavage by the removal of Ca^2+^ (here provoked by Pha), thereby, stabilizing its interaction with GPI anchors [53].

**Figure 6:**
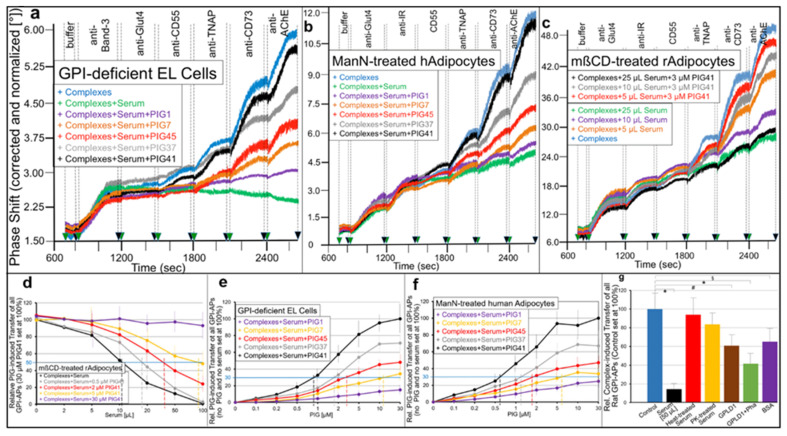
Effect of serum and PIGs on the transfer of full-length GPI-APs from micelle-like GPI-AP complexes to acceptor cells. (**a**) GPI-deficient EL cells, (**b**) ManN-treated human adipocytes of lipid-loading stage II or (**c**) mßCD-treated rat adipocytes were incubated (10 min, 37 °C) with micelle-like rat adipocyte GPI-AP complexes (5 arb. units) in the absence or presence of serum (30 µL) and PIGs (30 µM) (**a**,**b**) or as indicated (**c**). The expression of GPI-APs and transmembrane proteins at PM prepared from the cells was analyzed by SAW sensing as described in the legend to Figure 1. (**d**–**f**) mßCD-treated rat adipocytes (**d**), GPI-deficient EL cells (**e**) and ManN-treated human adipocytes (**f**) were incubated (60 min, 37 °C) with complexes (5 arb. units), serum (30 µL) and increasing concentrations of PIGs (**e**,**f**) or in the absence (black curve) or presence of PIG41 (colored curves) and serum at the indicated concentrations and volumes, respectively (**d**). The mean values ± SD of the phase shift Δ from four to six independent cell clones/preparations and incubations induced by all antibodies against rat GPI-APs (summation signal between 1500 and 2700 s) were used for calculation of the relative PIG-induced transfer of all rat GPI-APs to acceptor cells with 30 µM PIG41 in the presence of serum (**e**,**f**) or absence of both PIG41 and serum (**d**) set at 100%. The blue horizontal lines and the colored hatched vertical lines indicate (**e**,**f**) the concentration for 30% induction of transfer in the presence of serum for each PIG (EC_30_) and (**d**) the serum volume for 50% inhibition of transfer in the absence or presence of PIG41 (IV_50_). (**g**) mßCD-treated rat adipocytes were incubated (60 min, 37 °C) with complexes (5 arb. units) in the absence (Control, blue bar) or presence of untreated (black bar), heat-treated (red bar) or proteinase K-treated (PK, yellow bar) serum (50 µL) or GPLD1 (0.5 U) in the absence (brown bar) or presence of Pha (2 mM, green bar) or BSA (50 mg/mL, pink bar). The mean values ± SD of the phase shift Δ from three to four independent complex/cell preparations induced by all antibodies against rat GPI-APs (summation signal between 1500 and 2700 s) were used for calculation of the relative complex-induced transfer of all rat GPI-APs to acceptor cells with control set at 100% and significant differences vs. control indicated (* *p* ≤ 0.01, ^#^ *p* ≤ 0.02, ^§^ *p* ≤ 0.05).

Next, it was studied whether inhibition by serum proteins and its relief by PIGs holds true for the transfer of GPI-APs from micelle-like complexes to acceptor cells (Figure 6). For this, GPI-deficient EL cells (Figure 6a,e), ManN-treated human adipocytes (Figure 6b,f) and mßCD-treated rat adipocytes (Figure 6c,d) were incubated with micelle-like complexes reconstituted with rat adipocyte GPI-APs in the presence of serum and PIGs of different structure and then analyzed for expression of rat adipocyte GPI-APs at the acceptor cell PM as described for Figure 1. 

The transfer of CD55, TNAP, CD73 and AChE from the complexes to each cell type (Figure 6a–c, blue curves) was completely inhibited by serum (Figure 6a–c, green curves; taking into consideration their (low) endogenous expression in adipocytes but not in EL cells). This inhibition, which was dependent on the volume of serum (Figure 6d, black curve), was abrogated by PIGs at variable potency dependent on their concentration (Figure 6e,f) and structure (Figure 6a–c,e,f). The ranking order (of declining potency) for PIG-induced transfer of each GPI-AP from complexes to each cell type in the presence of serum was PIG41 > PIG37 > PIG45 > PIG7 > PIG1 as revealed by their increasing EC_30_ (Figure 6e,f; vertical hatched lines) and thus matched those for transfer from isolated donor to acceptor PM (see Figure 5). 

Consequently, increasing concentrations of PIG41 reduced the potency of serum to inhibit transfer as reflected in right-ward shifts of the serum volume–inhibition curves (Figure 6d). This led to elevation of the volumes required for 50% inhibition of transfer (IV_50_, vertical hatched lines). At 30 µM PIG41 no effect of serum on transfer was observed (Figure 6f, pink curve). Together, the data strongly argued for competition of GPI-APs and PIGs for interaction with some serum components that interfere with transfer of GPI-APs from micelle-like complexes to acceptor cells.

Next, the nature of those serum components was characterized (Figure 6g). For this, rat serum was treated with heat (red bar) or PK (yellow bar) and then assayed for effect on complex-induced transfer of rat adipocyte GPI-APs to mßCD-treated rat adipocytes. Both heat- and PK-treated serum did not impair transfer, arguing for the proteinaceous nature of the transfer-inhibiting serum component. Furthermore, GPLD1 alone (Figure 6g, brown bar) and, more pronouncedly, in combination with Pha (green bar) interfered with complex-induced transfer of GPI-APs, which was compatible with the assumed function of GPLD1 as binding-protein for full-length GPI-APs (see Figure 5). Finally, BSA at a concentration in the range of serum concentration of albumin was identified as an inhibitor of transfer (Figure 6g, pink bar) with potency comparable to GPLD1.

Remarkably, the relative potency of PIGs in preventing serum inhibition of transfer of GPI-APs from isolated donor to acceptor PM (Figure 5) and from micelle-like complexes to acceptor cells (Figure 6) was identical with that for stimulation of lipid synthesis in rat adipocytes (Supplementary Materials, Figure S3) and glycogen synthesis in rat diaphragms [56]. This correlation suggested a role of intercellular transfer of GPI-APs in the regulation of lipid and glycogen synthesis in the acceptor cells.

**Figure 7:**
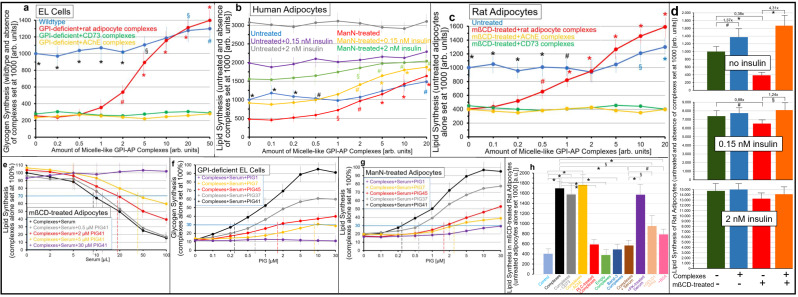
Stimulation of glycogen and lipid synthesis by micelle-like GPI-AP complexes in acceptor cells and effect of serum proteins and PIGs. (**a**–**d**) Wildtype and GPI-deficient EL cells (**a**), untreated and ManN-treated human adipocytes of lipid-loading stage II (**b**) or untreated and mßCD-treated rat adipocytes (**c**,**d**) were incubated (30 min, 37 °C) without or with micelle-like GPI-AP complexes (**a**–**c**, increasing amounts; **d**, 10 arb. units), reconstituted with total rat adipocyte GPI-APs (**a**–**d**), bAChE or rCD73 (**a**,**c**) as described in Materials and Methods, in the absence (**a**,**c**) or presence (**b**,**d**) of half-maximal (0.15 nM) and maximal (2 nM) effective concentrations of insulin and then assayed for glycogen (**a**) or lipid (**b**–**d**) synthesis as described in Materials and Methods. (**a**–**d**) The mean values ± SD (4–6 independent incubations and assays, each) of the synthesized glycogen and lipid are given as arb. units set at 1000 for incubation of wildtype or untreated cells without complexes as well as insulin. Significant increases vs. absence of complexes are indicated by colored symbols. Significant differences between wildtype and GPI-deficient EL cells or untreated and treated adipocytes are indicated by black symbols. (**d**) Lipid synthesis in mßCD-treated rat adipocytes is given for untreated adipocytes in the absence of complexes set at 1000 arb. units. (**e**–**g**) mßCD-treated rat adipocytes (**e**), GPI-deficient EL cells (**f**) and ManN-treated human adipocytes (**g**) were incubated (30 min, 37 °C) with micelle-like GPI-AP complexes from total rat adipocyte GPI-APs (20 arb. units) in the absence or presence of serum (**e**, increasing volumes; **f**,**g**, 50 µL) as well as increasing concentrations of PIGs and then assayed for lipid (**e**,**g**) or glycogen (**f**) synthesis. (**e**–**g**) The mean values ± SD (3–7 independent incubations and assays, each) of the synthesized glycogen and lipid are given with complexes alone set at 100%. The volumes of serum (**e**) or concentrations of PIGs (**f**,**g**) required for 30% decrease (horizontal blue line) in lipid synthesis (**e**; IV_30_) or 30% increase (horizontal blue lines) in glycogen (**f**; EC_30_) or lipid (**g**; EC_30_) synthesis are given by the hatched vertical lines for each PIG. (**h**) mßCD-treated rat adipocytes were incubated (30 min, 37 °C) in the absence (light blue bar) or presence of micelle-like GPI-AP complexes from total rat adipocyte GPI-APs (20 arb. units), which had been left untreated (black bar) or immune depleted from rCD73 (grey bar) or rAChE (yellow bar) or treated with bacterial PI-PLC (red bar) or with complexes that had been reconstituted without proteins (green bars) or with erythrocyte Band-3 (dark blue bar) or with complexes together with untreated serum (50 µL, brown bar) or PK-treated serum (pink bar) or GPLD1 (0.2 units/mL) together with Pha (2 mM, orange) or BSA (50 mg/mL, light red) and then assayed for lipid synthesis. The mean values ± SD (four or five independent incubations and assays, each) of the synthesized lipid are given as arb. units set at 1000 for incubation of untreated rat adipocytes without complexes. Significant differences are indicated (* *p* ≤ 0.01, ^#^ *p* ≤ 0.02 and ^§^ *p* ≤ 0.05).

To test for this possibility, EL cells and human or rat adipocytes with impaired PM expression of GPI-APs were incubated with micelle-like complexes reconstituted with total rat adipocyte GPI-APs or purified rCD73 or bAChE (in the absence of serum proteins as well as BSA) and then assayed for synthesis of glycogen and lipids, respectively (in the presence of BSA) (Figure 7). 

In GPI-deficient EL cells (Figure 7a) as well as ManN-treated human (Figure 7b) and mßCD-treated rat (Figure 7c) adipocytes, glycogen and lipid synthesis, respectively, was upregulated by rat adipocyte GPI-AP complexes (Figure 7a–c, red curves) in concentration-dependent fashion to up to 5.6-, 3.3- and 3.9-fold above basal, respectively. In contrast, in wildtype EL cells as well as untreated human and rat adipocytes only moderate stimulations of glycogen and lipid synthesis were detectable, even at high amounts of complexes (Figure 7a–c, blue curves). 

Importantly, rCD73 (Figure 7a–c, green curves) and bAChE (orange curves) complexes were inactive in each cell type. In insulin-responsive ManN-treated human (Figure 7b) and mßCD-treated rat (Figure 7d) adipocytes the simultaneous presence of rat adipocyte GPI-AP complexes and half-maximal (Figure 7b, orange curves; Figure 7d, orange bars) or maximal (Figure 7b, green curves; Figure 7d, orange bars) effective concentrations of insulin stimulated lipid synthesis in additive and sub-additive fashion, respectively, vs. complexes alone (Figure 7b, red curve) or insulin alone (Figure 7d, red bars). 

This resulted in declining fold-stimulations by complexes in the presence of insulin (Figure 7b) and vice versa by insulin in the presence of complexes (Figure 7d). Together, these data argued that transfer of GPI-APs from micelle-like complexes reconstituted with total rat adipocyte GPI-APs, rather than solely with rCD73 or bAChE, to EL cells and adipocytes with downregulated PM expression of GPI-APs leads to stimulation of glucose and lipid synthesis, respectively, involving a signaling pathway that differs from that of insulin.

As expected, based on the interference of serum proteins with the transfer of GPI-APs from micelle-like complexes to EL cells and adipocytes (see Figure 6), serum reduced complex-induced lipid synthesis in mßCD-treated rat adipocytes in volume-dependent fashion by up to 85% to 90% (Figure 7e, black curve). Serum inhibition was diminished by PIG41 in concentration-dependent fashion to up to complete abrogation at 30 µM (Figure 7e, pink curve). Increasing concentrations of PIG41 necessitated increasing volumes of serum for 30% inhibition of complex-induced synthesis in mßCD-treated rat adipocytes (Figure 7e, colored curves and vertical hatched lines, IV_30_), GPI-deficient EL cells and ManN-treated human adipocytes (data not shown). 

Structurally different PIGs considerably differed in their efficacy to counteract serum inhibition of complex-induced synthesis in GPI-deficient EL cells (Figure 7f), ManN-treated human adipocytes (Figure 7g) and mßCD-treated rat adipocytes (data not shown), with PIG41 being most potent, followed by PIG37, PIG45 and finally PIG7, as reflected in their increasing EC_30_ (Figure 7f,g, vertical hatched lines). PIG1 was almost inactive. The similar EC_30_ of PIGs for abrogation of serum inhibition of complex-induced transfer (Figure 6) and glycogen and lipid synthesis (Figure 7f,g) argued for causal relationship between transfer and stimulation of synthesis.

Next, the nature of the complexes and serum component that manage to induce lipid synthesis and interfere with it, respectively, was studied with mßCD-treated adipocytes (Figure 7h). Micelle-like complexes reconstituted with total rat adipocyte GPI-APs that had been immune depleted of rCD73 (grey bar) or rAChE (orange bar) did not differ from untreated complexes (black bars) in stimulating lipid synthesis. Complexes treated with bacterial PI-PLC failed to stimulate lipid synthesis (red bar). Complexes reconstituted without proteins (green bars) or erythrocyte Band-3 protein (dark blue bar) were inactive. 

Serum digested with PK failed to inhibit complex-induced lipid synthesis (Figure 7h, pink bar). Remarkably, GPLD1 in combination with Pha (light brown bar) or BSA at a concentration prevalent in serum (pink bar) significantly interfered with complex-induced lipid synthesis by 50% to 60%. Together these data, which were qualitatively reproduced for glycogen synthesis in GPI-deficient EL cells (data not shown), confirmed that the transfer of certain adipocyte GPI-APs, but not of CD73 and AChE, is prerequisite for complex-induced glycogen and lipid synthesis in acceptor cells. Furthermore, its inhibition by serum depends on a proteinaceous component, which apparently includes, but is not restricted to, GPLD1 and albumin.

A previous study demonstrated positive correlation of the release of GPI-APs from primary rat adipocytes to their size and, as a consequence, to the age of the donor animals [82,83]. Therefore, the impact of cell size on the stimulation of lipid synthesis by micelle-like rat adipocyte GPI-AP complexes was studied next (Supplementary Materials, Figure S6). For this, adipocytes were used that had been prepared from rats of three different age classes and thus corresponded to size classes I = small, II = medium and IV = large diameter) [82].

In agreement with literature data [84,85,86,87], basal lipid synthesis (absence of insulin) was the lowest for small adipocytes, followed by medium and large ones (Supplementary Materials, Figure S6a). Consequently, both insulin sensitivity (Figure S6a) and responsiveness (Supplementary Materials, Figure S6c) were the highest for small, followed by medium and then large adipocytes as reflected in the increasing apparent EC_50_ (0.19, 0.55 and 1.34 nM, horizontal and vertical hatched lines) and significantly decreasing fold-stimulations by insulin, respectively. 

Moreover, as manifested in the absolute increases (Supplementary Materials, Figure S6b) and fold-stimulations (Supplementary Materials, Figure S6d) of lipid synthesis, small rat adipocytes (Supplementary Materials, Figure S6b,d, blue curve and bar) exhibited the highest responsiveness towards micelle-like GPI-AP complexes, followed by medium (Supplementary Materials, Figure S6b,d, red curve and bar) and then large (Figure S6b,d, green curve and bar) cells. 

Again, stimulation of lipid synthesis by complexes (as shown here only for small adipocytes) turned out to rely on total rat adipocyte GPI-APs (Supplementary Materials, Figure S6b, blue and pink curves) rather than solely on CD73 (orange curve) or AChE (black curve) as revealed by adsorption of total rat GPI-APs to α-toxin Sepharose beads (Supplementary Materials, Figure S6b, pink curve) and immune depletion of rCD73 or rAChE (Supplementary Materials, Figure S6b, orange and black curves), respectively. Thus, upregulation of lipid synthesis in rat adipocytes in response to transfer of total adipocyte GPI-APs depends on the cell size/age of the donor animals with small size/low age being most effective.

### 2.3. GPI-APs Are Transferred between EL Cells and Human Adipocytes in Transwell Co-Culture with Accompanying Stimulation of Glycogen and Lipid Synthesis, Respectively

The above findings of transfer of rat adipocyte GPI-APs from micelle-like complexes to human EL cells or adipocytes with impaired expression of GPI-APs at PM (see Figure 4) under accompanying upregulation of glycogen and lipid synthesis, respectively (see Figure 7), prompted the question as to whether transfer of GPI-APs in parallel to synthesis stimulation occurs also in a more physiological setting between cells. 

To test for this, putative donor and acceptor cells were cultured in the insert wells at the top and in the companion bottom wells, respectively, of transwell co-cultures, which are separated from one another by a semipermeable filter plate (with pores of 1 µM diameter). This configuration prevented direct cell-to-cell contact and enabled passage of large molecules and complexes, such as lipoprotein-like complexes and micelle-like GPI-AP complexes (data not shown), but not of extracellular vesicles and cells (data not shown). 

**Figure 8:**
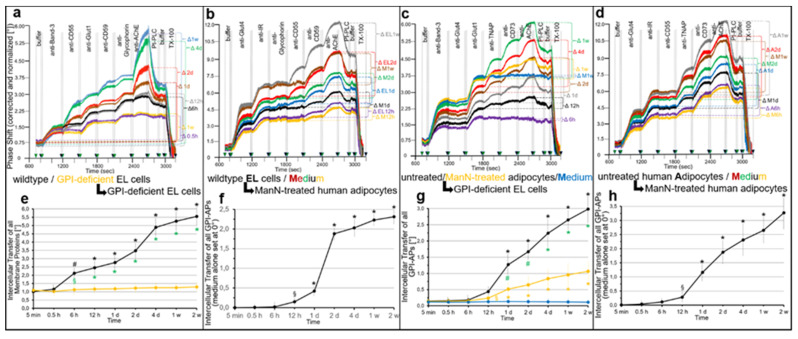
Transfer of full-length GPI-APs from donor to acceptor cells in transwell co-culture. Transwell co-cultures were run with donor and acceptor cells in the insert and bottom wells, respectively, as described in Materials and Methods in homologous configuration ((**a**,**e**), GPI-deficient [orange curves] or wildtype [other colors] EL cells and GPI-deficient EL cells; (**d**,**h**), untreated human adipocytes of lipid-loading stage II [A] or medium alone [M] and ManN-treated human adipocytes of lipid-loading stage II) or heterologous configuration ((**b**,**f**), wildtype EL cells [EL] or medium alone [M] and ManN-treated human adipocytes of lipid-loading stage II; (**c**,**g**), ManN-treated [orange curves] or untreated [other colors] human adipocytes of lipid-loading stage II or medium alone [blue curve]). After incubation (37 °C) for various times (h, hours; d, days; and w, weeks), PM were prepared from the cells of the bottom wells, coupled to chips by ionic/covalent capture and then analyzed for expression of the membrane proteins indicated by SAW sensing as described for Figure 1. (**a**–**d**) Phase shifts induced by binding of antibodies against GPI-APs or transmembrane proteins and by PI-PLC- and TX-100-treatment (from 700 to 3100 s) are shown, only, omitting the preceding capturing procedures (0–700 s, see Figure 1). Correction and normalization of the phase shift were performed as described in the legend to Figure 1. Phase shift Δ between injection of the first (at 800 s) and last (at 2700 s) antibodies against membrane proteins (**a**) as well as of the first (at 1800 s (**b**,**c**); 1500 s (**d**)) and last (2700 s (**b**–**d**)) antibodies against GPI-APs are indicated by horizontal hatched lines and brackets for each period ([A] donor adipocytes, [EL] donor EL cells, [M] medium alone in the inset wells). (**e**–**h**) The experiments were repeated three to six times for the indicated configurations and periods. Phase shift Δ induced by antibodies against all membrane proteins (**e**) or only against all GPI-APs (**f**–**h**) were used for calculation of the homologous (**e**,**h**) or heterologous (**f**,**g**) intercellular transfer. (**f**,**h**), Phase shift Δ for each incubation of acceptor with donor cells was corrected for that with medium alone in the insert wells (“M” in (**b**,**d**)). (**e**–**h**) Significant differences vs. 5-min incubation are indicated (black and orange symbols). Significant differences between wildtype and GPI-deficient EL cells (**e**) or untreated and ManN-treated human adipocytes (**g**) as donor cells are indicated (green symbols) (* *p* ≤ 0.01, ^#^ *p* ≤ 0.02, ^§^ *p* ≤ 0.05).

**Figure 9:**
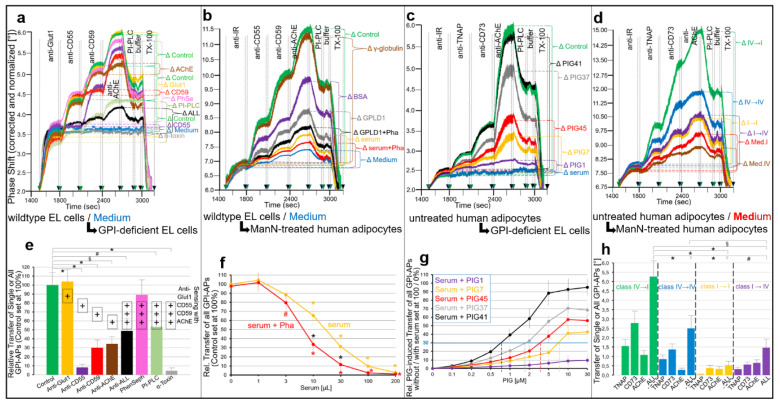
Biochemical and cell biological characterization of the intercellular transfer of GPI-APs. Transwell co-cultures were run with (**a**,**e**) wildtype and GPI-deficient EL cells, (**b**,**f**) wildtype EL cells and ManN-treated human adipocytes of lipid-loading stage II (see Supplementary Materials, Figure S6), (**c**,**g**) untreated human adipocytes of lipid-loading stage II and GPI-deficient EL cells, or (**d**,**h**) untreated and ManN-treated human adipocytes of the indicated stage of lipid-loading (class donor → class acceptor adipocytes) in the insert and bottom wells, respectively, as described in the legend to Figure 8. Where indicated, only medium was present in the insert wells (**a**,**b**, Δ Medium; **d**, Δ Med.I/IV for adipocytes of lipid-loading stage class I and IV, respectively). After incubation (37 °C, 1 week, serum- and albumin-free medium) in the absence (Δ Control) or presence (**a**,**e**) of single antibodies (source and titer used see Ref. [83] against Glut1, AChE, CD55 or CD59, or a combination of antibodies against all GPI-APs (ALL), each coupled to protein A Sepharose, or phenylsepharose (150 µL, PhSe/PhenSeph) or PI-PLC (*Bacillus cereus*, 0.2 U/mL) or α-toxin coupled to Sepharose (50 µL), or (**b**,**f**) γ-globulin (15 mg/mL), or BSA (50 mg/mL), or GPLD1 (100 mU/mL), or GPLD1 with Pha (2 mM), or rat (obese ZDF) serum (100 µL), or rat (obese ZDF) serum with Pha (2 mM) or (**c**,**g**) PIGs as indicated (10 µM) or rat (obese ZDF) serum (100 µL), PM were prepared from the cells of the bottom wells and then analyzed for the expression of GPI-APs and transmembrane proteins by chip-based SAW sensing as described for Figure 1. (**a**–**d**) Phase shifts induced by antibody binding and PI-PLC- or TX-100-treatment (from 1500 to 3100 s) after correction and normalization are shown, only, with those induced by all antibodies against GPI-APs (summation signals between 1800 and 2700 s) are indicated by horizontal hatched lines and brackets for each period. (**e**–**h**) The experiments were repeated three to five times for the indicated configurations and agents (**e**), serum volumes (**f**) and concentrations of PIGs (**g**). (**e**,**f**) Phase shift Δ for each incubation of acceptor with donor cells was corrected for that with medium alone in the insert wells (Δ Medium) and then used for calculation of the relative transfer of single or all GPI-APs as monitored by chip-based sensing with the indicated single or combined antibodies, respectively, with controls (no added agent or serum) set at 100%. Significant differences vs. control (**e**) or absence of serum (**f**, orange and red symbols) as well as for serum vs. serum + Pha (**f**, black symbols) are indicated. (**g**) Phase shift Δ were used for calculation of the relative PIG-induced transfer of all GPI-APs with absence and presence of serum set at 100 and 0%, respectively. (**h**) Phase shift Δ were corrected for those with medium alone in the insert wells (Δ Med.I/IV, Δ Medium for lipid-loading stage I or IV) for all configurations of stages of class donor → class acceptor adipocytes and used for calculation of transfer of single or all GPI-APs [°]. Significant differences between the transfer of all GPI-APs (ALL) are indicated (* *p* ≤ 0.01, ^#^ *p* ≤ 0.02, ^§^ *p* ≤ 0.05).

Upon incubation, intercellular transfer of GPI-APs, which encompasses their (i) release from donor cells in the insert wells, (ii) diffusion across the filter plate to the bottom wells and (iii) subsequent insertion into the PM of acceptor cells, may occur in parallel to stimulation of glycogen and lipid synthesis in the acceptor cells. Transfer to PM of the acceptor cells was monitored by chip-based SAW sensing as described for Figure 1 (Figure 8), and glycogen and lipid synthesis was assayed in the acceptor cells upon addition of [U-^14^C]glucose and NBD-FA, respectively, to the bottom wells (Figure 9).

The incubation of wildtype EL cells or untreated human adipocytes as donor cells with GPI-deficient EL cells or ManN-treated human adipocytes as acceptor cells in transwell co-culture in both homologous (Figure 8a,d) and heterologous configurations (Figure 8b,c) led to considerable increases in PM expression of the GPI-APs, CD55, CD59 and AChE (Figure 8a,b) and TNAP, CD73 and AChE (Figure 8c,d), respectively, during incubation for 0.5 h to 1 week (Figure 8e). In contrast, PM expression of transmembrane proteins did not increase. As expected, GPI-deficient EL cells (Figure 8a) or ManN-treated human adipocytes (Figure 8c) as donor cells in the insert wells did not support transfer during 1 week of culture (orange curves). 

Quantitative evaluation of the intercellular transfer of all GPI-APs between wildtype and GPI-deficient EL cells (Figure 8e) or untreated human adipocytes and GPI-deficient EL cells (Figure 8g) revealed significant differences between donor cells with normal (Figure 8e,g, black curves) and no/low (Figure 8e,g, yellow curves) PM expression of GPI-APs (Figure 8e,g, green symbols), compatible with complete defect in synthesis of GPI-APs as already demonstrated above (see Figure 1). As an additional control, incubation of GPI-deficient EL cells with culture medium alone (Figure 8c, 1 week, Δ M1w; Figure 8g, up to 2 weeks; blue curves) did not support GPI-AP transfer.

For correction of the residual endogenous expression of GPI-APs at PM in acceptor cells during co-culture with donor wildtype EL cells (Figure 8b, EL) and untreated human adipocytes (Figure 8d, A), ManN-treated human adipocytes were incubated with culture medium alone (M) in parallel. The total increases in GPI-AP expression at their PM were significantly higher with wildtype EL cells (Figure 8b) and untreated human adipocytes (Figure 8d) as donor cells in comparison to medium alone after co-culture for at least one day. Quantitative evaluation revealed significant increases in transfer between wildtype EL cells and ManN-treated human adipocytes (Figure 8f) as well as between untreated and ManN-treated human adipocytes (Figure 8h) after co-culture for at least 12 h.

Taken together, these data were compatible with (i) release of GPI-APs from donor cells in the insert wells, (ii) their subsequent diffusion across the filter plate into the bottom wells and (iii) their final insertion into the PM of acceptor cells expressing low or no GPI-APs. Thus, transwell co-culture enables monitoring of the transfer of GPI-APs between cells separated by an aqueous compartment.

On basis of determination of the absolute numbers of AChE molecules at the PM of donor and acceptor cells before and after incubation (for one week) in transwell co-culture using normalized chip-based SAW sensing with AChE purified from human erythrocytes as standard (see Supplementary Materials, legend to Table S1) and PM derived from defined numbers of cells grown in the inset and bottom wells as well as under consideration of the non-linear response of phase shift with mass (i.e., GPI-Aps, including AChE) loaded onto the chip (in particular in case of sandwich configuration of differing complexity), the percentage of GPI-APs transferred under the experimental conditions of Figure 8 was found to reach about 1% to 10% for the various donor–acceptor cell configurations (see Supplementary Materials, Table S1). 

However, this calculation of the efficacy of (heterologous) GPI-AP transfer did not consider the (presumably low) numbers of AChE molecules (i) synthesized during the 1-week incubation by the donor cells, in particular by those having released GPI-APs, (ii) being lost during the transfer process in course of unspecific adsorption to the filter plate or plastics of the culture dishes or due to unphysiological dilution by the culture medium and (iii) being released from the acceptor cells in course of successful transfer. 

Nevertheless, these considerable portions of full-length GPI-APs transferred from donor to acceptor cells in the transwell co-culture further substantiated the conclusion about the biological function and physiological relevance of GPI-AP transfer in vivo. Furthermore, the significant differences in transfer efficacy between configurations of different donor cells (and identical acceptor cells) and the lack of differences between configurations of identical donor cells (and different acceptor cells) were in agreement with release of full-length GPI-APs from PM of donor cells rather than their insertion into PM of acceptor cells being rate-limiting for GPI-AP transfer.

For further biochemical and cell biological characterization of the intercellular transfer of GPI-APs, the effect of various agents was studied upon their addition to the insert wells of transwell co-cultures in homologous and heterologous configurations (Figure 9). Transfer of CD55, CD59 and AChE from wildtype to GPI-deficient EL cells was significantly diminished by immune depletion with the corresponding antibody-protein A Sepharose (Figure 9a,e, dark green curve and bar) either alone or in combination (ALL) and, most potently, by adsorption to α-toxin Sepharose. 

As expected, anti-Glut1-protein A Sepharose did not affect transfer (Figure 9a,e, orange curve and bar) and culture medium alone in the insert wells did not support transfer (Figure 9a, blue curve). The only partial reduction of total GPI-AP transfer in course of bacterial PI-PLC action (Figure 9e, light green curve) is explained best by limited accessibility of the GPI anchor of human GPI-APs per se (see explanation for Figure 1 and Figure 2) or due to their arrangement in PI-PLC-resistant micelle-like complexes during transfer. These findings were compatible with appearance of GPI-APs during their intercellular transfer as soluble entities in the culture medium of the insert and bottom wells.

Interestingly, phenylsepharose, which had previously been shown to strongly bind to the fatty acyl chains of the GPI anchor of GPI-APs [88,89,90], failed to block GPI-AP transfer. This is compatible with masking of the hydrophobic portion of the GPI anchor within a macromolecular assembly, a candidate of which represents the recently described micelle-like complexes that are constituted by GPI-APs and (lyso)phospholipids [31,32]. Mediation of intercellular transfer of GPI-APs by those complexes of apparently larger size rather than by monomolecular entities was further supported by its almost complete blockade in the course of using transwell co-cultures with filter plates of 0.4 µm pore size instead of 1 µm (data not shown). 

Furthermore, the observation that downregulation of transfer of a GPI-AP by immune depletion relied on the corresponding antibody (Figure 9a,e) but was not functional with antibodies against other GPI-APs (Figure 9a) suggested that each complex consists of (lyso)phospholipids and only a single species of GPI-AP rather than of several or all GPI-APs expressed at PM of the donor cells. This finding together with the heavily impaired diffusion of vesicular structures across filter plates with 1 µm pore size (G.A.M., T.D.M., unpublished observations) argued against extracellular vesicles as putative mediators of intercellular transfer.

Next, the effects of total and specific serum proteins, which have already been demonstrated to interact with GPI-APs, such as GPLD1 [31,53] or albumin (Supplementary Materials, Figure S4), on intercellular transfer of GPI-APs were investigated (Figure 9b,c,f,g). In fact, rat serum added to the insert wells led to downregulation of the transfer of GPI-APs from wildtype EL cells or untreated human adipocytes to ManN-treated adipocytes or GPI-deficient EL cells, respectively (Figure 9b,c, green curves) in volume-dependent fashion (Figure 9f, orange curve) to up to the level observed with culture medium instead of donor cells (Figure 9b, blue curve). 

Serum GPLD1 diminished transfer to up to about 70% of serum inhibition (Figure 9b, grey curve). This inhibition was further exacerbated by simultaneous presence of the Ca^2+^-chelating agent Pha (Figure 9b,f, red and black curves). This was compatible with stabilization of the interaction of GPI-APs with serum GPLD1 and possibly with other Ca^2+^-dependent serum proteins in the absence of Ca^2+^. Complete inhibition of transfer was achieved with 200 and 100 µL of serum in the absence (Figure 9f, orange curve) and presence of Pha (Figure 9f, red curve) in the culture medium, which corresponded to a final dilution of 1:75 and 1:150, respectively. 

Moreover, BSA at a concentration of 50 mg/mL, corresponding to that of albumin in human serum [91,92,93], and a final dilution of 1:75 reduced GPI-AP transfer by about 35% (Figure 9b, turquoise curve). In contrast, γ-globulin at a concentration corresponding to that in human serum and the same final dilution had no effect (Figure 9b, brown curve). Together these findings strongly suggested that serum proteins, among them GPLD1 and albumin, control the efficacy of intercellular transfer of GPI-APs by direct interaction with their anchors.

Since PIGs have already been demonstrated to cause dissociation of GPI-APs from serum proteins (Supplementary Materials, Figure S5), their effect on the intercellular transfer of GPI-APs was assayed next (Figure 9c,g). In fact, PIGs caused restoration of the transfer of TNAP, CD73 and AChE from untreated human adipocytes to GPI-deficient EL cells that had been completely blocked by serum (Figure 9c, blue curve; no endogenous expression of GPI-APs in GPI-deficient EL cells), dependent on their structure and concentration (Figure 9g), to up to the control (Figure 9c, green curve; absence of serum). PIG41 was most effective, followed by PIG37, 45, 7 and 1, as also reflected in the increasing EC_30_ in this ranking order (Figure 9g, vertical hatched lines). These data demonstrated the capability of serum proteins to block intercellular transfer of GPI-APs through interaction with the glycan core of their GPI anchor, which is overcome by excess of PIGs.

Based on previous findings about the positive correlation between the size of primary rat adipocytes as donor cells and the release of GPI-APs from their PM [82], the impact of lipid-loading of human adipocytes, as a result of lipid droplet biogenesis [94], on the intercellular transfer of GPI-APs was investigated (Figure 9d,h). In fact, the transfer of TNAP, CD73 and AChE from untreated to ManN-treated human adipocytes was found to depend on the stage of lipid-loading of both donor and acceptor cells and to significantly differ between the four configurations assayed. 

Upon correction for the endogenous PM expression of GPI-APs in heavily (Figure 9d, brown curve; stage IV) and slightly lipid-loaded (Figure 9d, red curve, stage I) adipocytes, transfer was the most and least efficient from heavily to slightly lipid-loaded adipocytes (Figure 9d,h, green curve and bars) and from slightly to slightly lipid-loaded adipocytes (Figure 9d,h, orange curve and bars), respectively, with the other two configurations in between (Figure 9d,h, blue and turquois curves and bars). Apparently, the stage of lipid-loading of human adipocytes determined the efficacy of transfer of GPI-APs between them, with heavily lipid-loaded ones acting as efficient donor cells and slightly loaded ones as efficient acceptor cells.

**Figure 10:**
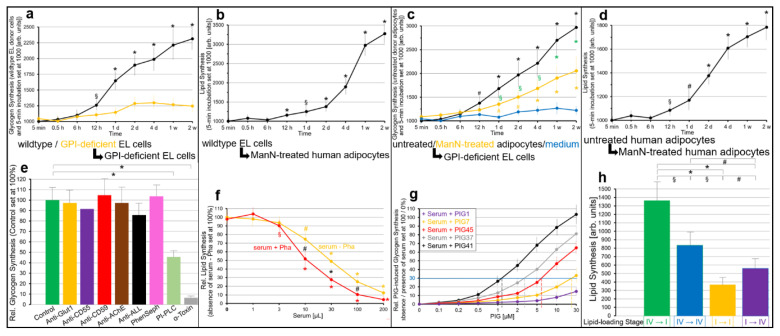
Stimulation of glycogen and lipid synthesis in acceptor cells in transwell co-culture. GPI-deficient EL cells and ManN-treated human adipocytes as acceptor cells in the bottom wells of transwell co-cultures generated above (see Figure 9) were used for determination of glycogen (see legend to Figure 9a,c) and lipid synthesis (see legend to Figure 9b,d), respectively. (**a**–**d**) The mean values ± SD (four to six independent incubations and assays, each) of the synthesized glycogen and lipid are given as arb. units set at 1000 for incubation (5 min) of wildtype donor and GPI-deficient EL acceptor cells (**a**), wildtype EL donor cells and ManN-treated acceptor adipocytes of lipid-loading stage II (**b**), untreated donor adipocytes of stage II and GPI-deficient EL acceptor cells (**c**) or untreated donor and ManN-treated acceptor adipocytes of lipid-loading stage II (**d**). Significant differences vs. 5-min incubation are indicated (black and orange symbols) for each configuration. Significant differences between untreated and ManN-treated adipocytes as donor cells are indicated (**c**, green symbols). (**e**,**f**) Phase shift Δ for each incubation of acceptor with donor cells was corrected for that with medium alone in the insert wells (Δ Medium) and used for calculation of the relative glycogen and lipid synthesis with controls (no added agent or serum) set at 100% and significant differences vs. control (**e**) or absence of serum (**f**, orange and red symbols) as well as for serum vs. serum + Pha (**f**, black symbols) indicated. (**g**) Phase shift Δ were used for calculation of the relative glycogen synthesis with absence and presence of serum set at 100% and 0%, respectively. (**h**) Phase shift Δ were corrected for those with medium alone in the insert wells (Δ Med.I/IV, Δ Medium for lipid-loading stage I or IV) for all configurations of lipid-loading stages of donor → acceptor adipocytes and used for calculation of lipid synthesis [arb. units]. Significant differences between the four different configurations of lipid-loading stage of donor → acceptor adipocytes are indicated (* *p* ≤ 0.01, ^#^ *p* ≤ 0.02, ^§^ *p* ≤ 0.05).

Finally, the same acceptor cells that had been analyzed for transfer of GPI-APs from donor to acceptor cells in homologous and heterologous configurations (Figure 8) as well as its impairment by various agents (Figure 9) were studied for glycogen and lipid synthesis for unravelling of putative correlations between transfer and synthesis stimulation (Figure 10).

Transwell co-culture of wildtype but not GPI-deficient EL cells with GPI-deficient EL cells (Figure 10a) or ManN-treated human adipocytes (Figure 10b) as well as untreated but not ManN-treated human adipocytes with GPI-deficient EL cells (Figure 10c) or ManN-treated human adipocytes (Figure 10d) led to time-dependent stimulation of glycogen (Figure 10a,c) and lipid synthesis (Figure 10b,d), respectively, in the corresponding acceptor cells. Importantly, the presence of α-toxin Sepharose (Figure 10e, grey bars) and bacterial PI-PLC (light green) completely and partially, respectively, interfered with the upregulation of glycogen synthesis. 

These data together with failure of a transwell co-culture with 0.4 µm pore size to support transfer and glycogen synthesis stimulation (G.A.M., T.D.M., unpublished observations) made the involvement of other soluble entities released from the donor cells, such as cytokines [95,96], in upregulation of syntheses in acceptor cells unlikely. Interestingly, antibodies against Glut1, CD55, CD59 and AChE alone or in combination (Anti-ALL) as well as phenylsepharose had no effect. Moreover, serum inhibited lipid synthesis in a volume-dependent fashion (Figure 10f, orange curve). This effect was further enhanced by Pha leading to a leftward shift of the serum volume–inhibition curve (Figure 10f, red curve). 

Serum inhibition of glycogen synthesis was abrogated by PIGs dependent on their concentration and structure (Figure 10g), with PIG41 > 37 > 45 > 7 >1 in that ranking order of declining efficacy. Lastly, upregulation of lipid synthesis in ManN-treated human adipocytes during transwell co-culture with untreated human adipocytes was found to depend on the stage of lipid-loading of both donor and acceptor cells (Figure 10h). A configuration of heavily and slightly lipid-loaded donor and acceptor cells, respectively, was most (Figure 10h, orange bar) and that of slightly lipid-loaded donor and acceptor cells (Figure 10, green bar) least efficient. 

Together these data demonstrated a positive correlation between the transfer of GPI-APs in the four configurations of donor and acceptor cells used in this study (Figure 9) and the stimulation of glycogen or lipid synthesis in the acceptor cells (Figure 10). Importantly, bacterial PI-PLC and α-toxin Sepharose inhibited both transfer (Figure 9e) and glycogen (Figure 10e) and lipid synthesis (G.A.M., T.D.M., unpublished observations). In contrast, antibody-protein A Sepharose directed against a given GPI-AP interfered with its transfer (Figure 9e) but not with upregulation of glycogen (Figure 10e) and lipid synthesis (G.A.M., T.D.M., unpublished observations). 

This strongly argued for mechanistic involvement of the expression at PM of certain GPI-APs, apparently not identical with CD55, CD59, AChE (Figure 10e), TNAP and CD73 (G.A.M., T.D.M., unpublished observations), in the control of basal (i.e., absence of exogenous stimuli, such as insulin) glycogen and lipid synthesis, which is controlled by the intercellular transfer of those GPI-APs. Transfer (Figure 9b,f) and, as a consequence, synthesis stimulation (Figure 10f) becomes downregulated by interaction of those GPI-APs with serum proteins, such as albumin and GPLD1, and restored by relief of those GPI-APs from this interaction (Figure 9g and Figure 10g).

## 3. Discussion

### 3.1. (Patho)Physiological Role of Intercellular Transfer of GPI-APs

Shortly after the first description of membrane anchorage of cell surface proteins by glycolipidic anchors and elucidation of their detailed structure [5,6,97,98], the possibility of their spontaneous release from and insertion into PM of donor and acceptor cells, respectively, has been raised [99,100,101]. Subsequently, both the release [102,103,104,105,106,107] and insertion [108,109,110,111,112,113,114,115] of GPI-APs were substantiated by cell biological findings that, however, did not address putative (patho)physiological implications for the cells and tissues involved. 

The present study provides a panel of experimental evidence for a functional role of the intercellular transfer of GPI-APs at the biochemical (incubation of primary rat adipocytes with total rat adipocyte GPI-APs reconstituted into micelle-like complexes; Figure 4, Figure 6 and Figure 7; Supplementary Materials, Figure S6) and cellular level (transwell co-culture of human adipocytes or EL cells as donor and acceptor cells in homologous and heterologous configurations; Figure 8, Figure 9 and Figure 10):

(i) Transfer of certain full-length GPI-APs, as exemplified by, but actually not involving CD55, CD59, CD73, TNAP and AChE (Figure 4, Figure 8 and Figure 9), to primary rat or cultured human adipocytes and cultured human EL cells with low or missing expression of GPI-APs at PM (Figure 1, Figure 2 and Figure 3) leads to stimulation of lipid and glycogen synthesis, respectively (Figure 7 and Figure 10; Supplementary Materials, Figure S6).
(ii) Transfer and syntheses are blocked by certain serum proteins that bind full-length GPI-APs, such as GPLD1 and albumin, and other thus far unidentified ones (Figure 6, Figure 7, Figure 9 and Figure 10).
(iii) This blockade is bypassed by the dissociation of GPI-APs from the binding-proteins, as provoked by synthetic PIGs (Figure 6, Figure 7, Figure 9 and Figure 10), which closely resemble the structure of the glycan core of the GPI anchor (see Supplementary Materials, Figure S2) and manage to displace rat and human serum components from micelle-like GPI-AP complexes in vitro (Supplementary Materials, Figure S5).
(iv) Rat and human adipocytes displaying large size or heavy lipid-loading, respectively, are most efficient as donor cells and those of small size or slight lipid-loading as acceptor cells (Supplementary Materials, Figure S6; Figure 10).

**Figure 11:**
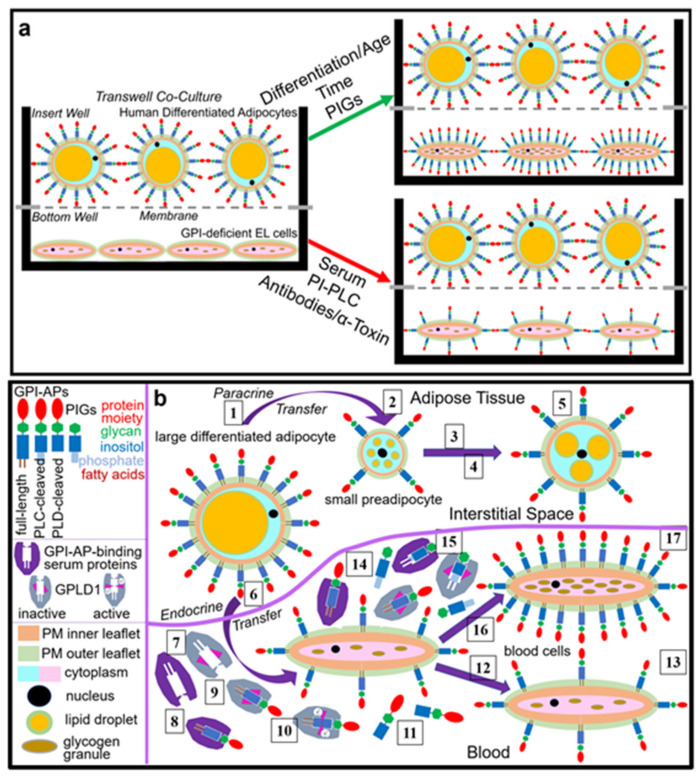
Intercellular transfer of GPI-APs. (**a**) Analysis by transwell co-culture as exemplified for the donor human adipocytes—acceptor EL cells configuration. (Left half) Human adipose-derived stem cells (hADSCs) are seeded in the top insert well, which is equipped with a bottom porous filter plate and placed onto a companion bottom well, then grown to confluency and subsequently differentiated into human adipocytes of varying stage of lipid-loading. GPI-deficient EL cells are seeded in a separate bottom well and then grown to confluency. For initiation of transfer, the insert well harboring the human adipocytes is placed on top of the bottom well harboring the GPI-deficient EL cells and then incubated for certain periods in the absence or presence of certain agents before measurement of GPI-AP expression at PM (by chip-based SAW sensing) and glycogen synthesis (by addition of U-[^14^C]glucose) by the EL cells in the bottom well (parallel wells for each incubation) after its disassembly from the companion insert well (see Materials and Methods for details). (Right upper half) Incubation of human adipocytes in the insert wells with GPI-deficient EL cells in the bottom wells together with PIGs (in case of serum in the medium) leads to time-dependent upregulation of the expression of GPI-APs at PM of the EL cells with accompanying synthesis of glycogen and its accumulation in granules. (Right lower half) Transfer and synthesis were considerably diminished in the presence of antibody-protein A against GPI-APs or α-toxin, both coupled to Sepharose, bacterial PI-PLC or serum in the medium (see [**b**] for explanation of the symbols). (**b**) Hypothetical model for the intercellular transfer of GPI-APs via endocrine and paracrine routes and its impact on lipid and glycogen synthesis of adipose cells (upper compartment, stages 1–5) and blood cells (lower compartment, stages 6–16), respectively, in response to endogenous or exogenous cues. (Upper compartment) Paracrine transfer of full-length GPI-APs within adipose tissue depots is initiated by their release from the outer leaflet of the PM of large heavily lipid-loaded adipocytes [1]. This may be facilitated by specific characteristics of the PM (e.g., viscoelasticity and stiffness), typical for the age and metabolic state of the donor cells as has been reported recently for both rat and human adipocytes [53]. Subsequently, those GPI-APs become inserted into the outer leaflet of PM of small slightly lipid-loaded (pre)adipocytes, which are located in close neighborhood at the same adipose tissue depot and initially harbor only few or no full-length GPI-APs (stage 2). This leads to considerable upregulation of their expression at PM (stage 3) as well as lipid synthesis (stage 4). The accumulation of lipids fosters lipid droplet biogenesis and thereby the growth of small to large adipocytes (stage 5). The absence or low concentrations of GPI-AP-binding serum proteins in the interstitial space of adipose tissue depots, possibly in course of high flux of the blood stream, supports the (desired) paracrine transfer of GPI-APs in unidirectional fashion. This shifts the burden of lipid-loading from large to small adipocytes within an adipose tissue depot. (Lower compartment) For the initiation of endocrine transfer from large heavily lipid-loaded adipocytes to blood cells, released full-length GPI-APs have to pass the endothelial barrier into the blood compartment (stage 6). Here, they bind to certain GPI-AP-binding serum proteins (stage 7), such as albumin (stage 8) and GPLD1 in the inactive state (lack of Ca^2+^) (stage 9), most likely through recognition of the glycan core of the GPI anchor. Active GPLD1 (presence of Ca^2+^) cleaves the GPI anchor (stage 10), thereby, removing the inositol-glycan-protein moiety of GPI-APs (stage 11). These distinct modes of interaction of serum proteins and full-length GPI-APs (stages 8–10) each prevent their insertion into PM (stage 12) and thereby block their intercellular transfer to as well as upregulation of glycogen synthesis in blood cells (stage 13). In contrast, endocrine transfer is fostered by small-molecule factors, such as PIGs, in blood (stage 14), which displace serum proteins from full-length GPI-APs (stage 15). This enables their insertion into the outer leaflet of PM of blood cells (stage 16), thereby, stimulating synthesis of glycogen and its deposition in granules (stage 17).

Together these data suggest a working model for a (patho)physiological role of the intercellular transfer of GPI-APs in general and in the control of glycogen and lipid synthesis in particular (Figure 11). The interplay of serum factors, such as “inhibitory” GPI-AP binding-proteins and “stimulatory” PIGs, may be critical for the (desired) paracrine vs. the (unwanted) endocrine routing of GPI-APs. Full-length GPI-APs en route within the interstitial space of adipose tissue depots as well as between the latter and the blood compartment are not depicted here but may be arranged in micelle-like (lyso)phospholipid complexes as was characterized previously [32].

In brief, the intercellular transfer of full-length GPI-APs seems to exert a physiological function for donor and acceptor cells within the same tissue or organ but an undesired, potentially even pathophysiological effect on acceptor cells residing at tissues or organs distant from those of the donor cells. As shown here, “paracrine” transfer of specific GPI-APs from adipocytes to adipocytes (within the same adipose tissue depot) stimulates lipid synthesis and thereby determines its basal value (i.e., in the absence of insulin), whereas “endocrine” transfer from adipocytes to cells of the blood compartment causes adverse lipid storage in the latter. The absence or presence of binding-proteins for GPI-APs in interstitial spaces and the blood compartment, respectively, may determine paracrine vs. endocrine transfer.

With regard to the binding-proteins for GPI-APs in serum, GPLD1 accounted for up to 50% of the total binding activity (in the absence of Ca^2+^), and albumin was identified as another candidate of minor capacity (Figure 10; Supplementary Materials, Figure S4). Interestingly, two decades ago, proteinaceous binding sites for a PIG-peptide, the PIG portion being identical with the authentic glycan core of yeast GPI anchors, were identified in lipid rafts of rat adipocyte PM, which were highly enriched in GPI lipids and GPI-APs [116]. 

Importantly, high affinity and specificity of binding were demonstrated by competition with synthetic PIGs displaying IC_50_ similar to the EC_50_ for stimulation of glucose transport by those PIGs. Salt washing, carbonate extraction and *N*-ethylmaleimide treatment of total rat adipocyte PM or lipid rafts led to abrogation of binding [117]. These findings together with labeling of PM with [^14^C]*N*-ethylmaleimide (NEM) led the authors to suggest that a 115-kDa NEM-sensitive polypeptide acts as a receptor for PIG(-P), which is peripherally associated with PM of insulin target cells and mediates insulin-mimetic signaling [117]. 

It will be interesting to see whether this NEM-sensitive polypeptide is also present in serum and operates as an additional serum binding-protein for full-length GPI-APs. In this case, it is conceivable that the 115-kDa NEM-sensitive polypeptide initiates or fosters the release of GPI-APs from the outer leaflet of PM in course of shuttling between the surface of donor cells and the blood compartment and thereby facilitates or even controls the transfer of GPI-APs to acceptor cells.

### 3.2. PIGs, GPI-AP Transfer and Matter vs. Information Transfer

As a byproduct of this study, its data may help to understand the heavily debated controversy about the operation of PIGs as second messengers of insulin action, which started in 1981 [118] and lasted for more than two decades (for reviews see Refs. [119,120,121]), at least in part. In fact, a role in insulin-mimetic signaling has been attributed to naturally occurring PIGs (and PIG-peptides) as the lipolytic (and proteolytic) degradation products of specific GPI lipids (and GPI-APs). 

This was based mainly on their potential to stimulate glucose and lipid metabolism in insulin target cells, such as myocytes and adipocytes, upon incubation of PIG(-peptides) of defined structure in vitro [79,122,123,124]. However, subsequent problems with the reproducibility of some of these results as well as the size of the effects yielded with different PIG preparations (including those of unknown structure) and assay systems by various laboratories discredited this hypothesis [125]. However, structurally defined PIGs as true small-molecule insulin mimetics could be of value for the therapy of diabetes mellitus type II [126,127].

The findings presented here suggest that considerable variation in the efficacy of structurally defined PIGs is explained by differential responsiveness of the target (in particular adipose and muscle) cells towards PIGs and insulin. With regard to adipocytes, specific requirements for cell size and degree of lipid-loading (Figure 8 and Figure 11) as well as specific conditions for their preparation (i.e., collagenase digestion) and incubation (i.e., shaking intensity, titer, presence of albumin or serum) have to be fulfilled, which apparently are not identical with (and sometimes even opposed to) those supporting maximal insulin action (Supplementary Materials, Figures S3 and S4). 

For instance, defatted BSA (primary cells) or certain serum fractions (cultured cells), which are typically used for cell biological experiments, support insulin activity but interfere with the detection of PIG insulin-mimetic activity—i.e., the higher their concentration, the higher the insulin and the lower the PIG activity. It is likely that detection of the latter strictly depends on serum albumin or specific serum proteins loaded with full-length GPI-APs, which are removed in course of routine procedures for commercialization, such as defatting. Exact description of the source and origin of the assay components as well as tight control of the experimental conditions are therefore of tremendous importance to guarantee valid results and correct conclusions about PIGs as functionally relevant molecules in physiologically relevant studies in the future.

In any case, according to the presented findings, PIGs cannot be regarded as insulin second messengers but rather manage to trigger insulin-mimetic action through a non-canonical pathway: They displace GPI-APs from serum proteins (Supplementary Materials, Figure S5), which makes them available for transfer to insulin target cells. This results in stimulation of lipid and glycogen synthesis in adipose and muscle cells, respectively. Thus, PIG activity depends on intercellular transfer of material rather than signals, messages, or information, which is the case for insulin. Consequently, the intercellular transfer of GPI-APs must be regarded as material rather than information transfer.

Interestingly, the current controversy about extracellular vesicles as information carriers (for reviews see Refs. [128,129,130,131]) sheds light on a similar misunderstanding of the term “information” as it is typically used with regard to hormone or neurotransmitter action. The molecules transferred by extracellular vesicles from donor to acceptor cells, such as some luminal or membrane proteins, exert biological activity by themselves as discrete matter (e.g., as enzymes, binding proteins and transporters) rather than as abstract message or order, which is dependent on deciphering by a cellular machinery (e.g., activation of a receptor or downstream signaling cascade). 

This dualism is manifested in the apparent unrelatedness of structure and function for signaling molecules, such as insulin and glucose transport stimulation. This is in contrast to the relationship of structure and function for matter, such as glycerol-3-phosphate acyltransferase and lipogenesis stimulation, transferred and mediated, respectively, by extracellular vesicles [130]. Thus, transfer of matter, which is also realized with the transfer of full-length GPI-APs from donor to acceptor cells, should be clearly distinguished from transfer of information from cells or organs that secrete signaling molecules to those that decode the message.

### 3.3. Implications for the Therapy of Metabolic Diseases

Although the experimental demonstration of the intercellular transfer of GPI-APs with resulting stimulation of glycogen and lipid synthesis at the biochemical and cellular level suggests a (patho)physiological role in the control of metabolism and development of metabolic diseases (e.g., diabetes and obesity), this speculation remains to be substantiated by future elucidation of (i) the cell types and tissues operating as donors and acceptors in vivo, (ii) the higher likelihood of paracrine vs. endocrine transfer (or vice versa) in vivo and (iii) the GPI-APs involved in transferring specific functions in vivo.

Ad (i) and (ii): It is tempting to speculate that adipose, muscle, endothelial and blood cells act as donor and acceptors cells for the regulation of lipid synthesis by transferred GPI-APs based on the presented data and the following rationale: Storage of lipids in insulin target cells and tissues that are not destined for (e.g., muscle in contrast to adipose) is regarded as cause for or consequence of their insulin-resistant state as prevalent during type II diabetes. 

This so-called lipotoxicity may be bypassed by shifting the burden of lipid storage from large, heavily lipid-loaded to small, slightly lipid-loaded adipocytes by paracrine transfer of GPI-APs. At variance, endocrine transfer could lead to lipid storage in muscle and insulin non-target cells (e.g., endothelial and blood) and thereby contribute to the development of insulin resistance and diabetic late complications, respectively. Since transfer of GPI-APs controls the basal level of glycogen and lipid synthesis in both insulin target and non-target cells, determination of the portion of full-length GPI-APs (probably embedded in micelle-like complexes) that are free in serum, i.e., not bound to serum proteins, may be useful for diagnosis, monitoring and stratification of type II diabetes.

Ad (iii): With regard to the specific GPI-APs involved, Gpc4, Gce1 and T-cadherin with their demonstrated functions in metabolic processes (see Ref. [53] for a more detailed discussion) represent valid candidates. Unfortunately, antibodies appropriate for their immune depletion have not been available so far, and thus tedious proteomic identification or large-scale genetic inactivation of the transferred GPI-APs will be required. It is reasonable to assume that such a broader analysis using appropriate animal models will uncover additional (patho)physiological roles of the intercellular transfer of GPI-APs as well as of its control by serum components and thereby unravel novel targets for therapy.

Last but not least, the demonstration of stimulation of glycogen and lipid synthesis in insulin target cells in response to transferred full-length GPI-APs opens the possibility for the therapeutic use of transfer. The relevant GPI-APs may be injected intravenously for insertion into PM of blood cells (a process previously called “cell surface engineering or painting” [115,132,133]) and subsequently be transferred to insulin target cells of other compartments, such as adipose tissues. 

This could provoke upregulation of lipid synthesis in small slightly lipid-loaded but insulin-resistant adipocytes of type II diabetic patients by bypassing the defective intracellular canonical insulin signaling without the need for gene therapy. This option may even gain attractiveness based on the preliminary experimental evidence that the GPI anchor mediates transcellular transport of proteins from apical to basolateral PM in polarized cells. 

This may enable GPI-APs to cross the intestinal and endothelial barriers and support their oral delivery [134]. Furthermore, the obvious requirement of finetuning of intercellular transfer of GPI-APs to and thus of basal lipid synthesis in adipocytes in response to exogenous factors, such as caloric intake and physical activity, may be achieved by small molecules, such as PIGs, which control the availability of GPI-APs for transfer through provoking their dissociation from serum proteins.

## 4. Materials and Methods

### 4.1. Materials

Human adipose derived stem cells (hADSC) were delivered by iXCells Biotechnologies (San Diego, USA, Cat. Nr. 10HU-001). RPMI 1640 medium was obtained from GIBCO (Thermo Fisher Scientific, Schwerte, Germany). Fetal bovine serum (FBS) was provided by HyClone Laboratories Inc. (Bath, UK). The sources of the phospholipases and antibodies (including the dilutions used) were given previously [53] unless indicated otherwise. 1-ethyl-3-[3-dimethylaminopropyl] carbodiimide (EDC) and N-hydroxysulfosuccinimide (Sulfo-NHS, premium grade) were bought from Pierce/Thermo Scientific (Rockford, IL, USA). 

Protein A-Sepharose (Cl-4B) and phenylsepharose were from Calbiochem/Merck (Darmstadt, Germany). Polystyrene Bio-Beads SM-2 (20–50 mesh) were bought from Bio-Rad Laboratories (Munich, Germany). Mannosamine (ManN, 2-amino-2-deoxy D-mannose), methyl-ß-cyclodextrin (mßCD), BSA (fraction V, defatted) and human serum (normal healthy probands) were delivered by Sigma-Aldrich (Deisenhofen, Germany). Human (recombinant) insulin was a kind gift from Sanofi Pharma Germany GmbH (Diabetes group, Frankfurt am Main, Germany). Other materials (highest purity available) were obtained as described previously [31,32,53,82,83].

### 4.2. Culture of Wild-Type and GPI-Deficient K562 Erythroleukemia (EL) Cells

Wild-type and mutant (incapable of the earliest step in the glycosylation of PI, i.e., coupling of acetylated glucosamine) EL cells [57,58,59] were grown in RPMI 1640 medium supplemented with 10% FBS and 1% penicillin/streptomycin (0.3–1.2 × 10^6^ cells/mL). Prior to assaying of GPI-AP transfer or glycogen/lipid synthesis, the cells were washed three times with Ca^2+^-free PBS, resuspended in serum-free medium containing 0.1% BSA and incubated (4 h, 37 °C). Thereafter, the cells were centrifuged (250× *g*, 2 min, 24 °C) and then resuspended (at 5 × 10^6^ cells/mL) in medium free of serum and BSA for incubation with GPI-APs in standard cell culture or as donor / acceptor cells in Transwell co-culture (see below).

### 4.3. Differentiation and Culture of Human Adipocytes

hADSCs were isolated from lipoaspirate tissue from single normal donors collected during elective surgical liposuction procedures, cryopreserved at passage 1 (1.0 million cells/vial) and characterized by iXCells Inc. (San Diego, CA, USA) [69,70]. hADSCs were further expanded in hADSCs Growth Medium (iXCells Inc.) for three to four passages as described in detail previously [53]. Finally, new culture flasks (12-well plate formate) were seeded at 5 × 10^3^ cells/cm^2^ with media change every 2–3 days until the cells had reached 70–80% confluence.

hADSCs were differentiated into human adipocytes in vitro using Adipocytes Differentiation Medium (iXCells Inc.) as reported previously [53]. hADSCs were regarded as human adipocytes of slight (stage I), medium (stage II) or heavy lipid-loading (stage IV) when Oil Red-stained lipid droplets accounted for 10–25%, 45–60% and more than 80%, respectively, of the cytoplasmic area. 

The stage of lipid-loading was maintained using hADSCs Growth Medium supplemented with 1 µM dexamethasone, 0.5 mM isobutylmethylxanthine and 1 µg/mL insulin, with replacement every 48 h. Following washing with Dulbecco’s Modified Eagle’s Medium (DMEM, Gibco-BRL, Thermo Fisher Scientific, Waltham, MA, USA) containing 1% sodium pyruvate, 100 U/mL of penicillin and 100 µg/mL of streptomycin, the human adipocytes were used for incubation with GPI-APs (test system [i]) or for Transwell co-culture (test system [ii], see below).

### 4.4. Mannosamine (ManN) Treatment of Human Adipocytes

Human adipocytes were incubated (36 h, 37 °C) in glucose-free (glucose was shown to prevent uptake of ManN into MDCK cells [64]) medium (RPMI 1640) containing 10% FBS, 20 mM sodium pyruvate and 10 mM sodium butyrate in the absence or presence of ManN (prepared from a 1 M sterile-filtered stock with deionized H_2_O, then frozen at −20 °C in aliquots and finally thawed and diluted with culture medium immediately before use) at the concentrations indicated. 

Previous studies revealed that these conditions are optimal for inhibition of N-linked glycan synthesis in MDCK cells [65]. Control experiments demonstrated that glucosamine at identical concentrations and conditions has no significant effect on the expression of GPI-APs at PM of human adipocytes (data not shown).

### 4.5. Preparation of Primary Rat Adipocytes from Epididymal Fat Pads

See Supplementary Materials: Materials and Methods S1.

### 4.6. Methyl-ß-Cyclodextrin (mßCD) Treatment of Primary Rat Adipocytes

For cholesterol deprivation of primary rat adipocytes suspended in adipocyte buffer at a lipocrit of 10% (see Section 4.5), they were incubated (22 °C) with various concentrations of mßCD, freshly dissolved in the same buffer as 100-mM stock solution, for various periods of time as indicated. As a control, 2 mM mßCD-cholesterol inclusion complexes (2 mM mßCD-chol.) were used, which were prepared as described previously [71]. In brief, 30 mg of cholesterol were dissolved in 0.5 mL of methanol/chloroform (2/1 *v*/*v*) and then added in small aliquots to 1 g of mßCD in solution (5% *v*/*v*) at 80 °C. The mixture was stirred at 80 °C until complete dissolution of the initially precipitating steroid indicating its stable complexing by mßCD. 2 mM mßCD-chol. were stored at 22 °C until use on the same day.

This protocol resulted in the following depletion of cholesterol from the adipocyte PM as revealed by their subsequent preparation and enzymic determination of cholesterol: 15.3% (0.5 mM mßCD), 28.9% (1 mM mßCD), 67.7% (2 mM mßCD) and 2.4% (2 mM mßCD-chol.) for 10-min incubation period, 7.4% (2-min incubation period), 33.6% (5-min incubation period), 74.9% (10-min incubation period) and 89.8% (30-min incubation period) for 1 mM mßCD. Cholesterol depletion was paralleled by the following losses of cell viability, as assessed by trypan blue exclusion (counting of 500 cells three times each): 2.6% (0.5 mM mßCD), 4.9% (1 mM mßCD), 9.8% (2 mM mßCD) and 3.5% (2 mM mßCD-chol.) for 10-min incubation period, 2.9% (2-min incubation period), 6.0% (5-min incubation period), 11.2% (10-min incubation period) and 35.9% (30-min incubation period) for 1 mM mßCD.

### 4.7. Transwell Co-Culture of EL Cells and Human Adipocytes

Test system (ii) with transwell co-cultures was used between acceptor cells seeded at the bottom of 12-well tissue culture plates (Falcon Companion TC Plate, No. 353503, Falcon/Corning, Tewksbury, MA, USA, for 1.4–2.3 mL medium) and donor cells seeded in 12-well cell culture inserts (Falcon Cell Culture Insert, No. 353103, for 0.4–1.0 mL medium), thereby, enabling detection of the transfer of full-length GPI-APs between donor and acceptor cells of different type in different configurations as indicated, at a distance (from the membrane to the bottom of wells) of 0.9 mm through a porous membrane (pore size 1.0 µm, high pore density 1.6 ± 0.6 × 10^6^ pores/cm^2^, polyethylene terephthalate track-etched, transparent).

EL cells or hADSCs were seeded in the transwell inserts or bottom wells as required and then grown to confluence or differentiated into human adipocytes, respectively. Subsequently, 2 mL of prewarmed culture medium were added to each well of a 12-well tissue culture plate. Thereafter, a cell culture insert was removed from the package with sterile forceps and then gently placed into the bottom companion well culture plate, taking care to avoid trapping air under the insert by tilting the insert while lowering it onto the well. 

Upon correct positioning, the inserts with the flanges rested in the notches on the top edge of each well in diagonal arrangement. For seeding, the cells and 1 mL of medium were added to the cell culture insert at the density given above and cultured under routine conditions. For feeding, the inserts were first slid to one side using a sterile 1-mL Pasteur pipet to remove media from above and below the membrane. 

Subsequently, 2 and 1 mL of fresh medium lacking serum and BSA (as indicated) were added to the wells of the bottom companion tissue culture plate and cell culture insert, respectively. Following incubation at the conditions indicated, the insert wells were removed and then the medium was aspirated from the bottom wells. Thereafter, the acceptor cells of the bottom wells were rinsed with 2 × 1 mL PBS and then used for the preparation of PM (see Section 4.8) to analyze the expression of GPI-APs (see Section 4.14) as measure for GPI-AP transfer or incubated with fluorescently labeled fatty acids (NBD-FA) or D-[U-^14^C]glucose for assaying lipid or glycogen synthesis, respectively, (see Supplementary Materials).

### 4.8. Preparation of PM

PM were prepared from EL cells and human adipocytes, which were cultured in standard (test system [i], see Section 4.11) or transwell co-culture (test system [ii], see Section 4.7) 12-well culture dishes. For this, the medium was aspirated from the normal or bottom companion tissue culture plate, respectively, taking care to avoid touching the membrane of the latter. Thereafter, the top and then the bottom of the wells were rinsed with 2 × 0.5 mL PBS each to remove any serum (containing protease inhibitors). After the addition of 0.5 mL of trypsin/EDTA to the wells, the plate was incubated in a 37 °C-incubator containing 5% CO_2_ until cells were rounded and floated. 

The required period varied, depending on the degree of adherence and confluency of the cultured cells. Cell detachment was periodically monitored under a microscope. Once the cells looked rounded and detached, 0.5 mL of quenching solution was added to each well of the plate to terminate trypsinization. The suspension was gently pipetted up and down to break up cell clumps and then transferred to 2-mL Eppendorf cups. The top and bottom of the wells of the plate were rinsed with 0.5 mL of PBS each to remove any remaining cells. The rinsing fluids were combined with the cell suspension in the 2-mL Eppendorf cups and used for the preparation of PM.

PM from trypsinized human EL cells and human adipocytes were prepared using the “Minute^TM^ Plasma Membrane Protein Isolation and Cell Fractionation Kit” (Invent Biotechnologies Inc., Plymouth, UK; Cat. Nr. SM-005). After trypsinization, the suspension of adipocytes or EL cells were incubated (on ice, 10 min), vortexed vigorously (45 s) and then transferred to the filter cartridge. The cartridge was closed and centrifuged (16,000× *g*, 30 s, 4 °C, Eppendorf 5415C table top microcentrifuge). The pelleted PM (typically 150–200 μg protein) were suspended in 1 mL of 10 mM Mops/KOH (pH 7.5), 150 mM NaCl, 0.2 mM EGTA containing protease inhibitor mix (Complete, Roche, Mannheim, Germany) and stored in liquid N_2_ until use.

PM from primary rat adipocytes were prepared as described by Kiechle and coworkers [118] with minor modifications as introduced previously [135]. Pelleted PM were suspended in 10 mM Mops/KOH (pH 7.4), 0.25 M sucrose, 150 mM NaCl and 1 mM EDTA at 5 mg protein/mL, frozen in liquid N_2_ and stored until use at −80 °C.

### 4.9. Preparation of Total GPI-APs from Rat Adipocytes

Portions of 0.5 mL of rat adipocyte PM (5 mg protein/mL) were adjusted to 1.5 mL with 10 mM Tris/HCl (pH 7.4), 150 mM NaCl, 0.2 mM PMSF, containing protease inhibitor mix (Complete, Roche, Mannheim, Germany), then left on ice (15 min), subsequently added to 6 mL of ice-cold 2.5% (*w*/*v*) TX-114 (prepared by dissolving 37.5 g of TX-114 in 1 L of 10 mM Tris/HCl, pH 7.5, 150 mM NaCl on ice, precondensation at 37 °C, centrifugation and use of the TX-114-enriched lower phase), mixed thoroughly and finally incubated (37 °C, 5 min) for the initiation of clouding. 

The detergent-enriched and depleted phases were separated by centrifugation (15,000× *g*, 2 min, 25 °C). The lower TX-114-enriched phase (6 mL) was removed without any disturbance of the interface, transferred to a new tube and supplemented with TX-114 to a final concentration of 2.0% (*v*/*v*) for a second cycle of partitioning. After mixing and sequential incubation (0 °C, 5 min; 30 °C, 3 min), the solution was centrifuged (3000× *g*, 3 min). Thereafter, 5 mL of the lower TX-114-enriched phase were carefully transferred to a new tube avoiding any disturbance of the interface and then adjusted to 25 mL final volume with 10 mM Tris/HCl (pH 7.4), 150 mM NaCl, 6 mM octyl glucoside. 

After addition of 2.5 mL of α-toxin Sepharose-beads, the mixture was incubated (16 h, 4 °C, head-to-tail rotation). Following centrifugation (10,000× *g*, 5 min, 4 °C), the collected beads were suspended in 25 mL of 10 mM Tris/HCl (pH 7.4), 150 mM NaCl, 6 mM octyl glucoside, 1 mM EGTA, 0.2 mM PMSF, protease inhibitor mix (Complete, Roche, Mannheim, Germany). The washing cycle was repeated three times. The finally collected beads were suspended in 1 mL of the same buffer. The mixture was supplemented with 250 µL of 150 µM PIG41, then incubated (1 h, 4 °C) and finally centrifuged (10,000× *g*, 5 min, 4 °C). The supernatant containing full-length GPI-APs was dialyzed (membrane exclusion limit 2 kDa, same buffer, overnight, 4 °C), frozen in liquid N_2_ and stored at −80 °C until use.

### 4.10. Reconstitution of Micelle-like GPI-AP and AChE/CD73 Complexes

Preparation of bAChE and rCD73 and their reconstitution into micelle-like GPI-AP complexes were described previously [32]. Micelle-like total rat adipocyte GPI-AP complexes were produced in analogous fashion by using total GPI-APs from rat adipocytes. 1 Arb. unit of complexes corresponded to reconstitution of bAChE, rCD73 or total rat adipocyte GPI-APs, which were prepared from 10^6^ erythrocytes, 0.5 mg rat liver and 10^5^ rat adipocytes, respectively.

### 4.11. Incubation of EL Cells and Human/Rat Adipocytes with Micelle-like GPI-AP Complexes

For test system (i), EL cells (0.2–0.5 × 10^6^ cells) were grown (37 °C) in 0.5 mL of RPMI 1640 medium supplemented with 10% FBS and 1% penicillin/streptomycin, then washed two times with PBS and finally resuspended in serum-free medium. Human adipocytes (in 12-well plate format) were grown (37 °C) in 1.5 mL of ADSCs Growth Medium (lacking serum). 

After incubation (37 °C) with various amounts of micelle-like GPI-AP complexes for various periods of time as indicated, the cells were washed two times with 2 mL of serum-free medium containing 0.5% BSA and once with PBS (0.5 mL per well) and then used for the preparation of PM (see Section 4.8) to analyze the expression of GPI-APs (see Section 4.14) as measure for GPI-AP transfer or incubated with fluorescently labeled fatty acids (NBD-FA) or D-[U-^14^C]glucose for assaying lipid or glycogen synthesis (see Supplementary Materials).

Primary rat adipocytes (0.5 × 10^5^ cells) were incubated (37 °C) with various amounts of micelle-like GPI-AP complexes for various periods of time as indicated in 1 mL of adipocyte buffer (see Supplementary Materials). Thereafter, the adipocytes were washed three times with 2 mL of adipocyte buffer each by flotation and suction (250× *g*, 2 min). After final suction of the infranatant medium, the floating adipocytes were used for preparation of PM (see Section 4.8) to analyze the expression of GPI-APs (see Section 4.14) as measure for GPI-AP transfer or incubated with fluorescently labeled fatty acids (NBD-FA) for assaying lipid synthesis (see Supplementary Materials).

### 4.12. Immune Depletion of Medium or Micelle-like GPI-AP Complexes from AChE/CD73

α-Toxin was prepared from the culture supernatant of *Clostridium septicum* [136] and coupled to Sepharose beads using a conventional EDC/NHS-based protocol as described previously [31,32]. Anti-CD73 (2.5 µg protein/sample) and/or anti-AChE (10 µg protein/sample) antibodies were supplemented with 1 mL of TES buffer (20 mM Tris/HCl, pH 7.4, 1 mM EDTA, 150 mM NaCl) containing 50 mg protein A-Sepharose beads and then incubated (4 °C, overnight) in 1-mL Eppendorf cups under head-to-tail rotation. 

After centrifugation (12,000× *g*, 2 min, 4 °C), the collected beads were washed two times with 1 mL each of TEST buffer (TES buffer containing 1% TX-100, 10 µg/mL aprotinin, 1 mM benzamidine, 0.1 mM PMSF), recentrifuged, then washed four times with 1 mL of TES buffer containing 0.1% TX-100 each, recentrifuged and finally washed two times with 1 mL of TES buffer. The final pellet was suspended in 100 µL of TES buffer and stored at 4 °C until use.

For depletion of culture medium, α-toxin Sepharose beads or anti-CD73/AChE Sepharose beads were added to standard cell cultures or transwell co-cultures (insert wells) at 1:100 dilution as indicated. For depletion of micelle-like rat adipocyte GPI-AP complexes, total rat adipocyte GPI-APs prepared from 0.5 mg of PM protein were suspended in 100 µL of TEST buffer and incubated (overnight, 4 °C) with 100 µL of anti-CD73/AChE Sepharose beads in 0.5-mL Eppendorf cups under head-to-tail rotation. After centrifugation (12,000× *g*, 2 min, 4 °C), the supernatant containing full-length rat adipocyte GPI-APs depleted of CD73 or AChE were frozen in liquid N_2_ and stored at −80 °C until use for reconstitution.

### 4.13. Assay of Transfer of GPI-APs from Donor to Acceptor PM In Vitro

400 μL of PM from EL cells or human/rat adipocytes (0.2 mg protein/mL) (at 800–1200 s) was injected at a flow rate of 60 μL/min into chips with rat or human erythrocyte or adipocyte acceptor PM consecutively immobilized by ionic and covalent capture (see below). For initiation of transfer of GPI-APs from the donor PM presented in the chip microchannels as vesicles in solution to the acceptor PM immobilized at the chip TiO_2_ surface, the chips were incubated (1 h, from 1200 to 4800 s, 37 °C) at flow rate 0 (double hatched lines) in the absence or presence of certain agents for putative interference with transfer as indicated. 

For removal of the donor PM and any soluble or complex-bound GPI-APs from the microchannels, the chips were washed two times with 150 μL of PBSE each at a flow rate of 180 μL/min and then two times with 150 μL of 10 mM HEPES/NaOH, 150 mM NaCl (pH 7.5) (washing buffer) each at the same flow rate. Subsequently, the expression of GPI-APs at the acceptor PM was analyzed (see Section 4.14).

### 4.14. Analysis of PM for the Expression of GPI-APs and Transmembrane Proteins by Chip-Based SAW Sensing

PM were immobilized at the chip surface by ionic and covalent capture (see Section 4.15) and then assayed for the expression of GPI-APs and transmembrane proteins by sequential injection of 75 μL of appropriate antibodies (diluted as given in Ref. [53]) at a flow rate of 15 μL/min according to the order indicated in the figures. Finally, for demonstration of anchorage at the acceptor PM by GPI, 75 μL of PI-PLC (*Bacillus cereus*, 5 ng) at a flow rate of 15 μL/min were injected, followed by injection of three portions of 220 μL of 0.1% (*w*/*v*) Triton X-100, 10 mM glycine (pH 12) each at a flow rate of 200 μL/min for regeneration of the chips (for up to 12 re-uses). Phase shifts are given upon correction for unspecific interaction (no acceptor PM) and normalization for the varying capturing efficacy of different chips for the acceptor PM [53]).

### 4.15. Immobilization of PM at SAW Chip Surface by Ionic and Subsequent Covalent Capture

For ionic capture, uncoated negatively charged and highly hydrophilic TiO_2_ chips were used. Immobilization of erythrocyte/adipocyte PM containing positively charged, negatively charged or zwitterionic phospholipids or combinations thereof with high efficacy was performed in the presence of 2 mM Ca^2+^ in 10 mM HEPES/NaOH (pH 7.5), 100 mM NaCl to enable salt bridges between the chip surface and the PM phospholipids. PM (0.2 mg protein/mL) were injected at a flow rate of 25 μL/min for 4 min at 30 °C. After termination of the flow for 20 min at 30 °C, the chip was washed with 10 mM HEPES/NaOH (pH 7.5), 100 mM NaCl at a flow rate of 150 μL/min for 20 min at 30 °C.

For subsequent covalent capture via the protein moieties of GPI-APs as well as extracellular protein domains of transmembrane proteins, the microfluidic channels of uncoated chips were primed with three injections of 250 μL each of immobilization buffer at a flow rate of 50 μL/min. Then, the chip surface was activated by a 250-μL injection of 0.2 M EDC and 0.05 M Sulfo-NHS (mixed from 2x-stock solutions right before injection) at a flow rate of 50 μL/min. 

After a waiting period of 3 min (flow rate zero) and subsequent washing of the channels with two 300-μL portions of PBS containing 2.5 mM EGTA (PBSE) at a flow rate of 180 μL/min, the residual activated groups on the chip surface were capped by injecting 200 μL of 1 M ethanolamine (pH 8.5) at a flow rate of 60 μL/min. Thereafter, the chips were washed two times with 125 μL of PBSE each at a flow rate of 150 μL/min and then two times with 160 μL of 10 mM HEPES/NaOH (pH 7.5) each at the same flow rate.

### 4.16. Assay for Glycogen Synthesis with EL Cells

See Supplementary Materials: Materials and Methods S2.

### 4.17. Assay for Lipid Synthesis with Human and Rat Adipocytes

See Supplementary Materials: Materials and Methods S3.

### 4.18. Digestion with Phospholipases

See Supplementary Materials: Materials and Methods S4.

### 4.19. Statistical Analysis

All numerical data were presented as the means ± standard deviations (SD). Statistical significance was calculated using GraphPad Prism6 software (version 6.0.2, GraphPad Software Inc., San Diego, CA, USA) on the basis of either a two-tailed unpaired *Student’s t*-test between two experimental groups or one-way ANOVA performed with *Tukey’s* post-test for multiple comparisons. *p* ≤ 0.05 was considered to be significant.

### 4.20. Miscellaneous

The chemical synthesis of PIGs (for structure, see Supplementary Materials, Figure S2) [56], covalent coupling of α-toxin (*Staphylococcus aureus*) to protein A Sepharose beads [53], protein determination [136], and SAW sensing with long-chain 3D CM-dextran Sam^®^ 5 chips using a SamX instrument (SAW/Nanotemper, Bonn/Munich, Germany) and computer-based evaluation [31,32] were performed as previously described in detail.

## Data Availability

The datasets generated and analyzed during the current study are available from the corresponding author (G.A.M.; guenter.mueller@helmholtz-muenchen.de) on reasonable request and will be provided as the original SAW data files together with the appropriate SAW Inc. software for data visualization and processing (correction and normalization), if required, under consideration of the relevant conditions for licensing of FitMaster^®^, SensMaster^®^ and SequenceMaster^®^.

## Supplementary Materials

The following supporting information can be downloaded at: https://www.mdpi.com/article/10.3390/ijms23137418/s1, [32,53,56,63,82,83,135,137,138,139,140,141,142,143,144].

## References

[1] UniProt Consortium (2015). UniProt: A hub for protein information. Nucleic Acids Res..

[2] Eisenhaber B., Bork P., Eisenhaber B. (2001). Post-translational GPI lipid anchor modification of proteins in kingdoms of life: Analysis of protein sequence data from complete genomes. Protein Eng..

[3] Kinoshita T. (2020). Biosynthesis and biology of mammalian GPI-anchored proteins. Open Biol..

[4] Muniz M., Riezman H. (2016). Trafficking of glycosylphosphatidylinositol anchored proteins from the endoplasmic reticulum to the cell surface. J. Lipid Res..

[5] Ferguson M.A.J., Haldar K., Cross G.A.M. (1985). *Trypanosoma brucei* variant surface glycoprotein has a *sn*-1,2-dimyristyl glycerol membrane anchor at its COOH terminus. J. Biol. Chem..

[6] Haldar K., Ferguson M.A.J., Cross G.A.M. (1985). Acylation of a *Plasmodium falciparum* merozoite surface antigen via *sn*-1,2-diacyl glycerol. J. Biol. Chem..

[7] Lebreton S., Zurzolo C., Paladino S. (2018). Organization of GPI-anchored proteins at the cell surface and its physiopathological relevance. Crit. Rev. Biochem. Mol. Biol..

[8] Paulik M.G., Bertozzi C.R. (2008). The glycosylphosphatidylinositol anchor: A complex membrane-anchoring structure for proteins. Biochemistry.

[9] Kinoshita T., Fujita M., Maeda Y. (2008). Biosynthesis, remodeling and functions of mammalian GPI-anchored proteins. Rec. Prog. J. Biochem..

[10] Fujita M., Kinoshita T. (2012). GPI anchor remodeling: Potential functions of GPI anchors in intracellular trafficking and membrane dynamics. Biochem. Biophys. Acta.

[11] Maeda Y., Kinoshita T. (2011). Structural remodeling, trafficking and functions of glycosylphosphatidylinositol-anchored proteins. Prog. Lipid Res..

[12] Nakano M., Sabido-Bozo S., Okazaki K., Aguilera-Romero A., Rodriguez-Gallardo S., Cortes-Gomez A., Lopez S., Ikeda A., Funato K., Muniz M. (2021). Structural analysis of the GPI glycan. PLoS ONE.

[13] Yoko-O T., Umemura M., Komatsuzaki A., Ikeda K., Ichikawa D., Takase K., Kanzawa N., Saito K., Kinoshita T., Taguchi R. (2018). Lipid moiety of glycosylphosphatidylinositol-anchored proteins contributes to the determination of their final destination in yeast. Genes Cells.

[14] Yi-Shi L., Fujita M. (2020). Mammalian GPI-anchor modifications and the enzymes involved. Biochem. Soc. Trans..

[15] Müller G., Jung C., Wied S., Welte S., Jordan H., Frick W. (2001). Redistribution of glycolipid raft domain components induces insulin-mimetic signaling in rat adipocytes. Mol. Cell. Biol..

[16] Van den Berg C.W., Cinek T., Hallett M.B., Horejsi V., Morgan B.P. (1995). Exogenous glycosyl phosphatidylinositol-anchored CD59 associates with kinases in membrane clusters on U937 cells and becomes Ca(2+)-signaling competent. J. Cell Biol..

[17] Fujihara Y., Ikawa M. (2016). GPI-AP release in cellular, developmental, and reproductive biology. J. Lipid Res..

[18] Müller G.A. (2018). The release of glycosylphosphatidylinositol-anchored proteins from the cell surface. Arch. Biochem. Biophys..

[19] Nosjean O., Briolay A., Roux B. (1997). Mammalian GPI proteins: Sorting, membrane residence and functions. Biochem. Biophys. Acta.

[20] Müller G.A. (2018). Glycosylphosphatidylinositol-Anchored Proteins and Their Release from Cells—From Phenomenon to Meaning.

[21] Rooney I.A., Heuser J.E., Atkinson J.P. (1996). GPI-anchored complement regulatory proteins in seminal plasma. An analysis of their physical conditions and the mechanisms of their binding to exogenous cells. J. Clin. Investig..

[22] Rabesandratana H., Toutant J.P., Reggio H., Vidal M. (1998). Decay-accelerating factor (CD55) and membrane inhibitor of reactive lysis (CD59) are released within exosomes during in vitro maturation of reticulocytes. Blood.

[23] Müller C., Jung C., Straub J., Wied S., Kramer W. (2009). Induced release of membrane vesicles and exosomes from rat adipocytes containing lipid droplets, lipid rafts and glycosylphosphatidylinositol-anchored proteins. Cell. Signal..

[24] Eaton S. (2008). Multiple roles for lipids in the Hedgehog signalling pathway. Nat. Rev. Mol. Cell Biol..

[25] Eliakim R., Alpers D.H., Oren R., Fich A., DeSchryver-Kecskemeti K. (1996). Abundance of surfactant-like particles reflects mucosal integrity in patients with peptic ulcer disease. Gut.

[26] Mahmood A., Engle M.J., Alpers D.H. (2002). Secreted intestinal surfactant-like particles interact with cell membranes and extracellular matrix proteins in rats. J. Physiol..

[27] Neumann S., Harterink M., Sprong H. (2007). Hitch-hiking between cells on lipoprotein particles. Traffic.

[28] Panakova D., Sprong H., Marois E., Thiele C., Eaton S. (2005). Lipoprotein particles are required for Hedgehog and Wingless signalling. Nature.

[29] Patton S., Huston G.E. (1986). A method for isolation of milk fat globules. Lipids.

[30] Väkevä A., Jauhiainen M., Ehnholm C., Lehto T., Meri S. (1994). High density lipoproteins can act as carriers of glycophosphoinositol lipid-anchored CD59 in human plasma. Immunology.

[31] Müller G.A., Herling A.W., Stemmer K., Lechner A., Tschöp M.H. (2019). Chip-based sensing for release of unprocessed cell surface proteins in vitro and in serum and its (patho)physiological relevance. Am. J. Physiol. Endocrinol. Metab..

[32] Müller G.A., Tschöp M.H., Müller T.D. (2020). Upregulated phospholipase D activity toward glycosylphosphatidylinositol-anchored proteins in micelle-like serum complexes in metabolically deranged rats and humans. Am. J. Physiol. Endocrinol. Metab..

[33] Müller G.A. (2020). Membrane insertion and intercellular transfer of glycosylphosphatidylinositol-anchored proteins: Potential therapeutic applications. Arch. Physiol. Biochem..

[34] Johnstone R.M., Ahn J. (1990). A common mechanism may be involved in the selective loss of plasma membrane functions during reticulocyte maturation. Biomed. Biochim. Acta.

[35] Geminard C., Nault F., Johnstone R.M., Vidal M. (2001). Characteristics of the interaction between Hsc70 and the transferrin receptor in exosomes released during reticulocyte maturation. J. Biol. Chem..

[36] Johnstone R.M. (2006). Exosomes biological significance: A concise review. Blood Cells Mol. Dis..

[37] Johnstone R.M., Mathew A., Mason A.B., Teng K. (1991). Exosome formation during maturation of mammalian and avian reticulocytes: Evidence that exosome release is a major route for externalization of obsolete membrane proteins. J. Cell Physiol..

[38] Pan B.T., Blostein R., Johnstone R.M. (1983). Loss of the transferrin receptor during the maturation of sheep reticulocytes in vitro. An immunological approach. Biochem. J..

[39] Johnstone R.M., Adam M., Hammond J.R., Orr L., Turbide C. (1987). Vesicle formation during reticulocyte maturation. Association of plasma membrane activities with released vesicles (exosomes). J. Biol. Chem..

[40] Johnstone R.M., Bianchini A., Teng K. (1989). Reticulocyte maturation and exosome release: Transferrin receptor containing exosomes shows multiple plasma membrane functions. Blood.

[41] Kirchhoff C., Pera I., Derr P., Yeung C.H., Cooper T. (1997). The molecular biology of the sperm surface. Post-testicular membrane remodelling. Adv. Exp. Med. Biol..

[42] Ilangumaran S., Robinson P.J., Hoessli D.C. (1996). Transfer of exogenous glycosylphosphatidylinositol (GPI)-linked molecules to plasma membranes. Trends Cell Biol..

[43] Bütikofer P., Kuypers F.A., Xu C.M., Chiu D.T., Lubin B. (1989). Enrichment of two glycosylphosphatidylinositol-anchored proteins, acetylcholinesterase and decay accelerating factor, in vesicles released from human blood cells. Blood.

[44] Zhang H., Jones R., Martin-DeLeon P.A. (2004). Expression and secretion of rat SPAM1(2B1 or PH-20) in the epididymis: Role of testicular lumicrine factors. Matrix Biol. J. Internat. Soc. Matrix Biol..

[45] Griffiths G.S., Galileo D.S., Aravindan R.G., Martin-DeLeon P.A. (2009). Clusterin facilitates exchange of glycosyl phosphatidylinositol-linked SPAM1 between reproductive luminal fluids and mouse and human sperm membranes. Biol. Reprod..

[46] Queiroz K.C.S., Tio R.A., Zeebregts C.J., Bijlsma M.F., Zijlstra F., Badlou B., de Vries M., Ferreira C.V., Spek C.A., Peppelenbosch M.P. (2010). Human plasma very low density lipoprotein carries Indian Hedgehog. J. Prot. Res..

[47] DeSchryver-Kecskemeti K., Eliakim R., Carroll S., Stenson W.F., Moxley M.A., Alpers D.H. (1989). Intestinal surfactant-like material. A novel secretory product of the rat enterocyte. J. Clin. Investig..

[48] Eaton S. (2006). Release and trafficking of lipid-linked morphogens. Curr. Opin. Genet. Dev..

[49] Kirchhoff C., Hale G. (1996). Cell-to-cell transfer of glycosylphosphatidylinositol-anchored membrane proteins during sperm maturation. Mol. Hum. Reprod..

[50] Kooyman D.L., Byrne G.W., McClellan S., Nielsen D., Tone M., Waldmann H., Coffman T.M., McCurry K.R., Platt J.L., Logan J.S. (1995). In vivo transfer of GPI-linked complement restriction factors from erythrocytes to endothelium. Science.

[51] Liu T., Li R., Pan T., Liu D., Petersen R.B., Wong B.-S., Gambetti P., Sun Sy M. (2002). Intercellular transfer of the cellular prion protein. J. Biol. Chem..

[52] Watanabe K., Salomon D.S. (2010). Intercellular transfer regulation of the paracrine activity of GPI-anchored Cripto-1 as a Nodal co-receptor. Biochem. Biophys. Res. Commun..

[53] Müller G.A., Tschöp M.H., Müller T.D. (2021). Chip-based sensing of the intercellular transfer of cell surface proteins: Regulation by the metabolic state. Biomedicines.

[54] Andrä J., Böhling A., Gronewold T.M.A., Schlecht U., Perpeet M., Gutsmann T. (2008). Surface acoustic wave biosensor as a tool to study the interaction of antimicrobial peptides with phospholipid and lipopolysaccharide model membranes. Langmuir.

[55] Gronewold T.M.A., Glass S., Quandt E., Famulok M. (2005). Monitoring complex formation in the blood-coagulation cascade using aptamer-coated SAW sensors. Biosens. Bioelectron..

[56] Frick W., Bauer A., Bauer J., Wied S., Müller G. (1998). Structure-activity relationship of synthetic phosphoinositolglycans mimicking metabolic insulin action. Biochemistry.

[57] Hirose S., Mohney R.P., Mutka S.C., Ravi L., Singleton D.R., Perry G., Tartakoff A.M., Medof M.E. (1992). Derivation and characterization of glycoinositol-phospholipid anchor-defective human K562 cell clones. J. Biol. Chem..

[58] Mohney R.P., Knez J.J., Ravi L., Sevlever D., Rosenberry T.L., Hirose S., Medof M.E. (1994). Glycoinositol phospholipid anchor-defective K562 mutants with biochemical lesions distinct from those in Thy-1^-^ murine lymphoma mutants. J. Biol. Chem..

[59] Kamitani T., Chang H.-M., Rollins C., Waneck G.L., Yeh E.T.H. (1993). Correction of the class H defect in glycosylphosphatylinositol anchor biosynthesis in Ltk^-^ cells by a human cDNA clone. J. Biol. Chem..

[60] Stieger S., Brodbeck U. (1991). Glycosylphosphatidylinositol anchored acetylcholinesterase as substrate for phosphatidylinositol-specific phospholipase C from Bacillus cereus. Biochimie.

[61] Bon S., Rosenberry T.L., Massoulie J. (1991). Amphiphilic, glycophosphatidylinositol-specific phospholipase C (PI-PLC)-insensitive monomers and dimers of acetylcholinesterase. Cell. Mol. Neurobiol..

[62] Richier P., Arpagaus M., Toutant J.P. (1992). Glycolipid-anchored acetylcholinesterases from rabbit lymphocytes and erythrocytes differ in their sensitivity to phosphatidylinositol-specific phospholipase C. Biochim. Biophys. Acta.

[63] Lazar D.F., Knez J.J., Medof M.E., Cuatrecasas P., Saltiel A.R. (1994). Stimulation of glycogen synthesis by insulin in human erythroleukemia cells requires the synthesis of glycosylphosphatidylinositol. Proc. Natl. Acad. Sci. USA.

[64] Lisanti M.P., Field M.C., Caras I.W., Menon A.K., Rodriguez-Boulan E. (1991). Mannosamine, a novel inhibitor of glycosylphosphatidylinositol incorporation into proteins. EMBO J..

[65] Pan Y.-T., Kamitani T., Bhuvaneswaran C., Hallaq Y., Warren C.D., Yeh E.T.H., Elbein A.D. (1992). Inhibition of glycosylphosphatidylinositol anchor formation by mannosamine. J. Biol. Chem..

[66] Bryson H., Buttle D.J., Kozaci L.D., Johnatty R.N., Bunning R.A.D. (2000). Evidence that the inhibition of cartilage proteoglycan breakdown by mannosamine is not mediated via inhibition of glycosylphosphatidylinositol anchor formation. Biochem. J..

[67] Sandy J.D., Thompson V., Verscharen C., Garnett D. (1999). Chondrocyte-mediated catabolism of aggrecan: Evidence for a glycosyl-phosphatidylinositol-linked protein in the aggrecanase response to interleukin-1 or retinoic acid. Arch. Biochem. Biophys..

[68] Wei Y., Waltz D.A., Rao N., Drummond R.J., Rosenberg S., Chapman H.A. (1994). Identification of the urokinase receptor as an adhesion receptor for vitronectin. J. Biol. Chem..

[69] Harasymiak-Krzyzanowska I., Niedojadlo A., Karwat J., Kotula L., Gil-Kulik P., Sawiuk M., Kocki J. (2013). Adipose tissue-derived stem cells show considerable promise for regenerative medicine applications. Cell. Mol. Biol. Lett..

[70] Bertheuil N., Chaput B., Menard C., Varin A., Laloze J., Watier E., Tarte K. (2019). Adipose mesenchymal stromal cells: Definition, immune modulatory properties, mechanical isolation and interest for plastic surgery. Ann. Chir. Plast. Esthet..

[71] Müller G., Hanekop N., Wied S., Frick W. (2002). Cholesterol depletion blocks redistribution of lipid raft components and insulin-mimetic signaling by glimepiride and phosphoinositolglycans in rat adipocytes. Mol. Med..

[72] Simons K., Ikonen E. (1997). Functional rafts in cell membranes. Nature.

[73] Lingwood D., Simons K. (2010). Lipid rafts as a membrane-organizing principle. Science.

[74] Goni F.M. (2019). “Rafts”: A nickname for putative transient nanodomains. Chem. Phys. Lipids.

[75] Müller G. (2002). Dynamics of plasma membrane microdomains and cross-talk to the insulin signalling cascade. FEBS Lett..

[76] Nishijo J., Moriyama S., Shiota S., Kamigauchi M., Sugiura M. (2004). Interaction of heptakis (2,3,6-tri-O-methyl)-beta-cyclodextrin with cholesterol in aqueous solution. Chem. Pharm. Bull..

[77] Loftsson T., Magnusdottir A., Masson M., Sigurjonsdottir J.F. (2002). Self-association and cyclodextrin solubilization of drugs. J. Pharm. Sci..

[78] Cerneus D.P., Ueffing E., Posthuma G., Strous G.J., van der Ende A. (1993). Detergent insolubility of alkaline phosphatase during biosynthetic transport and endocytosis. Role of cholesterol. J. Biol. Chem..

[79] Müller G., Wied S., Crecelius A., Kessler A., Eckel J. (1997). Phosphoinositolglycan-peptides from yeast potently induce metabolic insulin actions in isolated rat adipocytes, cardiomyocytes and diaphragms. Endocrinology.

[80] Kessler A., Müller G., Wied S., Crecelius A., Eckel J. (1998). Signalling pathways of an insulin-mimetic phosphoinositolglycan-peptide in muscle and adipose tissue. Biochem. J..

[81] Müller G., Wied S., Piossek C., Bauer A., Bauer J., Frick W. (1998). Convergence and divergence of the signaling pathways for insulin and phosphoinositolglycans. Mol. Med..

[82] Müller G.A., Ussar S., Tschöp M.H., Müller T.D. (2020). Age-dependent membrane release and degradation of full-length glycosylphosphatidylinositol-anchored proteins in rats. Mech. Ageing Dev..

[83] Müller G.A., Lechner A., Tschöp M.H., Müller T.D. (2021). Interaction of full-length glycosylphosphatidylinositol-anchored proteins with serum proteins and their translocation to cells in vitro depend on the (pre-)diabetic state in rats and humans. Biomedicines.

[84] Bäckdahl J., Franzen L., Massier L., Li Q., Jalkanen J., Gao H., Andersson A., Bhalla N., Thorell A., Ryden M. (2021). Spatial mapping reveals human adipocyte subpopulations with distinct sensitivities to insulin. Cell Metab..

[85] Stenkula K.G., Erlanson-Albertsson C. (2018). Adipose cell size: Importance in health and disease. Am. J. Physiol. Regul. Integr. Comp. Physiol..

[86] Roberts R., Hodson L., Dennis A.L., Neville M.J., Humphreys S.M., Harnden K.E., Micklem K.J., Frayn K.N. (2009). Markers of de novo lipogenesis in adipose tissue: Associations with small adipocytes and insulin sensitivity in humans. Diabetologia.

[87] Ryden M., Petrus P., Andersson D.P., Medina-Gomez G., Escasany E., Corrales Cordon P., Dahlman I., Kulyte A., Arner P. (2019). Insulin action is severely impaired in adipocytes of apparently healthy overweight and obese subjects. J. Intern. Med..

[88] Medof M.E., Walter E.I., Roberts W.L., Haas R., Rosenberry T.L. (1986). Decay accelerating factor of complement is anchored to cells by a C-terminal glycolipid. Biochemistry.

[89] Davitz M.A., Hereld D., Shak S., Krakow J., Englund P.T., Nussenzweig V. (1987). A glycan-phosphatidylinositol-specific phospholipase D in human serum. Science.

[90] Davitz M.A., Low M.G., Nussenzweig V. (1986). Release of decay-accelerating factor (DAF) from the cell membrane by phosphatidylinositol-specific phospholipase C (PIPLC). Selective modification of a complement regulatory protein. J. Exp. Med..

[91] Mishra V., Heath R.J. (2021). Structural and biochemical features of human albumin essential for eukaryotic cell culture. Int. J. Mol. Sci..

[92] Fanali G., di Masi A., Trezza V., Marino M., Fasano M., Ascenzi P. (2012). Human serum albumin: From bench to bedside. Mol. Asp. Med..

[93] Merlot A.M., Kalinowski D.S., Richardson D.R. (2014). Unraveling the mysteries of serum albumin-more than just a serum protein. Front. Physiol..

[94] Ducharme N.A., Bickel P.E. (2008). Lipid droplets in lipogenesis and lipolysis. Endocrinology.

[95] Jung H.N., Jung C.H. (2021). The role of anti-inflammatory adipokines in cardiometabolic disorders: Moving beyond adiponectin. Int. J. Mol. Sci..

[96] Romero A., Eckel J. (2021). Organ crosstalk and the modulation of insulin signaling. Cells.

[97] Ferguson M.A.J., Low M.G., Cross G.A.M. (1985). Glycosyl-sn-1,2-dimyristylphosphatidylinositol is covalently linked to Trypanosoma brucei variant surface glycoprotein. J. Biol. Chem..

[98] Ferguson M.A.J., Homans S.W., Dwek R.A., Rademacher T.W. (1988). Glycosyl-phosphatidylinositol moiety that anchors Trypanosoma brucei variant surface glycoprotein to the membrane. Science.

[99] Medof M.E., Kinoshita T., Silber R., Nussenzweig V. (1985). Amelioration of lytic abnormalities of paroxysmale nocturnal hemoglobinuria with decay-accelerating factor. Proc. Natl. Acad. Sci. USA.

[100] Zalman L.S., Wood L.M., Frank M.M., Müller-Eberhard H.J. (1987). Deficiency of the homologous restriction factor in paroxysmal nocturnal hemoglobinuria. J. Exp. Med..

[101] Wilcox L.A., Ezzel J.L., Bernshaw N.J., Parker C.J. (1991). Molecular basis of the enhanced susceptibility of the erythrocytes of paroxysmal nocturnal hemoglobinuria to hemolysis in acidified serum. Blood.

[102] Cross B., Ronzon F., Roux B., Rieu J.-P. (2005). Measurement of the anchorage force between GPI-anchored alkaline phosphatase and supported membranes by AFM force spectroscopy. Langmuir.

[103] Caseli L., Masui D.C., Furriel R.P.M., Leone F.A., Zaniquelli M.E.D., Orbulescu J., Leblanc R.M. (2008). Rat osseous plate alkaline phosphatase as Langmuir monolayer—An infrared study at the air-water interface. J. Colloid Interface Sci..

[104] Ronzon F., Rieu J.-P., Chauvet J.-P., Roux B. (2006). A thermodynamic study of GPI-anchored and soluble form of alkaline phosphatase films at the air-water interface. J. Colloid Interface Sci..

[105] Suzuki K., Okumura Y. (2000). Mechanism of selective release of membrane proteins from human erythrocytes in the presence of liposomes. Arch. Biochem. Biophys..

[106] Nakamura M., Tsujii K., Katsuragi Y., Kurihara K., Sunamoto J. (1994). Taste receptor proteins directly extracted by liposome from intact epithelium of bullfrog tongue. Biochem. Biophys. Res. Commun..

[107] Okumura Y., Ishitobi M., Sobel M., Akiyoshi K., Sunamoto J. (1994). Transfer of membrane proteins from human platelet to liposomal fraction by interaction with liposomes containing an artificial boundary lipid. Biochem. Biophys. Acta.

[108] Kogure K., Nakamura C., Okuda O., Hayashi K., Ueno M. (1997). Effect of dicetylphosphate or stearic acid on spontaneous transfer of protein from influenza virus-infected cells to dimyristoylphosphatidylcholine liposomes. Biochim. Biophys. Acta.

[109] Medof M.E., Kinoshita T., Nussenzweig V. (1984). Inhibition of complement activation on the surface of cells after incorporation of decay-accelerating factor (DAF) into their membranes. J. Exp. Med..

[110] Nagarajan S., Anderson M., Ahmed S.N., Sell K.W., Selvaraj P. (1995). Purification and optimization of functional reconstitution on the surface of leukemic cell lines of GPI-anchored Fc gamma receptor III. J. Immunol. Methods.

[111] Zhang F., Schmidt W.G., Hou Y., Williams A.F., Jacobson K. (1992). Spontaneous incorporation of the glycosyl-phosphatidylinositol-linked protein Thy-1 into cell membranes. Proc. Natl. Acad. Sci. USA.

[112] Rieu J.-P., Ronzon F., Place C., Dekkiche F., Cross B., Roux B. (2004). Insertion of GPI-anchored alkaline phosphatase into supported membranes: A combined AFM and fluorescence microscopy study. Acta Biochim. Pol..

[113] Kouzayha A., Besson F. (2005). GPI-alkaline phosphatase insertion into phosphatidylcholine monolayers: Phase behavior and morphology changes. Biochem. Biophys. Res. Commun..

[114] Morandat S., Bortolato M., Roux B. (2002). Cholesterol-dependent insertion of glycosylphosphatidylinositol-anchored enzyme. Biochim. Biophys. Acta.

[115] Premkumar D.R.D., Fukuoka Y., Sevlever D., Brunschwig E., Rosenberry T.L., Tykocinski M.L., Medof M.E. (2001). Properties of exogenously added GPI-anchored proteins following their incorporation into cells. J. Cell. Biochem..

[116] Müller G., Jung C., Frick W., Bandlow W., Kramer W. (2002). Interaction of phosphatidylinositolglycan(-peptides) with plasma membrane lipid rafts triggers insulin-mimetic signaling in rat adipocytes. Arch. Biochem. Biophys..

[117] Müller G., Hanekop N., Kramer W., Bandlow W., Frick W. (2002). Interaction of phosphoinositolglycan(-peptides) with plasma membrane lipid rafts of rat adipocytes. Arch. Biochem. Biophys..

[118] Kiechle F.L., Jarett L., Kotagal N., Popp D.A. (1981). Partial purification from rat adipocyte plasma membranes of a chemical mediator which stimulates the action of insulin on pyruvate dehydrogenase. J. Biol. Chem..

[119] Saltiel A.R., Cuatrecasas P. (1986). Insulin stimulates the generation from hepatic plasma membranes of modulators derived from an inositol glycolipid. Proc. Natl. Sci. Acad. USA.

[120] Romero G., Luttrell L., Rogol A., Zeller K., Hewlett E., Larner J. (1988). Phosphatidylinositol-glycan anchors of membrane proteins: Potential precursors of insulin mediators. Science.

[121] Saltiel A.R. (1990). Second messengers of insulin action. Diabetes Care.

[122] Stralfors P. (1997). Insulin second messengers. Bioessays.

[123] Larner J., Brautigan D.L., Thorner M.O. (2010). D-Chiro-inositol glycans in insulin signaling and insulin resistance. Mol. Med..

[124] Misek D.E., Saltiel A.R. (1992). An inositol phosphate glycan derived from a Trypanosoma brucei glycosyl-phosphatidylinositol mimics some of the metabolic actions of insulin. J. Biol. Chem..

[125] Hecht M.-L., Tsai Y.-H., Liu X., Wolfrum C., Seeberger P.H. (2010). Synthetic inositol phosphoglycans related to GPI lack insulin-mimetic activity. ACS Chem. Biol..

[126] Asplin I., Galasko G., Larner J. (1993). Chiro-inositol deficiency and insulin resistance: A comparison of the chiro-inositol- and the myo-inositol-containing insulin mediators isolated from urine, hemodialysate, and muscle of control and type II diabetic subjects. Proc. Natl. Acad. Sci. USA.

[127] Chakraborty N., d’Alarcao M. (2005). An anionic inositol phosphate glycan pseudotetrasaccharide exhibits high insulin-mimetic activity in rat adipocytes. Bioorg. Med. Chem..

[128] Van Niel G., D’Angelo G., Raposo G. (2018). Shedding light on the cell biology of extracellular vesicles. Nat. Rev. Mol. Cell Biol..

[129] Rome S., Blandin A., Le Lay S. (2021). Adipocyte-derived extracellular vesicles: State of the art. Int. J. Mol. Sci..

[130] Müller G. (2012). Microvesicles/exosomes as potential novel biomarkers of metabolic diseases. Diabetes Metab. Syndr. Obes..

[131] Lakhter A.J., Sims E.K. (2015). Emerging roles for extracellular vesicles in diabetes and related metabolic disorders. Mol. Endocrinol..

[132] Medof M.E., Nagarajan S., Tykocinski M.L. (1996). Cell-surface engineering with GPI-anchored proteins. FASEB J..

[133] Bouwens E.A., Stavenuiter F., Mosnier L.O. (2015). Cell painting with an EPCR to augment the protein C system. Thromb. Haemost..

[134] Müller G.A. (2011). Oral delivery of protein drugs: Driver for personalized medicine. Curr. Issues Mol. Biol..

[135] Müller G., Wetekam E., Jung C., Bandlow W. (1997). Membrane association of lipoprotein lipase and a cAMP-binding ectoprotein in rat adipocytes. Endocrinology.

[136] Huang K., Park S., Balagurunathan K., Nakato H., Desai U., Saijoh Y. (2003). Affinity purification of glycosylphosphatidylinositol-anchored proteins by alpha-toxin. Glycosaminoglycans.

[137] Müller G. (2010). Control of lipid storage and cell size between adipocytes by vesicle-associated glycosylphosphatidylinositol-anchored proteins. Arch. Physiol. Biochem..

[138] Lawrence J.C., Guinovart J.J., Larner J. (1977). Activation of rat adipocyte glycogen synthase by insulin. J. Biol. Chem..

[139] Müller G., Jordan H., Petry S., Wetekam E.-M., Schindler P. (1997). Analysis of lipid metabolism in adipocytes using a fluorescent fatty acid derivative. I. Insulin stimulation of lipogenesis. Biochim. Biophys. Acta.

[140] Müller G., Schubert K., Fiedler F., Bandlow W. (1992). The cAMP-binding ectoprotein from Saccharomyces cerevisiae is membrane-anchored by glycosylphosphatdylinositol. J. Biol. Chem..

[141] Stieger S., Diem S., Jakob A., Brodbeck U. (1991). Enzymatic properties of phosphatidylinositol-glycan-specific phospholipase C from rat liver and phosphatidylinositol-glycan-specific phospholipase D from rat serum. Eur. J. Biochem..

[142] Li J.-Y., Hollfelder K., Huang K.-S., Low M.G. (1994). Structural features of GPI-specific phospholipase D revealed by proteolytic fragmentation and Ca2+ binding studies. J. Biol. Chem..

[143] Gnagey A.L., Forte M., Rosenberry T.L. (1987). Isolation and characterization of acetylcholinesterase from Drosophila. J. Biol. Chem..

[144] Kaya A.L., Özcan B., Sisecioglu M., Özdemir H. (2013). Purification of acetylcholinesterase by 9-amino-1,2,3,4-tetrahydroacridine from human erythrocytes. Appl. Biochem. Biotechnol..

