# Supplementary material for: Biological Role of the Intercellular Transfer of Glycosylphosphatidylinositol-Anchored Proteins: Stimulation of Lipid and Glycogen Synthesis
Source: Int J Mol Sci. 2022 Jul 4;23(13):7418. doi: 10.3390/ijms23137418 (PMC9267055; doi:10.3390/ijms23137418)
Supplement: Supplementary file 1 [file ijms-23-07418-s001.zip › ijms-1742272-supplementary.pdf]

Supplementary Materials for

Biological Role of the Intercellular Transfer of Glycosylphosphatidylinositol-Anchored Proteins: Stimulation of Lipid and Glycogen Synthesis

Günter A. Müller and Timo D. Müller

International Journal of Molecular Sciences

Materials and Methods

Materials

12-((7-nitrobenz-2-oxa-1,3-diazol-4-yl)amino)dodecanoic acid (NBD-FA) was purchased from Molecular Probes Inc. (Eugene, OR, USA). Plates for thin layer chromatography (TLC) (Si-60) and tetrahyrofurane (THF) were from Merck (Darmstadt, Germany). D-[U-^{14}C]glucose (250-360 mCi/mmol, 3% ethanol) (Cat.No. NEC042V), liquid scintillation cocktail Ultima Gold^{TM} MV (Cat.No. 6013151) and the liquid scintillation counter TRI-CARB 4810TR 110V were obtained from PerkinElmer (Waltham, MA, USA).

S1. Preparation of Rat Adipocytes of Age / Size Classes I, II and IV from Epididymal Fat Pads

Primary rat adipocytes were prepared from epididymal fat pads of male Wistar rats fed a chow diet for 3, 6 and 16 weeks with a mean body weight of 91.8 $\pm$ 4.7, 179.1 $\pm$ 8.9 and 455 $\pm$ 29.3 g (corresponding to the age / size classes I, II and IV as characterized by Müller and coworkers [S1]) by digestion with collagenase for 20 min (routine digestion) or 60 min (long digestion) as described previously [S2]. Released adipocytes were washed three times with adipocyte buffer (20 mM Hepes/KOH, pH 7.4, 140 mM NaCl, 4.7 mM KCl, 2.5 mM $CaCl_2$, 1.2 mM $MgSO_4$, 1.2 mM KH_2PO_4, 2% [*w*/*v*] BSA, 100 µg/mL gentamycin, 50 units/mL penicillin, 50 µg/mL streptomycin sulfate, 1 mM sodium pyruvate, 5.5 mM glucose, 0.5 U/mL adenosine deaminase, 200 nM phenylisopropyladenosine) by flotation (200x *g*, 2 min, 30 °C; aspiration of infranatant). After final aspiration of the infranatant, the adipocytes were suspended in adipocyte buffer at a lipocrit of 10% corresponding to 100 µL of packed cell volume per mL (determined by aspiration of small aliquots into capillary hematocrit tubes and centrifugation for 60 sec in a microhematocrit centrifuge in order to assess the fractional occupation of the suspension by the adipocytes). 10% lipocrit corresponds to about 10.5, 4.6 and 0.3 x 10^6 adipocytes per mL from rats of age / size classes I (young age / small size), II (medium age / size) and IV (old age / large size), respectively.

S2. Assay for Glycogen Synthesis with EL Cells

After serum deprivation for 4 h, the cells were washed twice with KRB containing 0.1% BSA and then resuspended in the same buffer at 7.5 x 10^5 cells/mL. Aliquots (0.4 mL each) of this suspension in 5-mL plastic vessels were then incubated (120 min, 37 °C) with 1 mM D-[U-^{14}C]glucose (4 µCi *per* aliquot) in a shaking water bath as was previously described [S3,S4] with modifications. In brief, the assay was terminated by immersion of the vessels into an ice bath (20 min) and subsequent three washings of the cells with 4 mL of ice-cold PBS, each. The last cell pellet was homogenized (0 °C, 10 up- and down-pipetting cycles with a 0.1-mL pipette) in 0.1 mL of 25 mM Tris/HCl (pH 7.4), 5 mM EDTA, 100 mM NaF, 0.1 mM PMSF. The homogenate was then centrifuged (10,000x *g*, 20 min, 4 °C). 40-µL Aliquots of the supernatant were transferred into new 2.5-mL tubes, supplemented with 20 µL of 5 mg/mL “carrier” glycogen, then heated (45 min, 100 °C) in 1 mL of 30% KOH and subsequently adjusted to 70% ethanol by addition of 1.5 mL of 100% ethanol. After incubation (4 h, -20 °C), the samples were centrifuged (2000x *g*, 15 min, 4 °C). The glycogen pellets were washed four times with 70% ethanol and then dissolved in 200 µL of distilled water. Three 60-µL portions were spotted on 2-cm^2 filter papers, which were dried before liquid scintillation counting. The amount of glucose incorporated into glycogen was calculated for the total cell samples after subtracting the radioactivity obtained from tubes to which KOH was added before cells.

S3. Assay for Lipid Synthesis with Human and Rat Adipocytes

In general, lipid synthesis with adipocytes was measured as the incorporation of 12-((7-nitrobenz-2-oxa-1,3-diazol-4-yl)amino)dodecanoic acid (NBD-FA) into total fluorescent acylglycerols. For primary rat adipocytes, the procedure was recently described in detail [S5]. For human adipocytes differentiated from hADSCs in Transwell co-culture, the medium was removed from the bottom companion tissue culture plates and replaced by 1 mL/well of 25 mM Hepes free acid, 25 mM Hepes sodium salt (pH 7.2), 80 mM NaCl, 1 mM $MgSO_4$, 2 mM $CaCl_2$, 6 mM KCl, 1 mM glucose (KRH) containing 0.75% (w/v) BSA (KRHLB). After incubation of the bottom plate (30 min, 37 °C, mild shaking [stage 11, thermomixer, Eppendorf, Germany]), the assay was initiated by the addition of 250 µL of 0.9 mM NBD-FA (prepared daily from a 100-mM stock solution in ethanol by dilution with KRHLB under mild heating). After incubation (3 h, 37 °C, as above), lipid synthesis was terminated by suction of the medium and washing of the cells three times with 2 mL/well of KRHLB. After removal of the last washing fluid, the adipocytes were dissolved and their acylglycerols were extracted by addition of 1.5 mL/well of tetrahydrofurane (THF) and rigorous shaking (20 min). Thereafter, 1.4 mL of THF was transferred into new tubes and centrifuged (15,000x *g*, 5 min). The supernatant was dried (SpeedVac) and then suspended in 50 µL THF. 5-µL samples were analyzed by thin layer chromatography (TLC) on silica gel Si-60 plates using 78 mL diethylether, 22 mL petrol ether, 1 mL acetic acid as solvent system. Fluorescent (acylglycerol) products on the dried plates

were evaluated by fluorescence imaging (Storm 860 phosphorimager, Molecular Dynamics, Krefeld, Germany) with excitation at 460 nm and emission at 540-560 nm. The relative peak area of each lipid product minus a background value (derived from an equal-sized region of the TLC plate which does not contain any lipidic product) is given in arb. units.

S4. Digestion with Phospholipases

Partially purified PI-PLC from *Bacillus cereus* (0.25 mU/mL) in PIPLC buffer (20 mM Tris/HCl, pH 7.8, containing 0.1% (*w*/*v*) BSA, 150 mM NaCl, 0.5 mM EDTA, 1 mM DTT and 0.1 mM PMSF) or GPLD1, which had been purified from bovine serum by sequential chromatography on anti-GPLD1 antibody, wheat germ lectin and monoQ columns according to Refs. [S6,S7], in 20 mM Hepes/KOH (pH 7.0), 300 mM NaCl, 2 mM Ca^{2+} at 25 ng/mL were added to incubation media or to the insert wells of transwell co-cultures at 1:200 and 1:50 dilution, respectively.

Results

Phosphoinositolglycans (PIGs) induce insulin-mimetic actions and dissociation of GPI-APs from serum proteins

During the initial evaluation of putative physiological actions of the intercellular transfer of GPI-APs, it was reasoned that PIGs and their reported effects *in vitro* could provide some relevant hints for the following reasons: (i) The structures of PIGs (see **Figure S2**) resemble that of the glycan core of authentic GPI-APs, as exemplified here for human AChE (**Figure S1**) to variable degrees [S8].

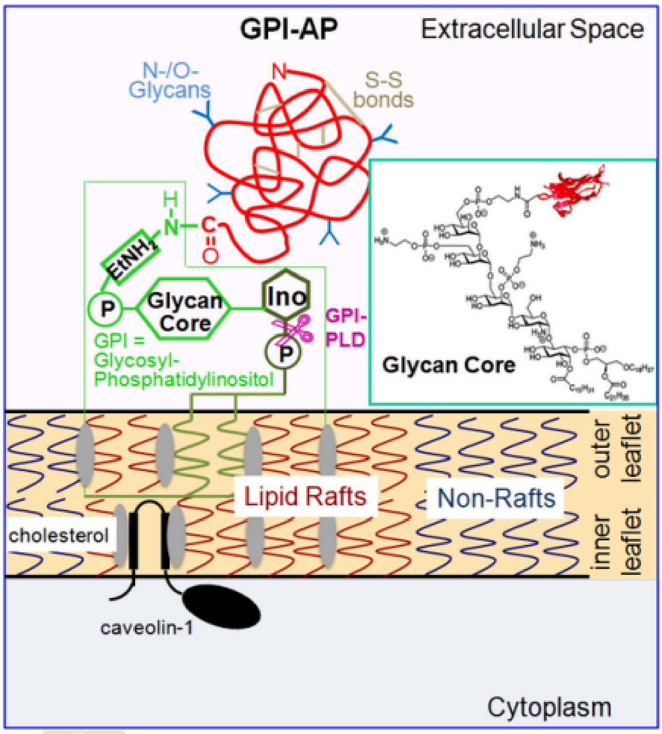

Figure S1. Structure of full-length GPI-APs, their membrane anchorage and lipolytic cleavage, and the glycan core of human AChE

GPI-APs consist of a large polypeptide domain (red), typically harboring N-/O-glycosidically linked glycan chains (blue) and intrachain disulfide bonds (brown), and a GPI anchor which is constituted by a phosphatidylinositol moiety (P-Ino) with long saturated fatty acids (dark green) and a glycan core (light green; detailed structure for AChE see inset). The carboxyl terminus of the polypeptide domain and the terminal mannose residue of the glycan core are coupled *via* an ethanolamine moiety ($EtNH_2$) through amide and phosphodiester linkages, respectively. The fatty acids of the GPI anchor are embedded in the outer leaflet of PM, typically within lipid rafts (characterized by high cholesterol content and expression of caveolin-1), with the polypeptide domain protruding into the extracellular space. The cleavage specificity of GPI-PLD is indicated (pink) separating the phosphatidic acid moiety from the inositol (Ino)-glycan protein moiety.

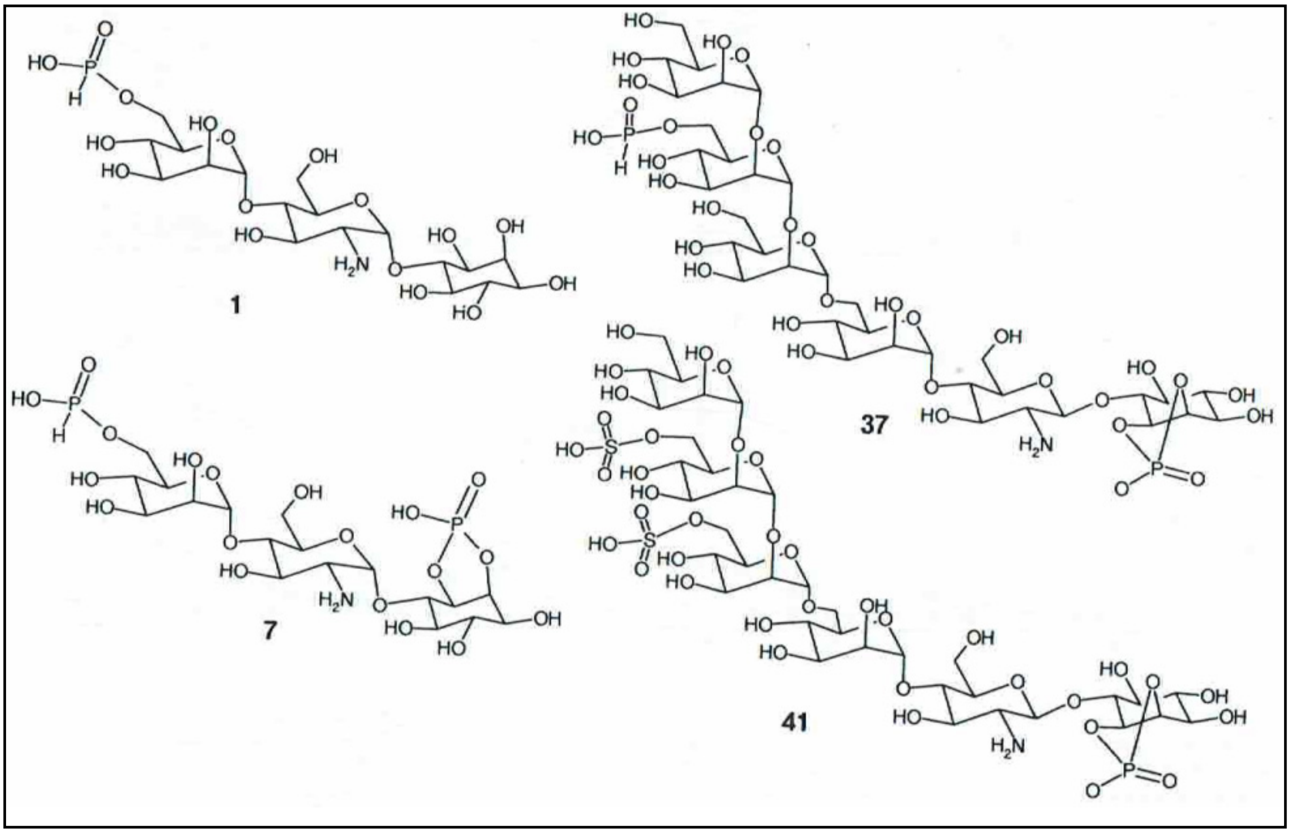

Figure S2. Structure of the PIGs used

(ii) PIG41 with high structural similarity to GPI-APs was shown to stimulate the transfer of GPI-APs from donor to acceptor adipocyte and erythrocyte PM in the presence of serum proteins as recently assayed by chip-based sensing *in vitro* [S9]. (iii) PIG41 was reported to inhibit the binding of GPI-APs to serum proteins as recently monitored by chip-based sensing *in vitro* [S10]. (iv) During the past three decades, PIGs of variable structure were demonstrated to exert insulin-mimetic effects, such as stimulation of glucose transport and the incorporation of glucose into lipids and

glycogen, in insulin target cells, such as adipose and muscle cells [S8], albeit this has remained a matter of intense dispute in studying insulin signaling and action *in vitro* (see Discussion of main text).

As initial step to substantiate the hypothetical relationship between the release of GPI-APs from serum proteins, their transfer to target cells and the induction of insulin-mimetic actions in these cells, the structure-activity relationships for these processes were assessed for five structurally different PIGs. These have previously been chemically synthesized and categorized into distinct classes of insulin-mimetic activity as exerted in cells of very high insulin sensitivity and responsiveness, primary rat adipocytes prepared from young rats [S8]: PIG41, HO-SO_2-O-6Manα1(Manα1-2)-2Manα1(6-HSO_3)-6Manα1-4GluNβ1-6(D)-inositol-1,2-(cyclic)phosphate, very high activity; PIG37, HO-PO(H)O-6Manα1(Manα1-2)-2Manα1-6Manα1-4GluNβ1-6(D)-inositol-1,2-(cyclic)-phosphate, high activity; PIG45, Manα1-2Manα1-2Manα1(6-HSO_3)-6Manα1-3GluNβ1-3(D/L)inositol-6-sulfate, medium activity; PIG7, Manα1-4GluNα1-6(L)inositol-1,2-(cyclic)-phosphate, low activity; PIG1, HO-PO(H)O-6Manα1-4GluNα1-6(L)inositol, very low activity.

In fact, incubation of primary adipocytes prepared from young rats of age class I (**Figure S2**, black lines) with PIG41 (**Figure S3b**), PIG37 (**Figure S3c**), PIG45 (**Figure S3d**), PIG7 (**Figure S3e**) and PIG1 (**Figure S3f**) led to upregulation of lipid synthesis to maximal 93, 69, 47, 26 and 19%, respectively, of the maximal insulin response (**Figure S3a**) with similar effective concentrations for half-maximal response (EC_{50} as calculated for the response towards the maximal concentration of each corresponding PIG) of 5,1, 14.1, 8.9, 10.1 and 18.2 μM compared to insulin (0.058 nM).

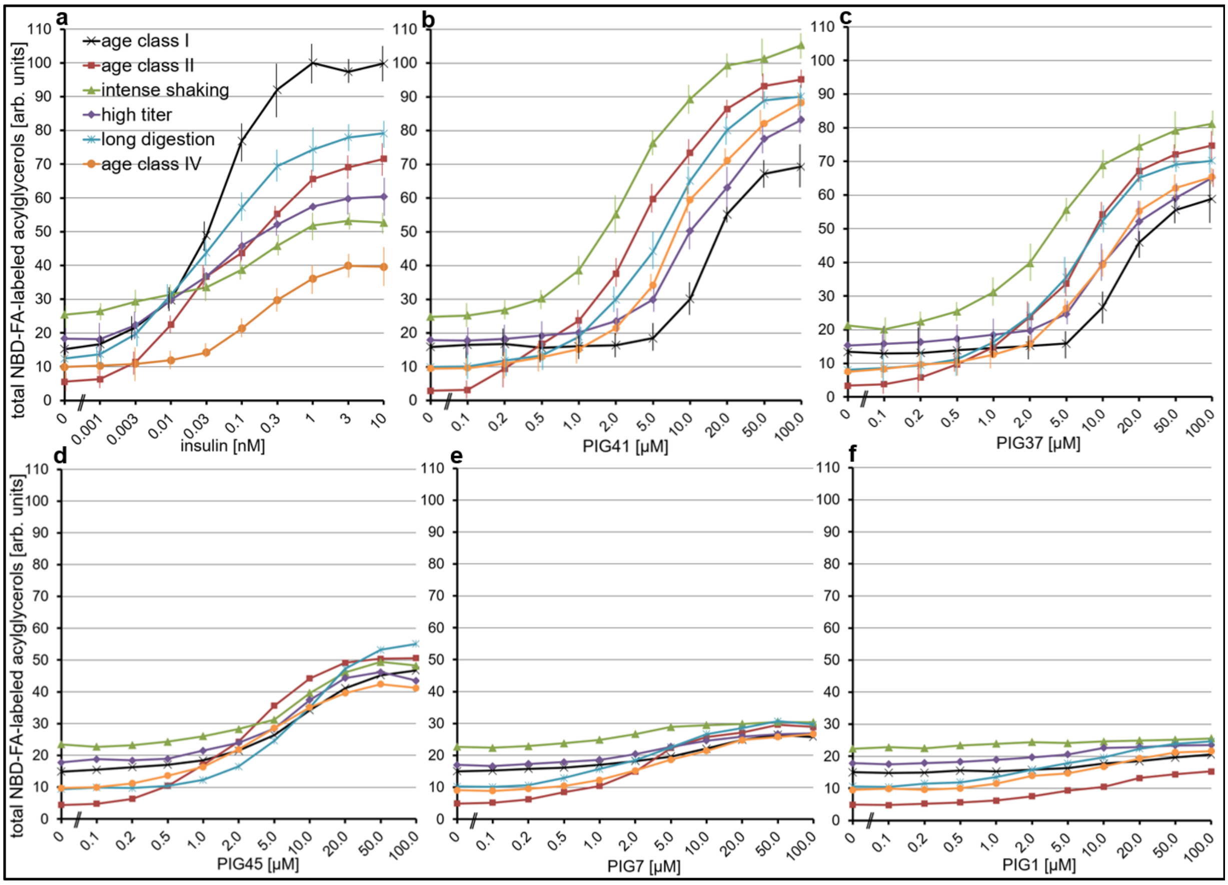

Figure S3. Stimulation of lipid synthesis by insulin and PIGs in primary rat adipocytes.

Primary adipocytes prepared from rats of age classes I (black, green, pink, blue), II (red) and IV (orange) by routine (black, red, pink, green, orange) or long (blue) collagenase digestion were suspended at a lipocrit of 0.2% (routine conditions; black, green, blue, red, orange) or 1% (high titer; pink) and then incubated (3 h, 37 °C) with NBD-FA in a shaking water bath at 60 cycles/min (routine conditions; black, pink, blue, red, orange) or 360 cycles/min (intense shaking; green) in the absence or presence of increasing concentrations of insulin (**a**), PIG41 (**b**), PIG37 (**c**), PIG45 (**d**), PIG7 (**e**) or PIG1 (**f**). NBD-FA-labeled acylglycerols were extracted from the adipocytes, separated by TLC and quantitatively evaluated by fluorescence imaging. The amount of total NBD-FA-labeled acylglycerols (set at 100 arb. units for adipocytes of age class I in response to 1 nM insulin) is given as mean ± SD (four independent incubations with independent adipocyte preparations and acylglycerol extraction/determination in triplicate, each).

As expected, the maximal insulin response (**Figure S3a**) was markedly reduced with increasing age of the rats (age class I, black line > age class II, red line > age class IV orange line), incubation with insulin at routine (black line) > intense (green) shaking, incubation with insulin at routine (black line) > high (pink line) adipocyte titer and use of adipocytes digested with collagenase for routine period (black line) > long (orange line) period. Strikingly, the impact of age, incubation and preparation of the adipocytes on the responsiveness towards PIGs, in general (i.e. this responsiveness regard was not affected by the structural differences between the PIGs), differed from that towards insulin, with medium age class II > IV > I, intense > routine shaking, high > routine titer and long > routine digestion. These findings of drastically different requirements for the induction of insulin-mimetic activity between PIGs and insulin with regard to adipocyte age (medium *vs*. young), preparation (harsh *vs*. gentle conditions) and incubation (high *vs*. low titer) suggest the involvement of different molecular mechanism(s), i.e. those being related to extracellular pathways, such as remodeling of the PM in course of ageing, collagenase digestion and cell-to-cell contact (as a result of high titer during incubation) *vs*. canonical insulin signaling, which are critically dependent on intact PM (i.e. routine shaking and digestion conditions during preparation), small cell size (i.e. young age) and low cell-to-cell contact (i.e. routine titer during incubation).

Furthermore, the effect of PIGs on lipid synthesis in rat adipocytes and their relative potency turned out to critically depend on the presence of serum during the incubation (**Figure S4**). For this, adipocytes from age class I were assayed under routine conditions (see **Figure S3**) in the presence of low (final conc. 0.015%) or high (final conc. 0.15%) BSA or (final conc. 0.15%) total serum in the incubation medium for the incorporation of NBD-FA into total acylglycerols in response to insulin or PIGs (**Figure S4a**). The low concentration of BSA left under each incubation condition was required for the binding and solubilization of NBD-FA and their efficient delivery to the cellular fatty acid uptake and lipid synthesizing machinery. The stimulation of lipid synthesis in adipocytes from young rats by insulin and PIGs of either type *vs*. control was considerably diminished (6.3- to 1.7-fold) and completely abrogated, respectively, under conditions of low BSA in the incubation medium compared to high BSA. Importantly, this loss of responsiveness was due to a more than four-fold increase in

control lipid synthesis (absence of insulin and PIGs) at low BSA. Apparently, insulin and PIGs elicited low and no further elevation, respectively, of lipid synthesis. Strikingly, the presence of total serum (at 0.15% final conc.) led to restoration of upregulation of lipid synthesis by insulin and PIGs under preservation of the ranking order in relative potency (insulin = PIG41 > PIG37 > PIG45 > PIG7 > PIG1) as measured for high BSA. Nevertheless, there was a tendency of increased control and decreased insulin-induced lipid synthesis by using total serum compared to BSA at the identical (high) concentration. Taken together, serum at a certain concentration in the incubation medium of the adipocytes is required to demonstrate the insulin-mimetic activity of PIGs which is predominantly based by keeping the control activity low rather than by increasing the PIG-dependent activity above control. This argued for an active role of serum factors in the molecular mechanism involved in the induction of insulin-mimetic activity by PIGs.

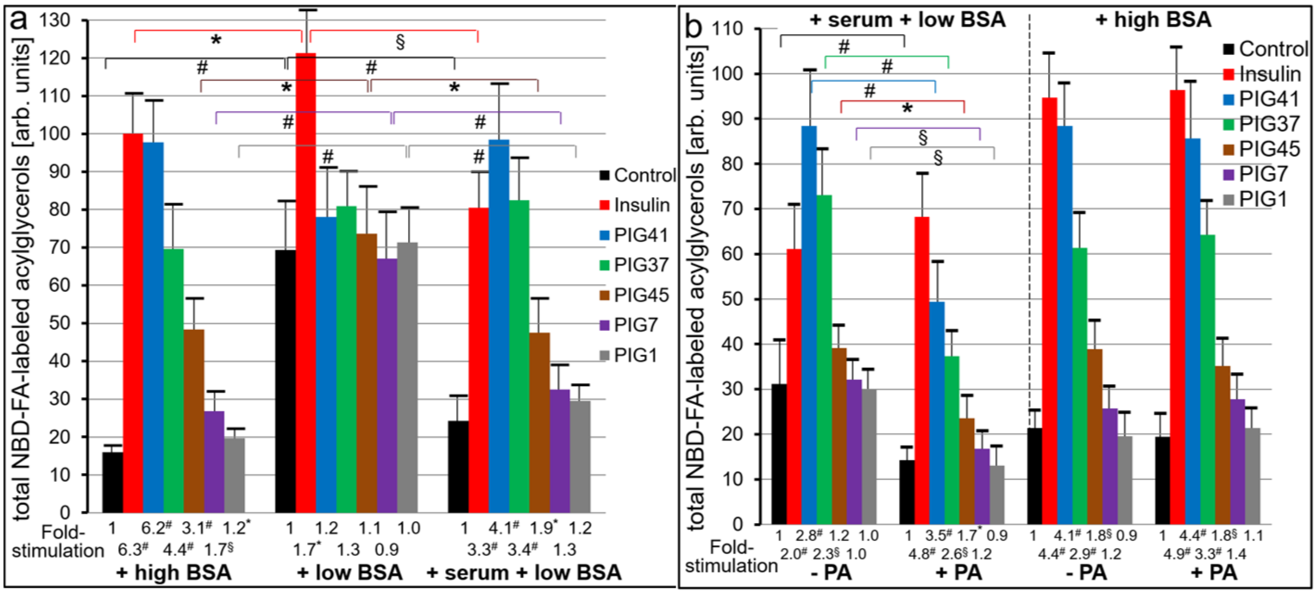

Figure S4. Effect of serum on the stimulation of lipid synthesis by insulin and PIGs in primary rat adipocytes.

(**a**) Primary adipocytes prepared from rats of age class I by low collagenase digestion were washed, suspended at a lipocrit of 0.2% in KRH containing 0.075% (low) or 0.75% (high) (w/v) BSA or 1.5% total serum proteins and 0.075% BSA (+ serum + low BSA) and then incubated (3 h, 37 °C) with NBD-FA in KRH containing the corresponding concentration of BSA in a shaking water bath at 60 cycles/min in the absence of additions (Control, black bars) or presence of 1 nM insulin (red bars) or 20 µM PIG41 (blue bars), PIG37 (green bars), PIG45 (brown bars), PIG7 (pink bars) or PIG1 (grey bars). NBD-FA-labeled acylglycerols were extracted from the adipocytes, separated by TLC and quantitatively evaluated by fluorescence imaging. The amount of total fluorescent NBD-FA-labeled acylglycerols (set at 100 arb. units for incubation with high BSA and insulin) is given as mean $\pm$ SD (three independent incubations with independent adipocyte preparations and acylglycerol extraction/determination in duplicate, each). Significant differences between control incubations, insulin incubations or incubation with PIGs of the same type performed in the absence and presence of serum proteins

are indicated ($^{\#}p \leq 0.01$, $^{§}p \leq 0.02$, $^{*}p \leq 0.05$). The fold-stimulation of lipid synthesis in response to insulin or PIGs is calculated as ratio between the amounts of total NBD-labeled acylglycerols measured for the presence of insulin or PIGs and Control and is given below the bars with significance *vs.* Control indicated.

(b) Primary adipocytes prepared from rats of age class I by low collagenase digestion were suspended at a lipocrit of 0.2% in KRH containing 0.75% (w/v) BSA (high BSA) or 1.5% total serum proteins and 0.075% BSA (+ serum + low BSA) and then incubated (3 h, 37 °C, shaking water bath at 60 cycles/min) in the absence or presence of 100 µM PA and 1 nM insulin (red bars) or 20 µM PIG41 (blue bars), PIG37 (green bars), PIG45 (brown bars), PIG7 (pink bars) or PIG1 (grey bars) as indicated. Thereafter, the adipocytes were washed by flotation two times with KRH containing low BSA in the absence of additions and then incubated (3 h, 37 °C) with NBD-FA in the same buffer. NBD-FA-labeled acylglycerols were extracted from the adipocytes, separated by TLC and quantitatively evaluated by fluorescence imaging. The amount of total fluorescent NBD-FA-labeled acylglycerols (set at 100 arb. units for incubation with high BSA and insulin in the absence of PA as derived from Figure 4a) is given as mean $\pm$ SD (four independent incubations with independent adipocyte preparations and acylglycerol extraction/determination in triplicate, each). Significant differences between control incubations, insulin incubations or incubation with PIGs of the same type performed in the absence and presence of PA are indicated for the condition of either "+ serum + low BSA" or "high BSA" ($^{\#}p \leq 0.01$, $^{§}p \leq 0.02$, $^{*}p \leq 0.05$). The fold-stimulation of lipid synthesis in response to insulin or PIGs is calculated as ratio between the amounts of total NBD-FA-labeled acylglycerol measured for the presence of insulin or PIGs and their absence (black bars, Control) and given below the bars with significance *vs*. Control indicated.

For the initial characterization of the nature of those serum factors, the above experiment was repeated with 1,10-phenanthroline (PA) (**Figure S4b**). The rationale for this was based on previous findings that one of the serum factors which interacts with full-length GPI-APs upon release from PM in competitive fashion to PIG41 is known to be GPI-specific phospholipase D (GPLD1) [S9]. GPLD1 is abundant in mammalian serum and depends on Ca^{2+} for activity [S11,S12]. Chelating of divalent cations by PA has recently been shown by chip-based sensing to enhance the interaction between GPLD1 and full-length GPI-APs as well as to diminish the insertion of the latter from micelle-like GPI-AP complexes into PM [S9]. Primary adipocytes were incubated with insulin, PIGs, serum proteins and BSA as described for **Figure S4a**, however, in the absence or presence of PA and then after intense washing assayed for the incorporation of fluorescent NBD-FA into total acylglycerols for the same period in the absence of any addition. This two-step procedure relied on the dependence of the adipocyte lipid-synthesizing machinery on divalent cations thereby masking their role in the stimulation of lipid synthesis by PIGs and GPI-APs in course of upstream processes. There was only a slight trend of impairment of upregulation of lipid synthesis by insulin or PIG41, 37, 45, 7 and 1 (in that ranking order of declining potency) in the presence of serum (+ serum + low BSA) as well as BSA (+ high BSA) for preincubation with PIGs and subsequent assay (**Figure S4b**) compared to simultaneous incubation with PIGs and NBD-FA (**Figure S4a**). This argued for maintenance of PIG action on lipid synthesis, as also reflected in its similar fold-stimulation, upon initial engagement of serum factors or BSA and subsequent removal of both PIGs and serum factors or BSA prior to start of the incorporation of NBD-FA into acylglycerols.

Strikingly, the presence of PA during incubation of the adipocytes with serum proteins (+ serum + low BSA) significantly reduced control and PIG-induced lipid synthesis, but had no effect on the insulin-stimulated one (**Figure S4b**). In contrast, the differential upregulation of lipid synthesis, as also reflected in the fold-stimulation, by PIG41, 37, 45, 7 and 1 in the presence of BSA (high BSA) was not affected by PA. These data argued for the involvement of serum factors relying on divalent cations, among them presumably GPLD1, in the stimulation of lipid synthesis of PIGs. It is tempting to speculate that stabilization of the interaction between those serum factors and full-length GPI-APs in the absence of divalent cations prevents their dissociation by PIGs and thereby causes abrogation of the insulin-mimetic activity of PIGs. These findings confirmed that the molecular mechanisms underlying the insulin-mimetic activity are completely different for PIGs and insulin.

Importantly, the remaining significant stimulation of lipid synthesis in the presence of serum and PA by PIG41, 37 and 45 (in that ranking order of declining poteny) strongly argued for the involvement of serum factors different from GPLD1 in mediating the insulin-mimetic activity of PIGs, apparently in cation-independent fashion and possibly relying on the interaction with full-length GPI-APs, such as albumin. The significant reductions of NBD-FA-labeled acylglycerols in control adipocytes by BSA and serum (**Figure S4a**) as well as by PA in the presence of serum (**Figure S4b**) indicated a role of both cation-dependent and independent serum factors in the adjustment of basal lipid synthesis, i.e. in the absence of insulin.

Consequently, the interaction of serum proteins with full-length GPI-APs, embedded in micelle-like complexes, and its interference by PIGs was studied next (**Figure S5**).

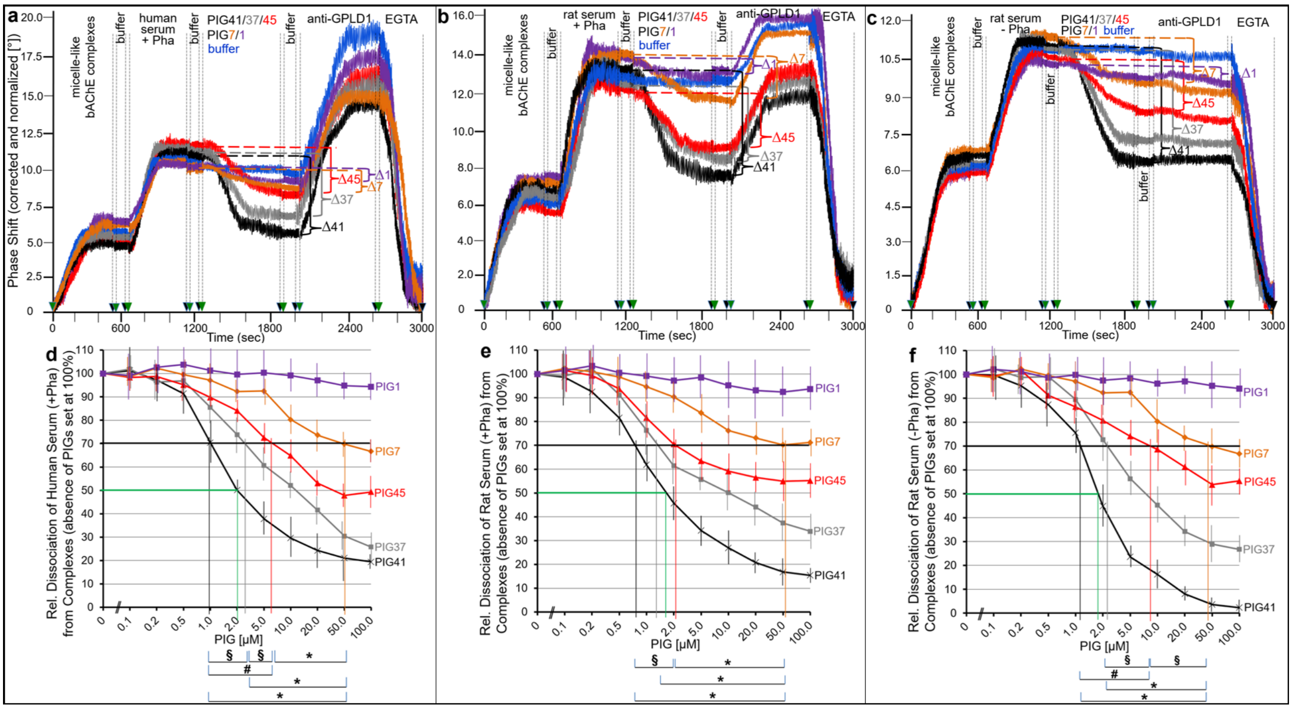

Figure S5. Displacement of rat and human serum components from micelle-like GPI-AP complexes by PIGs.

Micelle-like complexes reconstituted in the presence of bAChE at the "optimized" constituent ratio were injected into uncoated chips in the presence of Ca^{2+} (5 mM). Following washing of the chips with buffer, pooled serum from normal healthy human probands (**a**) or serum from obese ZDF rats (**b**,**c**) was injected in the presence of Pha (0.5 mM; **a**,**b**) or its absence (**c**). After washing with buffer, PIGs (30 µM; PIG41, black curves; PIG37, grey curves; PIG45, red curves; PIG7, brown curves; PIG1, pink curves) or buffer alone (blue curves) were injected, followed by additional injection of buffer, then anti-GPLD1 antibodies in the absence or presence of Pha in correspondence to the serum injection before) and finally EGTA. (**a**-**c**) Correction and normalization of phase shift were performed as described in Materials and Methods. The differences (Δ) between the maximal serum-induced phase shift left after the following washing with buffer (at 1250 sec, hatched lines) and the minimal phase shift left after PIG injection and the subsequent washing with buffer (at 2000 sec) are indicated by brackets. The experiments were repeated once (distinct chips and couplings of bAChE) with very similar results. (**d**-**f**) The experiments (0-2000 sec) were performed with increasing concentrations of PIGs (1250-1900) as described above and repeated three times (using the same chips and different preparations of micelle-like bAChE complexes) with similar results (12 regeneration cycles *per* chip). Means $\pm$ SD of the PIG-induced phase shift Δ from the three to five independent incubations were used for calculation of the relative dissociation of serum factors (**a**,**b**, presence of Pha; **c**, absence of Pha) from micelle-like bAChE complexes according to (2000 sec – 650 sec) / (1250 sec – 650 sec) and set at 100% for the absence of PIGs. The concentrations for 30% dissociation by each PIG (70% left as indicated by the thick horizontal lines) are indicated by vertical thin lines (inhibitory concentration = IC_{30}) with significant differences between the PIGs shown (* $p \leq 0.01$, $^{\#}$ $p \leq 0.02$, § $p \leq 0.05$). For PIG41, the concentration for 50% inhibition (IC_{50}) is indicated, in addition (green horizontal and vertical lines).

So far, only GPLD1, which cleaves the GPI anchor of GPI-APs, is known as a serum protein specifically interacting with GPI-APs. Since its enzymic activity critically depends on Ca^{2+} [S10] the Ca^{2+}-chelating agent, Pha, was used in order to putatively stabilize the interaction of GPI-APs and GPLD1 upon blockade of its activity. For determination of additional serum proteins associated with micelle-like bAChE complexes by chip-based sensing, the complexes were captured by the chip channels using Ca^{2+} bridges between the negatively charged TiO_2 chip surface and the negatively charged (lyso)phospholipids of the complexes as introduced previously [S10].

Injection of bAChE reconstituted into micelle-like complexes and subsequently of serum from human normal healthy probands (**Figure S5a**) or obese ZDF rats (**Figure 5b**,**c**) led to successive increases in phase shift, which both were left upon washing with buffer. Interestingly, the serum-induced phase shift increase was elevated by about 50% in the presence of Pha (**Figure S5b**) compared to its absence (**Figure S5c**). This was compatible with GPLD1 and other proteins representing the minor (about one third) and major (about two third) fraction, respectively, of serum proteins which interact with GPI-APs upon their ionic capture by the chip surface. Injection of PIGs at high concentration led to reduction in phase shift compared to buffer alone with PIG41 being most and PIG1 least effective, irrespective of the use of rat (**Figure S5b**) or human (**Figure S5a**) serum

proteins and the presence (**Figure S5a**,**b**) or absence (**Figure S5c**) of Pha. This argued for interaction of serum proteins and GPI-APs through the glycan core of their GPI anchor which is displaced by the synthetic PIGs, dependent on their structural similarity to the glycan core. Interestingly, PIG41 caused complete loss of the serum-induced phase shift increase in the absence (**Figure S5c**) but not in the presence (**Figure S5a**,**b**) of Pha. This can be explained by failure of PIGs to displace GPLD1 from micelle-like bAChE complexes, whereas interaction of GPI-APs with the other serum proteins totally depends on their glycan core and is thus sensitive towards PIGs. Injection of anti-GPLD1 antibody following the PIG action confirmed that in the presence of Pha (**Figure S5a**,**b**), GPLD1 bound to micelle-like bAChE complexes and remained bound despite the presence of PIGs. This elicited strong phase shift increases, which were independent of the type of PIG used. In contrast, the missing anti-GPLD1-induced phase shift increases in the absence of Pha (**Figure S5c**) confirmed (i) failure to detect stable interaction of micelle-like bAChE complexes and GPLD1 in the active state and (ii) displacement from micelle-like bAChE complexes by PIGs of serum proteins, only, which are different from GPLD1. Final regeneration of the chips was accomplished with EGTA which totally disrupts capture of the micelle-like bAChE complexes together with interacting serum proteins and anti-GPLD1 antibody.

For determination of the relative efficacies of the structurally different PIGs in displacing serum proteins from the micelle-like bAChE complexes, the experimental design of Figure 5a-c (0 – 2000 sec) was repeated with increasing concentrations of the PIGs. The PIG-induced phase shift differences were calculated as percentage of the serum-induced one, set at 100% for the phase shift increase from 600 to 1250 sec (**Figure S5d**-**f**). Each PIG caused concentration-dependent declines in the phase shift differences induced by human (**Figure S5d**) or rat (**Figure S5e**,**f**) serum irrespective of the presence (**Figure S5d**,**e**) or absence (**Figure S5f**) of Pha. As shown with 30 µM in the absence of Pha (**Figure S5c**), PIG41 (but not the other PIGs) managed to elicit complete dissociation of bAChE from rat serum proteins different from GPLD1 (**Figure S5f**). However, it failed to do so in the presence of Pha (**Figure S5d**,**e**) due to the interaction with GPLD1. The ranking order for the efficacy of the PIGs (PIG41 > PIG 37 > PIG45 > PIG7 > PIG1) was identical for the displacement of human (**Figure S5d**) and rat serum proteins (**Figure S5e**) as well as for serum proteins different from GPLD1 (**Figure S5f**) and GPLD1 (**Figure S5d**,**e**) as shown by the IC_{30}-values increasing from PIG41, PIG37, PIG45 to PIG7 in that order. Taken together, synthetic PIGs cause the dissociation of serum proteins, which interact with the GPI anchor of GPI-APs and are not identical with GPLD1, from GPI-APs. This is presumably due to their structural similarity to the glycan core of the GPI anchor, with PIG41 being most efficient as revealed by the IC_{50} of 1.8 µM (**Figure S5d**-**f**).

A previous study demonstrated the dependence of the release of GPI-APs from primary rat adipocytes on their size, which was positively correlated to the age of the donor animals. Therefore, the impact of cell size on the stimulation of lipid synthesis by micelle-like rat adipocyte GPI-AP

complexes was studied next (**Figure S6**). For this, adipocytes were used which had been prepared from rats of three different age classes and thus corresponded to size classes I / II / IV (small / medium / large diameter).

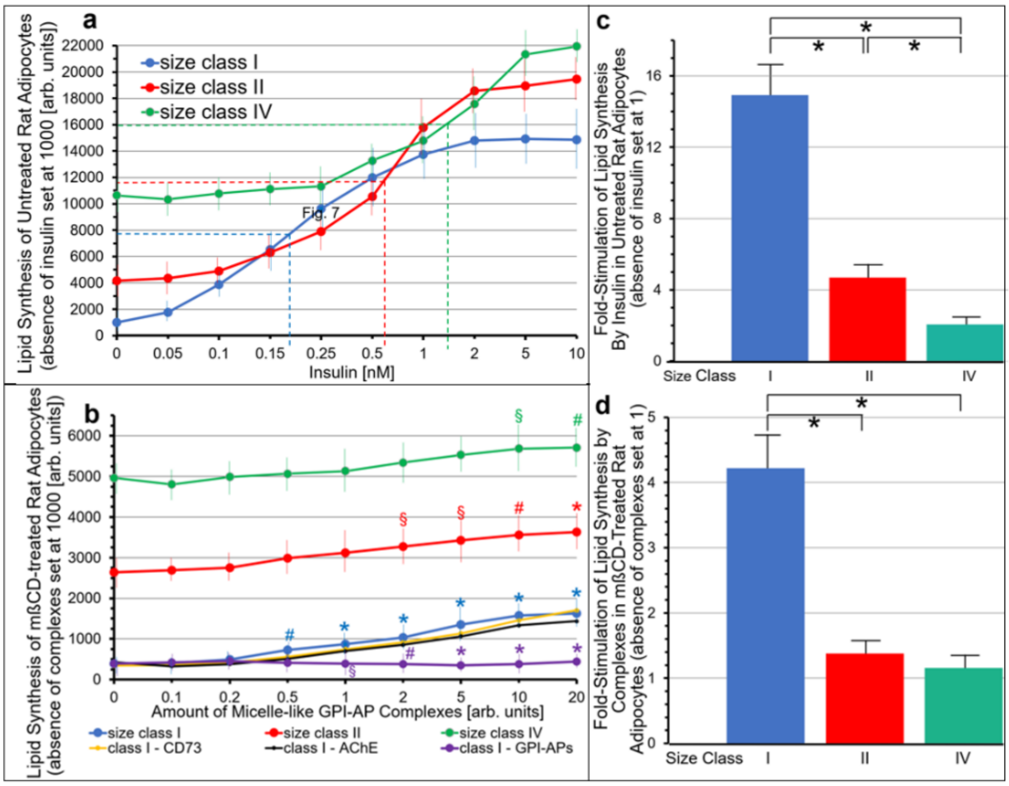

Figure S6. Effect of size of primary rat adipocytes on stimulation of lipid synthesis by micelle-like GPI-AP complexes.

Untreated (**a**,**c**) and mßCD-treated (**b**,**d**) rat adipocytes of size classes I, II and IV, prepared as described Supplementary Materials (Materials and Methods) were incubated (30 min, 37 °C) at identical lipocrit each without or with increasing amounts of (**a**) insulin or (**b**) micelle-like GPI-AP complexes reconstituted from total rat adipocyte GPI-APs (blue, red, green curves) or (for size class I only) with reconstituted complexes, which had been subsequently immune-depleted of CD73 (yellow curve) or AChE (black curve) or depleted of total GPI-APs by adsorption to α-toxin (pink curve) and then assayed for lipid synthesis as described in legend to Figure 7. (**a**,**b**) Mean values $\pm$ SD (3-5 independent incubations and assays, each) of the synthesized lipid are given as arb. units set at 1000 for incubation of untreated adipocytes of class I in the absence of insulin (**a**) or complexes (**b**). (**b**) Significant increases *vs*. absence of complexes are indicated by blue, red, green, yellow and black symbols for each size class. Significant differences between micelle-like complexes with total rat adipocyte GPI-APs (blue curve) *vs*. those depleted of GPI-APs (pink curve) for class I adipocytes are indicated by pink symbols. (**c**,**d**) Mean values $\pm$ SD of the fold-stimulation by insulin (**c**) or complexes (**d**) are given for each size class with significant differences indicated (* $p \leq 0.01$, $^{\#}$ $p \leq 0.02$, § $p \leq 0.05$).

In agreement with literature data, basal lipid synthesis (absence of insulin) was lowest for small adipocytes, followed by medium and large ones (**Figure S6a**). Consequently, both insulin sensitivity

(**Figure S6a**) and responsiveness (**Figure S6c**) were highest for small, followed by medium and then large adipocytes as reflected in the increasing apparent EC_{50} (0.19, 0.55 and 1.34 nM, horizontal and vertical hatched lines) and significantly decreasing fold-stimulations by insulin, respectively. Moreover, as manifested in the absolute increases (**Figure S6b**) and fold-stimulations (Figure 8d) of lipid synthesis, small rat adipocytes (**Figure S6b,d**, blue curve and bar) exhibited the highest responsiveness towards micelle-like GPI-AP complexes, followed by medium (**Figure S6b,d**, red curve and bar) and then large (**Figure 6b,d**, green curve and bar) cells. Again, stimulation of lipid synthesis by complexes (as shown here only for small adipocytes) turned out to rely on total rat adipocyte GPI-APs (**b**, blue and pink curves) rather than solely on CD73 (orange curve) or AChE (black curve) as revealed by adsorption of total GPI-APs to α-toxin Sepharose beads (**Figure S6b**, pink curve) and immunedepletion of CD73 or AChE (**Figure S6b**, orange and black curves), respectively. Thus, upregulation of lipid synthesis in rat adipocytes in response to transfer of total adipocyte GPI-APs depends on cell size / age of the donor animals with small size / low age being most effective.

The Transfer Efficacy Varies with Cell Type and Is Determined by the Release of GPI-APs by Donor Cells

Donor Cells	wildtype EL cells		untreated human adipocytes	
Acceptor Cells	GPI-deficient EL cells	ManN-treated adipocytes	GPI-deficient EL cells	ManN-treated adipocytes
Number of AChE *per* Donor Cell (before incubation)	3752 ± 681	2951 ± 523	748 ± 124	1035 ± 182
Number of AChE *per* Acceptor Cell (difference after and before incubation)	34 ± 8	52 ± 11	86 ± 17	79 ± 10
**Percentage of AChE in Acceptor *vs*. Donor Cells**	**0.9 ± 0.3**	**1.8 ± 0.5**	**11.5 ± 2.3**	**7.6 ± 1.8**

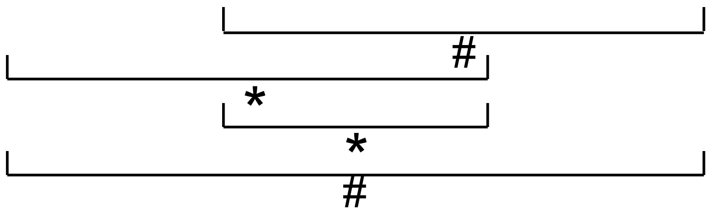

Supplementary Table S1. Efficacy of various donor-acceptor cell configurations in supporting intercellular transfer of AChE.

The experiment was performed as described for Figure 8 (main manuscript). Transwell co-cultures were run with donor and acceptor cells in the insert and bottom wells, respectively, as described in Materials and Methods in homologous configuration (wildtype and GPI-deficient EL cells; untreated and ManN-treated human adipocytes of lipid-loading stage II) or heterologous configuration (wildtype EL cells and ManN-treated human adipocytes of lipid-loading stage II; untreated human adipocytes of lipid-loading stage II and GPI-deficient EL cells). After incubation (1 week, 37 °C), PM were prepared from the cells of the bottom wells, coupled to chips by ionic/covalent capture and then analyzed for expression of the membrane proteins indicated by SAW sensing as described for Figure 1. Phase shifts induced by binding of antibodies against AChE were determined, only, with correction and normalization of the phase shift as described in the legend to Figure 1. Phase shift Δ for each incubation of acceptor with donor cells was corrected for that with medium alone in the insert wells. The number of AChE molecules expressed at donor and acceptor cells before and after incubation was determined with the aid of a set of calibration curves established for capture by the SAW sensor chips of purified human erythrocyte full-length AChE reconstituted into micelle-like (lyso)phospholipid complexes according to Refs. [S13-S15] or complexes lacking AChE but eliciting similar phase shifts as PM upon injection of antibodies against the other GPI-APs and transmembrane proteins prior to those against AChE to compensate for the non-linear and declining responsiveness of phase shift with the increase in mass loading. The experiments were repeated three to six times for the indicated configurations. Significant differences in the percentage of AChE in acceptor *vs*. donor cells (number of AChE molecules measured at PM of donor cells before incubation is set at 100%) between the various donor-acceptor cell configurations are indicated (* $p \leq 0.01$, $^{\#}$ $p \leq 0.02$).

On basis of determination of the absolute numbers of AChE molecules at the PM of donor and acceptor cells before and after incubation (for one week) of the transwell co-culture using normalized chip-based SAW sensing (with AChE purified from human erythrocytes as standard and PM derived from defined numbers of cells grown in the inset and bottom wells as well as under consideration of the non-linear response of the phase shift with the mass, i.e. GPI-APs including AChE, loaded onto the chip (in particular in case of sandwich configuration of differing complexity), the percentage of GPI-APs transferred under the experimental conditions of Figure 8 was found to reach about 1 to 10 for the various donor-acceptor cell configurations (**Supplementary Table S1**). However, this calculation of the efficacy of (heterologous) GPI-AP transfer did not consider the (presumably low) numbers of AChE molecules (i) synthesized during the 1-week incubation by the donor cells, in particular by those having released GPI-APs, (ii) being lost during the transfer process in course of unspecific adsorption to the filter membrane or plastics of the culture dishes or due to unphysiological dilution by the culture medium, and (iii) being released from the acceptor cells upon successful transfer. Nevertheless, these considerable portions of full-length GPI-APs transferred from donor to acceptor cells in the transwell co-culture further substantiated the conclusion about the biological function and physiological relevance of GPI-AP transfer *in vivo*. Furthermore, the significant differences in transfer efficacy between combinations equipped with different donor cell types (and

identical acceptor cell types) and the lack of significant differences between those with the identical donor cell types (and different acceptor cell types) are in agreement with release of full-length GPI-APs from PM of donor cells rather than their insertion into PM of acceptor cells is rate-limiting for GPI-AP transfer.

References

S1. Müller, G.A.; Ussar, S.; Tschöp, M.H.; Müller, T.D. Age-dependent membrane release and degradation of full-length glycosylphosphatidylinositol-anchored proteins in rats. *Mech. Ageing Dev*. **2020**, *190*, 111307.

S2. Müller, G. Control of lipid storage and cell size between adipocytes by vesicle-associated glycosylphosphatidylinositol-anchored proteins. *Arch. Physiol. Biochem.* **2010**, *117*, 23-43.

S3. Lawrence Jr., J.C.; Guinovart, J.J.; Larner, J. Activation of rat adipocyte glycogen synthase by insulin. *J. Biol. Chem.* **1977**, *252*, 444-450.

S4. Lazar, D.F.; Knez, J.J.; Medof, M.E.; Cuatrecasas, P.; Saltiel, A.R. Stimulation of glycogen synthesis by insulin in human erythroleukemia cells requires the synthesis of glycosylphosphatidylinositol. *Proc. Natl. Acad. Sci. U.S.A.* **1994**, *91*, 9665-9669.

S5. Müller, G.; Jordan, H.; Petry, S.; Wetekam, E.-M.; Schindler, P. Analysis of lipid metabolism in adipocytes using a fluorescent fatty acid derivative. I. Insulin stimulation of lipogenesis. *Biochim. Biophys. Acta* **1997**, *1347*, 23-39.

S6. Müller, G.; Schubert, K.; Fiedler, F.; Bandlow, W. The cAMP-binding ectoprotein from *Saccharomyces cerevisiae* is membrane-anchored by glycosylphosphatdylinositol. *J. Biol. Chem.* **1992**, *267*, 25337-25346.

S7. Müller, G.; Wetekam, E.-M.; Jung, C.; Bandlow, W. Membrane association of lipoprotein lipase and a cAMP-binding ectoprotein in rat adipocytes. *Biochemistry* **1994**, *33*, 12149-12159.

S8. Frick, W.; Bauer, A.; Bauer, J.; Wied, S.; Müller, G. Structure-activity relationship of synthetic phosphoinositolglycans mimicking metabolic insulin action. *Biochemistry* **1998**, *37*, 13421-13436.

S9. Müller, G.A.; Tschöp, M.H.; Müller, T.D. Chip-based sensing of the intercellular transfer of cell surface proteins: Regulation by the metabolic state. *Biomedicines* **2021**, *9*, 1452.

S10. Müller, G.A.; Lechner, A.; Tschöp, M.H.; Müller, T.D. Interaction of full-length glycosylphosphatidylinositol-anchored proteins with serum proteins and their translocation to cells in vitro depend on the (pre-)diabetic state in rats and humans. *Biomedicines* **2021**, *9*, 277.

S11. Stieger, S.; Diem, S.; Jakob, A.; Brodbeck, U. Enzymatic properties of phosphatidylinositol-glycan-specific phospholipase C from rat liver and phosphatidylinositol-glycan-specific phospholipase D from rat serum. *Eur. J. Biochem*. **1991**, *197*, 67-73.

S12. Li, Jau-Yi.; Hollfelder, K.; Huang, K.-S.; Low, M.G. Structural features of GPI-specific phospholipase D revealed by proteolytic fragmentation and Ca^{2+} binding studies. *J. Biol. Chem*. **1994**, *269*, 28963-28971.

S13. Gnagey, A.L.; Forte, M.; Rosenberry, T.L. Isolation and characterization of acetylcholinesterase from Drosophila. *J. Biol. Chem.* **1987**, *262*, 13920-13298.

S14. Kaya, A.L.; Özcan, B.; Sisecioglu, M.; Özdemir, H. Purification of acetylcholinesterase by 9-amino-1,2,3,4-tetrahydroacridine from human erythrocytes. *Appl. Biochem. Biotechnol.* **2013**, *170*, 198-209.

S15. Müller, G.A.; Tschöp, M.H.; Müller, T.D. Upregulated phospholipase D activity toward glycosylphosphatidylinositol-anchored proteins in micelle-like serum complexes in metabolically deranged rats and humans. *Am. J. Physiol. Endocrinol. Metab*. **2020**, *318*, E462-E479.
